# Morphology and molecular phylogeny of *Dothideomycetes* fungi associated with *Dracaena* plants

**DOI:** 10.3389/fcimb.2025.1550824

**Published:** 2025-08-11

**Authors:** Napalai Chaiwan, Kevin David Hyde, Ruvishika Shehali Jayawardena, Saowaluck Tibpromma, Dhanushka N. Wanasinghe, Ishara Sandeepani Manawasinghe, Dimuthu S. Manamgoda, Itthayakorn Promputtha

**Affiliations:** ^1^ Office of Research Administration, Chiang Mai University, Chiang Mai, Thailand; ^2^ Department of Biology, Faculty of Science, Chiang Mai University, Chiang Mai, Thailand; ^3^ Center of Excellence in Fungal Research, Mae Fah Luang University, Chiang Rai, Thailand; ^4^ Mushroom Research Foundation, School of Science, Chiang Mai, Thailand; ^5^ School of Science, Mae Fah Luang University, Chiang Rai, Thailand; ^6^ Center for Yunnan Plateau Biology Resources Protection and Utilization, College of Biology and Food Engineering, Qujing Normal University, Qujing, Yunnan, China; ^7^ Department of Soil Science, College of Food and Agriculture Sciences, King Saud University, Riyadh, Saudi Arabia; ^8^ Innovative Institute for Plant Health, Zhongkai University of Agriculture and Engineering, Guangzhou, China; ^9^ Department of Botany, University of Sri Jayewardenepura, Nugegoda, Sri Lanka

**Keywords:** fungal biodiversity, *Dothideomycetes*, drought tolerance plant, multi-loci phylogenetic analysis, new fungal taxa

## Abstract

*Dracaena* species are widely recognized for their exceptional drought tolerance, making them ideal candidates for sustainable landscaping and ecological restoration in arid regions. Limestone outcrops hosting *Dracaena* are unique ecosystems characterized by extreme environmental conditions such as nutrient-poor substrates. Thus, they provide valuable opportunities for studying fungal diversity and their adaptations. Despite their ecological importance, knowledge concerning fungal communities associated with limestone-inhabiting *Dracaena* species remains limited, particularly within the diverse biogeographic contexts of Thailand. Microfungal samples were collected from dead wood and leaves of Dracaena species across seven provinces in Thailand (Chiang Rai, Kanchanaburi, Krabi, Nakhon Si Thammarat, Ratchaburi, Songkhla, and Tak). Fungal taxa were identified and characterized through detailed morphological examinations combined with multi-gene phylogenetic analyses using Actin (act), Internal transcribed spacer (ITS), the large subunit of nuclear ribosomal RNA (LSU), translation elongation fac-tor 1-alpha (tef1-α), and beta-tubulin (tub) gene regions. This study documents eleven fungal taxa isolated from *Dracaena* substrates, belonging to seven families across five fungal orders. Three new species *viz*. *Cladosporium dracaenae*, *C. dracaenicola* and *Torula dracaenae* were described, and eight new host records were established (*Bipolaris coffeana*, *Curvularia lunata*, *Lasiodiplodia bruguierae*, *L. lignicola*, *L. thailandica*, *Longididymella clematidis*, *Ochroconis musae* and *Zasmidium citrigriseum*). Species de-scriptions, color photographic plates, phylogenetic trees and updated taxonomic notes are provided for all isolated taxa. The findings advance the current understanding of microfungal diversity associated with limestone outcrop habitats and *Dracaena* species, contributing to broader ecological and conservation efforts. By revealing novel fungal species and previously undocumented host-fungus interactions, this study underscores the rich but underexplored fungal biodiversity of limestone ecosystems in Thailand.

## 1 Introduction

Dothideomycetes is the largest and most recognized class of ascomycete fungi, renowned for its ecological diversity and significance in taxonomic and biological research (Hongsanan et al., 2020). This class includes a wide array of taxa such as endophytes, epiphytes, saprobes, pathogens of humans and plants, lichens, lichenicolous fungi, nematode-trapping fungi, and rock-inhabiting fungi (Hongsanan et al., 2020; Li et al., 2023). Dothideomycetes encompasses over 32 orders, 191 families, 1,495 genera, and more than 19,000 species (Pem et al., 2024). Characteristically, members of this class possess bitunicate asci with fissitunicate dehiscence and are found on a variety of hosts in terrestrial habitats (Hongsanan et al., 2020; Pem et al., 2024). Recent discoveries of numerous novel species, genera, families, and orders have significantly expanded the classification and understanding of Dothideomycetes.

*Dracaena*, a genus within the *Agavaceae* family of monocotyledonous plants, includes 198 species (POWO, 2021; van Kleinwee et al., 2022). These plants are distributed across Africa, Madagascar, the Arabian Peninsula, Indian Ocean islands, South Asia, Southeast Asia, Australia, Micronesia, Hawaii, Mexico, Central America, northwestern South America, Cuba, and Macaronesia (Mabberley, 2008). In Europe and Canada, *Dracaena* species are valued for their ornamental and medicinal uses (Almaghrebi et al., 2024). *Dracaena marginata* is a popular houseplant known for its ability to reduce formaldehyde levels in the air (Jaminson, 2012). Despite the fact that this species is an ecologically important plant species, studies on fungi associated with *Dracaena* species are not well established.

Ascomycetes can be found on *Dracaena* plants as pathogens, endophytes, or saprobes (Thongkantha et al., 2008; Crous et al., 2015; Lin et al., 2016; Chaiwan et al., 2019). Notably, *Dracaena* species exhibit a rich fungal diversity, as illustrated by Thongkantha et al. (2008), who documented 127 saprobic fungal records from *Dracaena* and *Pandanus* in Thailand. These taxa comprised 40 Ascomycota and one Basidiomycota, including 86 asexual morphic taxa. Of these documented fungi, eight sexual species and three asexual morphic taxa were newly described species. However, since 2010, investigations into microfungi associated specifically with *Dracaena* have been limited (Ariyawansa et al., 2015; Lin et al., 2016; Chaiwan et al., 2021). Therefore, this study aims to investigate and identify microfungi associated with *Dracaena* species in Thailand by combining morphological characteristics and molecular phylogenetic analyses, providing detailed descriptions and illustrations.

## 2 Materials and methods

### 2.1 Sample collection, morphological studies, and isolation

Specimens were collected from *Dracaena* plants in seven provinces: Chiang Rai, Kanchanaburi, Krabi, Nakhon Si Thammarat, Ratchaburi, Songkhla, and Tak of Thailand. Each sample was placed in a separate envelope or plastic bag and taken to the laboratory for observation and fungal isolation. Samples were examined using a Nikon ECLIPSE 80i compound light microscope, and micromorphological structures (fruiting bodies or mycelium from culture) were photographed with a Canon EOS 600D digital camera attached to the microscope. Measurements were conducted using the Tarosoft^®^ Image Framework program. Figures were processed with Adobe Photoshop CS6 Extended version 10.0 (Adobe Systems, USA). Specimens were isolated using single spore techniques (Senanayake et al., 2020). The sample was isolated on potato dextrose agar (PDA) plates and incubated at 25°C. Germinating spores were aseptically transferred to fresh PDA plates and incubated at 25°C. Cultures were grown for 2–4 weeks, during which morphological characteristics such as color, colony appearance, and texture were recorded. The specimens and living cultures were deposited in the Fungarium of Mae Fah Luang University (MFLU) and the Culture Collection of Mae Fah Luang University (MFLUCC) in Chiang Rai, Thailand. Facesoffungi and Index Fungorum numbers were obtained as described by Jayasiri et al. (2015) and Index Fungorum (Index Fungorum, 2025). New taxa were established based on the recommendations outlined by Jeewon and Hyde (2016); Chethana et al. (2021), and Jayawardena et al. (2021).

### 2.2 DNA extraction, PCR amplification, and sequencing

Spore mass or fruiting bodies or mycelium were used to extract DNA. The Biospin Fungus Genomic DNA Extraction Kit BioFlux (BioFlux^®^, Hangzhou, China) was used to extract DNA from fruiting bodies or spore mass. PCR amplification was performed using primer pairs, ACT512F/ACT783R for the actin (*act*) gene following the method described by Carbone and Kohn (1999), ITS4/ITS5 for the internal transcribed spacer region of ribosomal DNA (ITS), LR0R/LR5 for large subunit nuclear ribosomal DNA (LSU), *tef1-α*-983F/EF-2218R for translation elongation factor 1-alpha gene (*tef1-α*), and Bt2a/Bt2b for β-tubulin (*tub*) following the method described by Dissanayake et al. (2020). The genes were amplified using universal primers (**Supplementary Table 1**). The PCR mixer comprises 1 µl forward primer, 1 µl reverse primer, 9.5 µl distilled deionized (DD) water, and 12.5 µl mixer. The PCR conditions for ITS, LSU, and *tef1-α* were 3 min at 94°C, followed by 35 cycles of 30 s at 94°C, 50 s at 55°C, and 90 s at 72°C, and a final elongation step at 72°C for 10 min. The conditions for *act* were an initial elongation step of 2 min at 95°C, followed by 35 cycles of 45 s at 95°C, 45 s at 55°C, and 1 min at 72°C, and a final elongation step of 10 min at 72°C. Conditions for *tub* were an initial 8 min of 95°C, followed by 35 cycles of 30 s at 95°C, 30 s at 55°C, and 1 min at 72°C, and a final elongation step at 72°C for 5 min. The PCR products were purified and sequenced at Shanghai Sangon Biological Engineering Technology and Service Co. The PCR products were purified and sequenced using the same primers for each gene region. The amplification reactions followed the method described by Chaiwan et al. (2019). The quality of PCR products was assessed using 1% agarose gel electrophoresis stained with ethidium bromide. The PCR products were sent for sequencing at Sangon Biotech in Kunming, China.

### 2.3 Sequence assembly, alignment, and phylogenetic analyses

All sequences obtained from each strain were used for BLAST searches in the nucleotide database of GenBank (http://blast.ncbi.nlm.nih.gov/) to determine their closest taxa. The phylogenetic positions of Dothideomycetes isolates were initially assessed using sequence data from *act*, ITS, LSU, *tef1-α*, and *tub* genes. Species-level identifications within each group were then determined using multigene phylogenetic analysis based on individual and combined gene regions. Reference sequences and outgroups for each fungal group were selected from recent literature and GenBank data [https://blast.ncbi.nlm.nih.gov] (NCBI, 2025). Individual genomic region datasets were aligned separately using the MAFFT version 7.221 server (http://mafft.cbrc.jp/alignment/software/). Phylogenetic trees were inferred using maximum parsimony (MP), maximum likelihood (ML), and Bayesian inference (BI). Maximum parsimony analyses were performed with PAUP version 4.0 b10 (Swofford, 2003), excluding uninformative characters and treating all informative characters as unordered and of equal weight. Goodness-of-fit values, including tree length (TL), consistency index (CI), retention index (RI), homoplasy index (HI), and rescaled consistency index (RC), were calculated. Statistical support for branches was assessed using maximum parsimony bootstrap (MPBS) analysis with 1,000 replicates (Felsenstein, 1985). Maximum likelihood analysis was performed online using The CIPRES Science Gateway v. 3.3 (http://www.phylo.org/portal2/; Miller et al., 2010) and RAxML v. 7.2.8 (Stamatakis et al., 2008) as part of the BRAxML–HPC BlackBox on TG tool were used. Phylogeny website tool BALTER (http://sing.ei.uvigo.es/ALTER/) (Glez-Peña et al., 2010) was used to transfer the alignment file for RAxML analysis. All free model parameters were estimated by RAxML and ML estimated 25 per site rate categories. The model selected for ML was GTRGAMMA. The CIPRES Science Gateway v. 3.3 (http://www.phylo.org/portal2/; Miller et al., 2010) was used as part of MrBayes on XSEDE on the TG tool. Phylogeny website tool BALTER (http://sing.ei.uvigo.es/ALTER/) (Glez-Peña et al., 2010) was used to transfer the alignment file for MrBayes analysis. Phylogenetic trees were visualized and annotated using TreeView (Page, 1996) and formatted using PowerPoint 2010 (Microsoft Corporation, WA, USA).

### 2.4 Genealogical concordance phylogenetic species recognition analysis

The related species were analyzed using the Genealogical Concordance Phylogenetic Species Recognition (GCPSR) analysis model. This model assesses significant recombinant events to determine recombination levels within closely related species and supports the significance of new species in a clade. The pairwise homoplasy index (PHI) test, which evaluates the concordance and discordance of multiple gene phylogenies due to recombination and mutations, was performed using SplitsTree4, as described by Quaedvlieg et al. (2014). The analysis, conducted with a locus concatenated dataset, identified significant recombination if the PHI value was below the 0.05 threshold (Фw < 0.05), indicating that the recombination levels among related species were not different. Conversely, a PHI value above 0.05 (Фw > 0.05) suggested no significant recombination, implying differences among related species in the group. The relationships between closely related species were visualized by constructing a split graph using both the LogDet transformation and split decomposition options.

## 3 Results

**Dothideomycetes** O.E. Erikss. & Winka

**Botryosphaeriales** C.L. Schoch, Crous & Shoemaker

Botryosphaeriales was originally introduced to include accommodate *Botryosphaeriaceae* (Schoch et al., 2009). Subsequently, the order was expanded to encompass six families: *Aplosporellaceae, Botryosphaeriaceae, Melanopsaceae, Phyllostictaceae, Planistromellaceae*, and *Saccharataceae* (Phillips et al., 2019; Hongsanan et al., 2020, Wijayawardene et al., 2022).

***Botryosphaeriaceae*** Theiss. & H. Syd.

Wijayawardene et al. (2022) recognized 22 genera within *Botryosphaeriaceae*. Species in this family are diverse, functioning as endophytes, pathogens, and saprobes across a wide range of hosts, including woody plants (Crous et al., 2004; Slippers and Wingfield, 2007; Mehl et al., 2013; Trakunyingcharoen et al., 2014, Doilom et al., 2016, Manawasinghe et al., 2016). They are reported on numerous hosts and have a broad geographical distribution (Chethana et al., 2016). The family includes over 100 species and 22 genera (Hongsanan et al., 2020), with several species being significant pathogens of economically important crops, including commercial plantation tree species (Jayawardena et al., 2019). Notably, species of *Lasiodiplodia* are well-known dieback pathogens affecting woody hosts globally (Manawasinghe et al., 2016). For example, *Lasiodiplodia theobromae* causes dieback and wood discoloration in various tropical plants, such as *Acacia mangium* in Indonesia (Slippers and Wingfield, 2007; Jayawardena et al., 2020). However, there is currently no information on *Botryosphaeriaceae* affecting *Dracaena* sp. In this study, we introduce three new host records from *Dracaena* sp. from Thailand.

***Lasiodiplodia*** Ellis & Everh.

*Lasiodiplodia* is a widespread genus belonging to Botryosphaeriaceae (Alves et al., 2008; Phillips et al., 2019; Wijayawardene et al., 2022). These species have been recorded from many hosts, reporting as plant pathogens (Abdollahzadeh et al., 2010) causing fruit rot, stem-end rot, and dieback on a wide range of hosts. Species of this genus are reported as endophytes (Slippers and Wingfield, 2007, Chen et al., 2015) and saprobes (Abdollahzadeh et al., 2010, Liu et al., 2012, Dissanayake et al., 2016, Hyde et al., 2019) as well. In the present study, phylogenetic analysis of *Lasiodiplodia* was based on combined ITS and *tef1-α* sequence data of selected *Botryosphaeriaceae* isolates. Related sequences were obtained from GenBank. There were 141 taxa are included in the analyses, which comprise 1,691 characters including gaps. The tree is rooted in *Diplodia seriata* and *Diplodia mutila* best-scoring RAxML tree with a final likelihood value of − 5,535.293762. The matrix had 472 distinct alignment patterns, with 50.17% of undetermined characters or gaps. Estimated base frequencies were as follows; A = 0.215775, C = 0.287336, G = 0.258654, T = 0.238234; substitution rates AC = 1.014675, AG = 4.025490, AT = 1.480709, CG = 1.199950, CT = 5.465426, GT = 1.000000; gamma distribution shape parameter *α* = 0.223674. Maximum likelihood bootstrap support values ≥ 60% (ML) and Bayesian posterior probabilities (PP) ≥ 0.90 are given in the nodes. The scale bar indicates 0.02 changes. The isolates obtained in this study are in red, and ex-type taxa are in black bold (**Supplementary Figure 1**). Three collections from our study clustered with *Lasiodiplodia bruguierae, L. lignicola*, and *L. thailandica.*

***Lasiodiplodia bruguierae*** J.A. Osorio, Jol. Roux & Z.W. de Beer, in Osorio, Crous, De Beer, Wingfield & Roux, Fungal Biology 121(4): 374 (2016) (**Supplementary Figure 2**)

Index Fungorum number: IF188476

*Saprobic* on dead leaves of *Dracaena fragrans*. Sexual morph: undetermined. Asexual morph: *Conidiomata* 150–200 × 146–200 μm ( $\bar{\text{x}}$ = 175×173 μm; n = 10), pycnidial, semi-immersed, unilocular, solitary, scattered, globose or subglobose, dark brown. *Conidiomatal wall* 20–30 μm wide, outer layers dark brown to black, thick-walled, inner layer thin-walled, pale brown to hyaline, comprising two to three layers of dark- brown cells of *textura angularis*. *Paraphyses* up to 40 μm long, 2–3 μm wide, hyaline, septate, cylindrical, occasionally branched, ends rounded. *Conidiogenous cells* 19–21 × 3–6 μm ( $\bar{\text{x}}$ = 20 × 4.5 μm; n= 15), holoblastic, hyaline, cylindrical. *Conidia* 20–30 × 12–13 μm ( $\bar{\text{x}}$ = 25×12.5 μm; n=30), initially hyaline and aseptate when immature, becoming 1-septate at the median, dark brown, thin-walled, ellipsoid to obovoid, base truncate or rounded.

***Culture characteristics*:** Conidia germinating on PDA within 4–5 h. Colonies growing on PDA reaching 45 mm diam. After 1 day at 25°C in the dark, initially whitened in a few days, after 7 days becoming gray, fast growing, raised, fluffy, dense, filamentous, reaching the edge of the petri dish after 2 days.

***Material examined:*** Thailand, Chumphon Province, Phato District, Pang Wan, on dead leaves of *Dracaena fragrans*, 2 September 2017, Napalai Chaiwan, NCCP1, (MFLU 22–0183), living culture MFLUCC 18–1117.

***Known distribution*:** *Bruguiera gymmorrhiza* (South Africa) (Osorio et al., 2016, Dou et al., 2017), and dead leaves of *Dracaena fragrans* — (This study).

***GenBank accession number:*** ITS: OM919717, *tef1-α*: OP099910.

***Notes:*** Phylogenetic analyses show that *L. bruguierae* (MFLUCC 18–1117) is closely related to *L. bruguierae* (CMW41470, CMW41614, CMW42480). *Lasiodiplodia bruguierae* was first isolated from asymptomatic branches of *Bruguiera gymnorrhiza* in South Africa (Osorio et al., 2017). In comparison of the nucleotide variation in ITS of our collection with those of *L. bruguierae*, 1 out of 594 (0.17%) (position 51 our species showed A but *L. bruguierae* showed T) revealed nucleotide differences, whereas the *tef1-α* nucleotides showed no nucleotide differences. Based on these, we introduce our collection as a new host record from *Dracaena fragrans.*

***Lasiodiplodia lignicola*** (Ariyaw., Jian K. Liu & K.D. Hyde) A.J.L. Phillips, A. Alves & Abdollahz., in Phillips, Alves, Abdollahzadeh, Slippers, Wingfield, Groenewald & Crous, Stud. Mycol. 76: 120 (2013). (**Supplementary Figure 3**)

Index Fungorum number: IF 559796

*Saprobic* on dead leaves of *Dracaena fragrans*. Sexual morph: undetermined. Asexual morph: *Conidiomata* 200–250 × 100–130 μm ( $\bar{\text{x}}$ = 225 × 115 μm; n = 10), pycnidial, semi-immersed, unilocular, solitary, scattered, globose or subglobose, dark brown. *Paraphyses* arising from the onidiogenous layer, initially aseptate becoming up to 1-septate when mature, hyaline, tip rounded unbranched. *Conidiomatal wall* 25–40 μm wide, outer layer dark brown to black, thick-walled, inner layer, pale brown to hyaline, thin-walled comprising two to three layers of dark- brown cells of *Textura angularis*. *Conidiogenous cells* 18–22 × 6–9 μm ( $\bar{\text{x}}$ = 20 × 7.5 μm; n = 15), holoblastic, hyaline, cylindrical. *Conidia* 10–20 × 5–10 μm ( $\bar{\text{x}}$ = 17.5 × 9.5 μm; n = 30), initially hyaline and aseptate when immature, becoming medianly 1-euseptate, dark brown, thin-walled, ellipsoid to obovoid, base truncate or rounded, with longitudinal striations from apex to base.

***Material examined*:** Thailand, Krabi Province, on dead leaves of *Dracaena fragrans*, 21 December 2018, Napalai Chaiwan, Krabi1, (MFLU 22–0127).

***Known distribution*:** Dead wood (Thailand), Woody branch (China) (Liu et al., 2012; Wang et al., 2019; Wu et al., 2021), and dead leaves of *Dracaena fragrans* — (This study).

***GenBank accession number*:** ITS: OM919716, *tef1-α*: OP169686

***Notes*:** We were unable to obtain a culture because the spores of the species did not germinate. Phylogenetic analyses show that *L. lignicola* (MFLU 22-0127) is closely related to *L. lignicola* (CBS134112) (**Supplementary Figure 1**). The morphology of this species resembles *L. lignicola* in having thin-walled paraphyses and holoblastic conidiogenous cells (Liu et al., 2012; Wang et al., 2019). However, the conidia of *L. lignicola* (CBS134112) (15–17.5 × 8–11 μm) are larger than our new host record (10–20 × 5–10 μm). A comparison of the ITS nucleotides of *L. lignicola* (CBS134112) with those of this new host record revealed 6 out of 631 (0.95%) nucleotide differences. This new host record represents the first reported instance on *Dracaena fragrans* in Thailand.

***Lasiodiplodia thailandica*** T. Trakunyingcharoen, L. Lombard & Crous, Persoonia 34: 95 (2015) (**Supplementary Figure 4**)

Index Fungorum number: IF510941

*Saprobic* on dead leaves of *Dracaena fragrans.* Sexual morph: undetermined. Asexual morph: *Conidiomata* 200–300 × 150–250 μm ( $\bar{\text{x}}$ = 250 × 200 μm; n = 10), pycnidial, semi-immersed, unilocular, solitary, scattered, globose or subglobose, dark brown. *Conidiomata wall* 50–65 μm wide, outer layers dark brown to black, thick-walled, inner layers thin-walled, pale brown to hyaline, comprising two to three layers of dark- brown cells of *textura angularis*. *Paraphyses* up to 40 μm long, 1.5–2.5 μm wide, hyaline, septate, cylindrical, occasionally branched, ends rounded. *Conidiogenous cells* 19–33 × 5–10 μm ( $\bar{\text{x}}$ = 26 × 7.5 μm; n= 10), holoblastic, hyaline, cylindrical. *Conidia* 30–50 × 10–15 μm ( $\bar{\text{x}}$ = 40 × 12.5 μm; n=30), initially hyaline and aseptate when immature, becoming medianly one septate, dark brown, thick-walled, ellipsoid to obovoid, base truncate or rounded, with longitudinal striations from apex to base.

***Material examined:*** Thailand, Kanchanaburi Province, Sangkhla Buri District, on dead leaves of *Dracaena fragrans*, 24 October 2018, Napalai Chaiwan, KAN3 (MFLU 22-0146), *ibid* KLV2 (MFLU 22-0147).

***Known distribution*:** *Acacia confuse*, *Albizia chinensis*, *Magnolia candollii*, *Podocarpus macrophyllus* (China) (Dou et al., 2017; de Silva et al., 2019), *Mangifera indica* (Thailand) (Trakunyingcharoen et al., 2014) and dead leaves of *Dracaena fragrans* — (This study).

***GenBank accession numbers:*** KAN3; ITS: OM919715. KLV2; ITS: ON000546, *tef1-α*: 269 OP099911.

***Notes:*** The morphology of this collection is similar to *L. pseudotheobromae* (Gomdola et al., 2020), although it features septate paraphyses and slightly larger conidia (22–33 × 13–15 μm vs. 30–50 × 10–15 μm) (Gomdola et al., 2020; Pipattanapuckdee et al., 2019). The phylogenetic analyses (**Supplementary Figure 1**) show that our isolates cluster with the ex-type strain of *L. thailandica*. *Lasiodiplodia thailandica* was first described from symptomless twigs of *Mangifera indica* in Thailand (Trakunyingcharoen et al., 2014) and has also been reported on *Albizia chinensis*, *Podocarpus macrophyllus* (Dou et al., 2017), and *Magnolia candollii* (de Silva et al., 2019). Based on these, we introduce our collection as *L. thailandica* as a new host record from *Dracaena fragrans* from Thailand.

**Cladosporiales** Abdollahz. & Crous

Cladosporiales was introduced based on phylogenetic analyses by Abdollahzadeh et al. (2020), with *Cladosporiaceae* as the only family in this order. Initially, *Cladosporiaceae* was classified under Capnodiales but was later reassigned to *Cladosporiales* based on subsequent phylogenetic analyses (Abdollahzadeh et al., 2020; Hongsanan et al., 2020, Wijayawardene et al., 2022). Members of this order are commonly found as saprobic, endophytic, fungicolous, lichenicolous, and can act as pathogens in humans and plants (Torres-Cortés et al., 2015, Wijayawardene et al., 2022).

***Cladosporiaceae*** Chalm. & R.G. Archibald

Hyde et al. (2024) accepted eight genera in *Cladosporiaceae; Acroconidiella, Cladosporium, Davidiellomyces, Graphiopsis, Neocladosporium, Rachicladosporium, Toxicocladosporium* and *Verrucocladosporium*. They are reported on a wide range of hosts as endophytes, pathogens, and saprobes (Sandoval-Denis et al., 2016; Jayawardena et al., 2020). Members of *Cladosporiaceae* are commonly found on soil, food, paint, textiles, and other organic matters or colonize as secondary invaders leaf lesions caused by plant pathogenic fungi and common fungal components isolated from the air (Bensch et al., 2010).

***Cladosporium*** Link

*Cladosporium* is a cosmopolitan genus isolated from diverse environments, including soil, food, paint, textiles, and plant pathogenic fungi, and is known to cause allergies and diseases in plants and animals (Bensch et al., 2012). It is the most common fungal component isolated from the air (French, 1989, Flannigan and Wotton, 2001). The small conidia of *Cladosporium* are typically formed in branched chains, facilitating their dispersion through the air. The *Cladosporium* species complex includes three major groups: *C. herbarum, C. cladosporioides*, and *C.* sp*haerospermum* (Bensch et al., 2012). The *C.* sp*haerospermum* complex is characterized by globose or subglobose, pigmented, almost smooth to verrucose terminal conidia and 0–3-septate, smooth, or verruculose ramoconidia (Ellis and Yates, 1971; Zalar et al., 2007). The *C. cladosporioides* complex exhibits significant variability, with conidia ranging from smooth or nearly so to irregularly verrucose, vereuuculose, or rough-walled. In contrast, all species within the *C. herbarum* complex have ornamented conidia with features ranging from minutely verruculose to verrucose, echinulate, or spiny (Torres-Cortés et al., 2015). In this study, we introduce one new species and two new host records within the *C. cladosporioides* species complex from Thailand. Phylogenetic analyses of *Cladosporium* are based on combined ITS, *tef1-α*, and *act* sequence data of selected *Cladosporiaceae* isolates. Related sequences were obtained from GenBank. There are 114 taxa included in the analyses, which comprise 2,745 characters including gaps. The tree is rooted in *Cladosporium herbarum*. The best-scoring RAxML tree with a final likelihood value of − 22,003.238457 is presented. The matrix had 1,285 distinct alignment patterns, with 60.03% of undetermined characters or gaps. Estimated base frequencies were as follows: A = 0.233071, C = 0.288405, G = 0.248984, T = 0.229540; substitution rates AC = 1.517403, AG = 3.134825, AT = 1.668455, CG = 1.130508, CT = 4.683471, GT = 1.000000; gamma distribution shape parameter *α* = 0.426819. Maximum likelihood bootstrap support values ≥ 60% (ML) and Bayesian posterior probabilities (PP) ≥ 0.90 are given in the nodes. The scale bar indicates 0.2 changes. The isolates obtained in this study are in red, and ex-types taxa are in black bold (**Supplementary Figure 5**). In this analysis, *Cladosporium dracaenae* isolates from our collections clustered with *Cladosporium dracaenicola* and *Cladosporium tenuissimum*. To confirm the results from phylogenetic analyses, the PHI test was done. Results of the pairwise homoplasy index (PHI) test of *Cladosporium dracaenae*, *Cladosporium dracaenicola*, and closely related species were determined using both LogDet transformation and split decomposition. PHI test results (Φw) < 0.05 indicate significant recombination within the dataset. The new taxon is in red bold type. P=1 (**Supplementary Figure 6**). 328

***Cladosporium dracaenae*** Chaiwan & Jayaward, sp. **nov.** (**Supplementary Figure 7**)

Index Fungorum number: IF 559789 Facesoffungi number: FoF10748

Etymology: Referring to the host genus on which the fungus was collected, *Dracaena* (*Asparagaceae*).

Holotype: MFLU 22–0185

*Saprobic* on dead leaves of *Dracaena* sp. Sexual morph: undetermined. Asexual morph: on PDA. *Mycelium* 2–4 μm wide ( $\bar{\text{x}}$ = 3.2 μm; n = 30), hyphae branched, septate, often appearing somewhat darkened and not constrictions, irregular, hyaline to pale brown or pale olivaceous-brown, smooth, minutely verruculose, thin walls. *Conidiophores* 20–70 × 2–3 μm ( = 45 × 2.5 μm, n = 30), macronematous, arising laterally or terminally from hyphae, erect, straight to slightly flexuous, filiform to narrowly cylindrical 0–3-septate, septa often appearing darkened, sometimes pluriseptate with septa in short succession, especially toward the apex, septa not constricted, pale olivaceous-brown, smooth to minutely verruculose, thin walls or almost so, sometimes forming ramoconidia and fragments., unbranched or branched, brown occasionally slightly geniculate, non-nodulose. *Conidiogenous cells* undifferentiated or conidiophores reduced to conidiogenous cells, cylindrical, 4–18 μm long, usually neither geniculate nor nodulose, with a single or up to three protuberant, subdenticulate, thickened, and darkened. *Conidiogenous scars* thickened and *conspicuous*, protuberant. *Ramoconidia* rarely formed. A branch of a conidiophore at the base of branches having scar, having a truncate or slightly convex, to detached conidiogenous cells or short, fertile, terminal branches, and reclassified branched conidia. *Conidia* 12.5–16.5 × 2.5–3.0 μm ( $\bar{\text{x}}$ = 20 × 2.5 μm; n = 30), catenate, in branched chains, conidial chains branching in all directions, terminal chains with up to nine conidia, small terminal conidia globose or subglobose, hyaline to dark brown, narrower at both ends, straight, guttulate.

***Culture characteristics*:** Colonies on PDA reaching 20–40 mm diam after 2 weeks, olivaceous- gray brown to black, reverse olivaceous- gray to leaden- gray or olivaceous-black, velvety, powdery to felty-woolly, regular, glabrous or feathery, aerial mycelium absent or sparse, growth flat with a somewhat elevated colony center, without prominent exudates, sporulation profuse.

***Material examined***: Thailand, Chiang Rai Province, dead leaves of *Dracaena* sp. (*Asparagaceae*), 17 November 2017, Napalai Chaiwan, NCCR2, (MFLU 22–0185, holotype), ex-type living culture MFLUCC 18–0919.

***GenBank accession numbers***: NCCR2; ITS: OM908927, *tef1-α*: OP099913

***Notes:*** In the phylogenetic analysis, our isolates form a monophyletic clade that is a sister clade to *Cladosporium tenuissimum* (**Supplementary Figure 5**). Morphologically, this collection is similar to *Cladosporium tenuissimum* (in *Cladosporium cladosporioides* complex) (Bensch et al., 2010). Base pair comparison of ITS with *C. tenuissimum* reveals 7 out of 719 (0.97%) nucleotide differences, that in *act* (with *C. tenuissimum* isolate XCWN2) shows 90 out of 515 (17.47%) differences, and that in *tef1-α* (with *Cladosporium* sp. isolate CW_T3L3C12.1) shows 8 out of 1230 (0.65%) differences, indicating that this is a distinct taxon (Jeewon and Hyde, 2016; Chethana et al., 2021; Jayawardena et al., 2021). Consequently, we identify this new species as *C. dracaenae*, reported from *Dracaena* spp. in Thailand. 367

***Cladosporium dracaenicola*** Chaiwan & Jayaward, sp. **nov**. (**Supplementary Figure 8**)

Index Fungorum number: IF559789 Facesoffungi number: FoF10748

Etymology: Referring to the host genus on which the fungus was collected, *Dracaena* (*Asparagaceae*).

Holotype: MFLU 22-0186

*Saprobic* on dead leaves of *Dracaena* sp. Sexual morph: undetermined. Asexual morph: on PDA. *Mycelium* 2–4 μm wide ( $\bar{\text{x}}$ = 3.2 μm; n = 30), hyphae branched, septate, often appearing somewhat darkened and not constrictions, irregular, hyaline to pale brown or pale olivaceous-brown, smooth, minutely verruculose, thin walls. *Conidiophores* 50–80 × 2–3 μm ( $\bar{\text{x}}$ = 65 × 2.5 μm; n = 30), macronematous, arising laterally or terminally from hyphae, erect, straight to slightly flexuous, filiform to narrowly cylindrical 0–3-septate, septa often appearing darkened, sometimes pluriseptate with septa in short succession, especially toward the apex, septa not constricted, pale olivaceous-brown, smooth to minutely verruculose, thin walls or almost so, sometimes forming ramoconidia and fragments., unbranched or branched, brown occasionally slightly geniculate, non-nodulose. *Conidiogenous cells* undifferentiated or conidiophores reduced to conidiogenous cells, cylindrical, 4–18 μm long, usually neither geniculate nor nodulose, with a single or up to three protuberant, subdenticulate, thickened, and darkened. *Conidiogenous scars* thickened and conspicuous, protuberant. *Ramoconidia* rarely formed. *Ramoconidia* 6–12 × 2–3 μm ( $\bar{\text{x}}$ = 8.5 × 2.5 μm; n = 30), a branch of a conidiophore at the base of branches having scar, having a truncate or slightly convex, to detached conidiogenous cells or short, fertile, terminal branches, and reclassified branched conidia. *Conidia* 5–6 × 2.5–3 μm ( $\bar{\text{x}}$ = 5.5 × 2.5 μm; n = 30), catenate, in branched chains, conidial chains branching in all directions, terminal chains with up to nine conidia, small terminal conidia globose or subglobose, hyaline to dark brown, narrower at both ends, straight, guttulate.

***Culture characteristics:*** Colonies on PDA reaching 20–40 mm diam after 2 weeks, olivaceous, gray olivaceous or olivaceous-gray, reverse olivaceous- gray to leaden- gray or olivaceous-black, velvety, powdery to felty-wooly, margins white, regular, glabrous or feathery, aerial mycelium absent or sparse, growth flat with a somewhat elevated colony center, without prominent exudates, sporulation profuse.

***Material examined:*** Thailand, Songkhla Province, Hat Yai District, dead leaves of *Dracaena* sp. (*Asparagaceae*), 5 September 2018, Napalai Chaiwan, TNC6, (MFLU 22–0186, holotype), ex-type living culture MFLUCC 18–0915.

***GenBank accession numbers:*** TNC6; ITS: OM908928.

***Notes:*** In the phylogenetic analysis, our isolates formed a monophyletic clade and are a sister clade to *Cladosporium tenuissimum* (**Supplementary Figure 5**). Morphologically, this collection resembles *Cladosporium cladosporioides* (within the *Cladosporium cladosporioides* complex) (Bensch et al., 2010). Comparison of ITS nucleotides with *C. halotolerans* (CBS127371) reveals 8 out of 594 (1.3%) nucleotide differences, whereas comparison with *C. dracaenae* (MFLU 22-0185) shows 18 out of 594 (3%) nucleotide differences, indicating that they are distinct taxa. Our species showed conidiophores (65 × 2.5 μm) smaller than *Cladosporium tenuissimum* up to (310 × 460 µm). Also, *Ramoconidia* (8.5 × 2.5) our strain smaller than *Cladosporium tenuissimum* (22 × 41 µm). Consequently, we identify the new species as *C. dracaenicola*, which has been reported from *Dracaena* sp. in Thailand.

***Mycosphaerellales*** (Nannf.) P.F. Cannon.

Mycosphaerellales was introduced based on phylogenetic analyses by Schoch et al. (2006), Hyde et al. (2024) recognized 11 families within Mycosphaerellales (*Aeminiaceae, Cystocoleaceae, Dissoconiaceae, Extremaceae, Mycosphaerellaceae, Neodevriesiaceae, Phaeothecoidiellaceae, Phillipsiellaceae, Schizothyriaceae, Teratosphaeriaceae*, and *Xenodevriesiaceae*) (Norimova et al., 2024; Piątek et al., 2024; Hyde et al., 2024). Mostly plant-associated fungi, with a significant number being plant pathogens, endophytes, or saprobes (Norimova et al., 2024; Piątek et al., 2024).

***Mycosphaerellaceae*** Lindau

Wijayawardene et al. (2022) recognized 119 genera within *Mycosphaerellaceae*. Hongsanan et al. (2020) listed 106 genera as doubtful within *Mycosphaerellaceae*, based on the work of Videira et al. (2017).

***Zasmidium*** Fr.

*Zasmidium* is a genus within *Mycosphaerellaceae*, established by Fries in (1849). *Zasmidium cellare* is the type species of this genus (Zhao et al., 2016; Arzanlou et al., 2007). Species of *Zasmidium* have been reported from various plant hosts (Shivas et al., 2010; Crous et al., 2014; Quaedvlieg et al., 2014). In this study, we report a new record of *Zasmidium citrigriseum* from Thailand. For *Zasmidium*, the phylogram was generated from RAxML analysis based on combined LSU, ITS, and *act* sequence data of selected *Zasmidium* isolates. Related sequences were obtained from GenBank. There are 27 taxa included in the analyses, which comprise 2,044 characters including gaps. The tree is rooted in *Pseudozasmidium eucalypti* (CBS121101). The best-scoring RAxML tree with a final likelihood value of − 6,614.766180 is presented. The matrix had 454 distinct alignment patterns, with 33.32% of undetermined characters or gaps. Estimated base frequencies were as follows: A = 0.232177, C = 0.263299, G = 0.287808, T = 0.216717; substitution rates AC = 1.575049, AG = 2.045497, AT = 1.643817, CG = 1.149239, CT = 5.459635, GT = 1.000000; gamma distribution shape parameter *α* = 0.144082. Maximum likelihood bootstrap support values ≥ 60% (ML) and Bayesian posterior probabilities (PP) ≥ 0.90 are given in the nodes. The scale bar indicates 0.04 changes. The isolates obtained in this study are in red, and ex-types taxa are in black bold (**Supplementary Figure 9**). In this analysis, our collection clustered with 83% ML and 0.93 PP of *Zasmidium citrigriseum*.

***Zasmidium citrigriseum*** (F.E. Fisher) U. Braun & Crous [as “citri-griseum”], IMA Fungus 5(2): 337–442 (2014) (**Supplementary Figure 10**)

Index Fungorum number: IF 622149

Saprobic on dead leaves of *Dracaena* sp. Sexual morph: see Crous et al. (2014). Asexual morph: *Mycelium* external, smooth, septate, branched, with wide hyphae. Conidiophores micronematous, arising from superficial mycelium, solitary, erect, straight to slightly curved, branched laterally or unbranched, medium to dark brown, slightly wider at the base. *Conidiogenous cells* terminal, cylindrical to subcylindrical, tapering to a flattened apical region, smooth to finely verruculose, medium brown, measuring 20 × 60 µm; scars thickened, somewhat darkened, 2–2.5 µm wide. *Conidia* broadly fusiform to obovoid, 0–1-septate, pale olivaceous, paler toward the apex; terminal conidia ovoid, 0-septate, pale olivaceous to hyaline, paler toward the apex, with a truncate base extending up to 5 µm wide on each side, tapering toward the polar ends.

*Culture characteristics:* Colonies on PDA reaching 2–4 cm diameter after 2 weeks. Young colonies are olivaceous-brown, darkening with age. The reverse is olivaceous-brown to leaden- gray or olivaceous-black. Texture ranges from velvety and powdery to felty-wooly.

*Material examined:* Thailand, Nakhon Si Thammarat Province, Cha-uat District, on dead leaves of *Dracaena* sp., 19 December 2018, Napalai Chaiwan, specimen NSW1 (MFLU 22-0189); living culture MFLUCC 18–0903.

*Known distribution:* Vietnam, Thailand, USA. Also reported from *Acacia confusa*, *Albizia chinensis*, *Magnolia candollii*, *Podocarpus macrophyllus* (China) (Dou et al., 2017; de Silva et al., 2019), *Mangifera indica* (Thailand) (Trakunyingcharoen et al., 2014), and dead leaves of *Dracaena fragrans* (this study).

*GenBank accession numbers:* ITS: OM919719, LSU: OM919720, *act*: OP099914

***Notes:*** *Zasmidium citrigriseum* has been reported from various hosts in different countries, including *Musa* in America, *Acacia* in Thailand, *Eucalyptus* in Vietnam, and *Aeglopsis*, *Citrus*, *Fortunella*, *Murraya*, and *Poncirus* in North and South America (Crous et al., 2019; Pretorius et al., 2003). In our study, we identified the same fungus in Thailand on *Dracaena* sp. A BLASTn search of GenBank showed that the LSU sequence had 99.42% similarity with *Zasmidium anthuriicola* (no nucleotide differences in the aligned region), confirming our strain as a new host record of *Zasmidium citrigriseum* from *Dracaena* in Thailand.

Pleosporales Luttr. ex M.E. Barr

*Pleosporales* is one of the most species-rich orders within the class *Dothideomycetes*, encompassing species that inhabit a wide range of ecosystems, including terrestrial, freshwater, and marine environments (Phookamsak et al., 2014, 2017; Bakhshi et al., 2019; Jones et al., 2019a, b; Luo et al., 2019; Tennakoon et al., 2019). Hongsanan et al. (2020) and Wijayawardene et al. (2022) recognized 91 genera within *Pleosporales*. The family *Paralophiostomataceae* was introduced into this order by Hongsanan et al. (2020).

***Didymellaceae*** Gruyter, Aveskamp & Verkley

Hyde et al. (2024) recognized 44 genera within *Didymellaceae*. Taxa in this family are known to cause plant diseases, including leaf and stem blight, which can lead to plant death (Wanasinghe et al., 2018; Phukhamsakda et al., 2020, Wijayawardene et al., 2022).

***Longididymella*** L.W. Hou, L. Cai & Crou

The type species of *Longididymella*, originally described as *Phoma clematidina* on *Clematis ligusticifolia* (causing necrotic leaf spot), was characterized by both its sexual and asexual morphs (Woudenberg et al., 2009). The new combination *Longididymella clematidis* was synonymized by Hou et al. (2020) from *Anthodidymella clematidis* and proposed as *Didymella clematidis* (CBS 123705) (Phukhamsakda et al., 2020). *Longididymella* species are saprobic or necrotic and commonly occur on flowering and herbaceous plants. Previously, this species was introduced from *Clematis* plants in the USA (Woudenberg et al., 2009; Phukhamsakda et al., 2020). In this study, we isolated the asexual morph of *Longididymella clematidis* from *Dracaena* sp. in Thailand. Phylogenetic analysis of *Longididymella* was generated from RAxML analysis based on combined LSU sequence data of selected *Didymellaceae* isolates. Related sequences were obtained from GenBank. There are 28 taxa are included in the analyses, which comprise 1,354 characters including gaps. The tree is rooted to *Calophoma complanate* (CBS 268.92). The best- scoring RAxML tree with a final likelihood value of − 1,470.814319 is presented. The matrix had 42 distinct alignment patterns, with 4.19% of undetermined characters or gaps. Estimated base frequencies were as follows: A = 0.254580, C = 0.209195, G = 0.302369, T = 0.233856; substitution rates AC = 2,406.556015, AG = 21,439.684114, AT = 2,181.215287, CG = 2,016.393030, CT = 45,321.905016, GT = 1.000000; gamma distribution shape parameter *α* = 0.020000. Maximum likelihood bootstrap support values ≥ 60% (ML) and Bayesian posterior probabilities (PP) ≥ 0.90 are given in the nodes. The scale bar indicates 0.002 changes. The isolates obtained in this study are in red and ex-types taxa are in black bold (**Supplementary Figure 11**). In this analysis, our collections clustered with *Longididymella clematidis* CBS123705.

***Longididymella clematidis*** (Woudenb., Spiers & Gruyter) L.W. Hou, L. Cai & Crous, in Hou, Groenewald, Pfenning, Yarden, Crous & Cai, Stud. Mycol. 96: 339 (2020) (**Supplementary Figure 12**)

Index Fungorum number: IF833503

Basionym: *Didymella clematidis* Woudenb., Spiers & Gruyter in Woudenberg et al., Persoonia 22:60 (2009)

*Saprobic* on dead leaves of *Dracaena* sp. Sexual morph: Undetermined. Asexual morph: *Conidiomata* 50–400 × 80–350 μm ( $\bar{\text{x}}$ = 225 × 215 μm; n = 5), pycnidial, solitary, sometimes aggregated, uniloculate, immersed under epidermal layer, subglobose to depressed, coriaceous thin-walled, brown to dark brown, with ostiolate. *Ostioles* not observe. *Conidiomatal wall* 10–30 μm wide, of two to five layers, each cell-layer 10 μm wide, light- brown to brown cells of *textura globulosa*, heavily pigmented in the outer layers, lined with a hyaline innermost layer bearing conidiogenous cells. *Conidiophores* reduced to conidiogenous cells. *Conidiogenous cells* 2.5–4.5 × 1.5–3.5 μm ( $\bar{\text{x}}$ = 3.5 × 2.5 μm; n = 20), phialidic, determinate, discrete, ampulliform, cylindrical to sub-cylindrical, smooth-walled, hyaline, arising from the inner layer of conidioma. *Conidia* 2–3 × 4–8 μm ( $\bar{\text{x}}$ = 2.5 × 6 μm; n = 50), oblong or oval, slightly curved toward the ends, rounded ends, with 1(–2) guttules in each cell, hyaline, aseptate, smooth-walled.

***Culture characteristics:*** Colonies on PDA reaching 2–4 cm diam after 7 days dark-brown color, reverse dark-brown to black, margins white, regular aerial mycelium, growth flat with a somewhat elevated colony Center, without prominent exudates, sporulation profuse.

***Material examined:*** Thailand, Tak Province, Umphang District, on dead leaves of *Dracaena* sp., 21 August 2019, Napalai Chaiwan, Umpangsoil2 (MFLU 22-0187), living culture MFLUCC 22-0099. ***Host and Distribution*:** *Clematis ligusticifolia* (Woudenberg et al., 2009), *Dracaena* sp. (This study). USA (Woudenberg et al., 2009), Thailand (This study).

***GenBank accession numbers:*** LSU: ON000548.

***Notes:*** *Longididymella clematidis* (MFLU 22-0187) has been previously reported causing leaf spots on *Clematis ligusticifolia* (Woudenberg et al., 2009). Our strain represents a new host record (Phukhamsakda et al., 2020; Woudenberg et al., 2009). A BLASTn search of GenBank revealed that the LSU sequence of our strain has 99.54% similarity (5/908 = 0.55% nucleotide differences) to *Longididymella clematidis* (CBS123705). Our species is characterized by pseudothecial, globose, subglobose to pyriform ascomata, cylindrical asci with club-shaped bases and hyaline, septate, ovate to obpyriform ascospores. Conidiomata pycnidial, solitary, sometimes aggregated, uniloculate, immersed under the epidermal layer, brown to dark brown. Based on these, we identify this strain as a new host record for *Longididymella clematidis*.

***Pleosporaceae*** Nitschke

Hyde et al. (2024) accepted 23 genera in *Pleosporaceae* comprising *Allonecte, Alternaria, Bipolaris, Clathrospora, Comoclathris, Curvularia, Decorospora, Diademosa, Dichotomophthora, Exserohilum, Extrawettsteinina, Gibbago, Johnalcornia, Paradendryphiella, Platysporoides, Pleoseptum, Porocercospora, Prathoda, Pseudoyuconia, Pyrenophora* (= Marielliottia), *Tamaricicola*, and *Typhicola*. *Pleosporaceae* taxa occur as saprobes and plant pathogens.

***Bipolaris*** Shoemaker

*Bipolaris* species are plant pathogens with a global distribution (Hongsanan et al., 2020; Ferdinandez et al., 2022, 2024). They are commonly reported on a variety of plant diseases, including leaf spots, leaf blights, root rots, and other symptoms in field crops (Manamgoda et al., 2014). *Bipolaris* species are characterized by dematiaceous hyphomycetous fungi that produce pale- brown to dark- brown asexual conidia and conidiophores. In this study, we present a new record of Bipolaris from a different host in Thailand. Phylogenetic analyses of *Bipolaris* were done using RAxML analysis based on combined ITS and *tef1-α* sequence data of selected *Pleosporaceae* isolates. Related sequences were obtained from GenBank. There are 23 taxa included in the analyses, which comprise 1,537 characters including gaps. The tree is rooted to *Curvularia lunata*. The best-scoring RAxML tree with a final likelihood value of − 2,674.255709 is presented. The matrix had 139 distinct alignment patterns, with 15.20% of undetermined characters or gaps. Estimated base frequencies were as follows: A = 0.218186, C = 0.246306, G = 0.288802, T = 0.246706; substitution rates AC = 0.763615, AG = 5.761060, AT = 2.722841, CG = 0.695515, CT = 2.038141, GT = 1.000000; gamma distribution shape parameter *α* = 0.020000. Maximum likelihood bootstrap support values ≥60% (ML) and Bayesian posterior probabilities (PP) ≥ 0.90 are given in the nodes. The scale bar indicates 0.02 changes. The isolates obtained in this study are in red and ex-type taxa are in black bold (**Supplementary Figure 13**). In this analysis, our collections clustered with *Bipolaris coffeana* (BRIP14845).

***Bipolaris coffeana*** Sivan., Trans. Br. mycol. Soc. 84(3): 404 (1985) (**Supplementary Figure 14**)

Index Fungorum number: IF105089

*Saprobic* on dead leaves of *Dracaena* sp. Sexual morph: Undetermined. Asexual morph: *Vegetative hyphae* septate, subhyaline to brown, branched, smooth, 3–4 μm in width. Colonies on PDA reaching approximately 4–6 cm in diameter after 7 days at 25°C, surface funiculose, margin fimbriate, olivaceous black to olivaceous grey, velvety with sparse aerial mycelium. *Conidiophores* erect, often branched, in most cases uniformly brown, sometimes pale brown at apex, arising singly, septate, flexuous, in most cases geniculate toward the apex, up to 210 μm long, 3–4 μm wide, generally wide in basal parts, tapering in median and upper parts, straight or flexuous, geniculate in apex, frequently unbranched, cells walls thicker than those of the vegetative hyphae, pale brown to brown, basal cells sometimes swollen. *Conidiogenous cells* mostly integrated, terminal, or intercalary with sympodial proliferation, smooth, brown, mono- or polytretic. *Conidia* with bipolar germination, four-celled, smooth- walled or slightly verruculose, asymmetrically swollen and curved at the third cell from base, rarely symmetric swelling and straight, pale to dark brown, end cells paler and thin-walled than central cells, ellipsoidal to clavate to obovoid, asymmetrical with paler end cells, usually curved at the third cell from the base, (80–110 × 10–18 μm, 3-distoseptate, hila slightly protuberant, thickened and darkened. *Chlamydospores* with verruculose nodes, terminal or intercalary, proliferating sympodially, with circular and thickened scars, brown, cylindrical to swollen.

***Culture characteristics:*** Colonies growing on PDA 4–6 cm diam after 8 d days of incubation at 25°C, circular with filiform margin, dark green to greenish-black, aerial mycelium sparse to moderate, floccose with age; reverse greyish green to brownish black.

***Material examined:*** Thailand, Chiang Rai Province, Mea Lao district, on dead leaves of *Dracaena* sp., 17 November 2017, Napalai Chaiwan, DNC1.1 (living culture MFLUCC 17–2600).

***Host and distribution*:** *Bouteloua gracilis*, *Coffea arabica* (Kenya) (Tan et al., 2014), *Cynodon dactylon* (New Zealand) (Manamgoda et al., 2012), *Digitaria* sp. (*Poaceae*) (USDA fungal host data base) —*Dracaena fragrans* Thailand, USA (Jeon et al., 2015, This study).

***GenBank accession numbers:*** ITS: OP090572, *tef1-α*: OP099915.

***Notes:*** *Bipolaris coffeana* has been reported from *Coffea arabica* in Kenya (Tan et al., 2014) and also from *Bouteloua gracilis* and *Cynodon dactylon*. The combined phylogenetic analysis of the *Bipolaris generic* complex in this study shows that our strain has high bootstrap support with *Bipolaris coffeana*. This section includes descriptions and illustrations of the new host record based on molecular and morphological data (Ferdinandez et al., 2022). This species is characterized by fusiform shaped conidia, central cells not much darker but broader than the distal ones. This is the first report of *Bipolaris coffeana* isolated from *Dracaena fragrans* in Thailand.

***Curvularia*** Boedijn

*Curvularia* species are significant pathogens reported globally. They have been identified as plant pathogenic fungi and have also been associated with animal and human diseases (Kusai et al., 2016). Previously, the classification of *Curvularia* species was unclear due to a lack of molecular data, and the genus exhibits overlapping morphological characteristics with *Bipolaris* (Manamgoda et al., 2012). In this study, we present a new host record for *Curvularia lunata* from Thailand. Phylogenetic analyses of *Curvularia* were based on ITS sequence data of selected *Pleosporaceae* isolates. Related sequences were obtained from GenBank. There are 92 taxa included in the analyses, which comprise 694 characters including gaps. The tree is rooted with *Cochliobolus nisikadoi*. The best-scoring RAxML tree with a final likelihood value of −4,650.452400 is presented. The matrix had 358 distinct alignment patterns, with 26.96% of undetermined characters or gaps. Estimated base frequencies were as follows: A = 0.236630, C = 0.245701, G = 0.229962, T = 0.287707; substitution rates AC = 1.587196, AG = 2.075234, AT = 1.262201, CG = 1.663658, CT = 3.666320, GT = 1.000000; gamma distribution shape parameter *α* = 0.245992. Maximum likelihood bootstrap support values ≥ 60% (ML) and Bayesian posterior probabilities (PP) ≥ 0.90 are given in the nodes. The scale bar indicates 0.03 changes. The isolates obtained in this study are in red and ex-type taxa are in black bold (**Supplementary Figure 15**). In this analysis, our collections clustered with 88% ML and 1 PP with *Curvularia lunata* (CBS730.96).

***Curvularia lunata*** (Wakker) Boedijn, Bull. Jard. bot. Buitenz, 3 Sér. 13(1): 127 (1933) (**Supplementary Figure 16**)

Index Fungorum number: IF269889

*Saprobic* on dead leaves of *Dracaena* sp. Sexual morph: Undetermined. Asexual morph: *Conidiophores* 155–160 × 60–70 μm ( $\bar{\text{x}}$ = 157 × 65 μm; n = 10), arising occasionally singly or usually in groups, branched, septate, straight, geniculate at upper part, brown to pale reddish, lighter toward apex. *Conidiogenous nodes* dark brown, smooth. *Conidia* 159–165 × 70–75 μm ( $\bar{\text{x}}$ = 162 × 72 μm; n = 30), smooth-walled, straight or curved, subcylindrical to fusoid, sometimes narrowly clavate, tapering toward rounded apex, basal cell obconic, pale brown to mid reddish brown, 3–4- distoseptate. *Hilum* inconspicuous, germination bipolar.

***Culture characteristics:*** Colonies growing on PDA 4–6 cm diam after 8 d days of incubation at 25°C, circular with filiform margin, white or cream, aerial mycelium sparse to moderate, floccose with age; reverse brownish to brown.

***Material examined:*** Thailand, Kanchanaburi Province, on dead leaves of *Dracaena* sp., 27 June 2019, Napalai Chaiwan, KAN20 (living culture MFLUCC 22-0076).

***Host and Distribution*:** *Adhatoda* sp., A*gave sisalana, Agrostis palustris, Allium cepa, Allium sativum, Alnus rubra, Aloe vera, Amaranthus* sp*inosus, Anacardium occidentale, Ananas comosus, Andropogon sorghum* var. *sudanensis, Anthephora hermaphrodita, Arachis hypogaea, Areca catechu, Artocarpus integra, Axonopus affinis, Bambusa vulgaris, Boehmeria nivea, Brachiaria mutica, Brassica rapa* subsp. *pekinensis, Cajanus cajan, Capsicum annuum* var. *annuum, Capsicum frutescens, Citrus reticulata, Dactyloctenium aegyptium, Dendrocalamus strictus, Digitaria ischaemum, Echinochloa colona, Eleusine indica, Eucalyptus globulus, Fragaria ananassa, Gladiolus hortulanus, Halophila ovalis, Helianthus annuus, Impatiens sultanii, Ipomoea batatas, Legenaria vulgaris, Linum usitatissimum, Lolium multiflorum, Mangifera indica, Rosa* sp., *Zea mays* (Farr and Rossman, 2025) —of *Dracaena* sp., (This study). Australia, Brazil, California, China, Cuba, Hong Kong, India, Indonesia, Malaysia, Mauritius, Myanmar, Thailand, USA, and Virginia (Farr and Rossman, 2024).

***GenBank accession numbers:*** ITS: OP090556.

***Notes:*** *Curvularia lunata* is the type species of the genus (Boedijn, 1933). It was originally reported from *Saccharum officinarum* (sugar cane) in Java and is also known to cause leaf spots on *Clerodendrum indicum*, an important medicinal plant (Mukherjee et al., 2013), as well as on *Zea mays*, leading to significant yield losses (Li et al., 2006; Manamgoda et al., 2012). There have been reports of *Curvularia lunata* from unknown grasses in Thailand (Manamgoda et al., 2014; Tan et al., 2016). In this study, we isolated *Curvularia lunata* (KAN20) from *Dracaena* sp., a plant substrate in Thailand in 2018. This culture is undergoing sporulation. Many strains of *C. lunata* are misidentified in GenBank (Cai et al., 2011), and accurate identification of the type sequences for *Curvularia* species has been recently reported by Bhunjun et al. (2020).

***Torulaceae*** Nitschke

In 1794, Persoon introduced the genus *Torula* with *T. herbarum* as the type species (Persoon, 1794). Members of *Torulaceae* are commonly found as saprobes on various terrestrial plants and submerged decaying wood in rivers or streams (Su et al., 2018). *Torulaceae* species reported only in their asexual morphs, characterized by micro- or macronematous conidiophores that are erect from the host or aerial, and connected to doliform or ellipsoid, brown, smooth to verruculose conidia (Yang et al., 2022). There are seven genera associated with this family (Wijayawardene et al., 2022).

***Torula*** Pers.

The type species of this genus is *Torula herbarum* was designated from a neotype (CPC 24114) (Crous et al., 2015). The members of *Torula* are mainly characterized by terminal or lateral, monoblastic, or polyblastic conidiogenous cells which have a basally thickened and heavily melanized wall, with the apex thin-walled and frequently collapsing and becoming coronate (Crane and Miller, 2016). *Torula* has over 500 epithets in Index Fungorum (2025). In the present study, we introduce a new species of *Torula* from dead leaves of *Dracaena* sp. from Thailand. Phylogenetic analyses of *Torulaceae* was based on combined ITS, LSU, SSU, and *rpb2* sequence data of selected *Torulaceae* isolates. Related sequences were obtained from GenBank. There are 44 taxa included in the analyses, which comprise 864 characters including gaps. The tree is rooted to *Dendryphion europaeum* (CPC23231). The best-scoring RAxML tree with a final likelihood value of − 7,716.685771 is presented. The matrix had 517 distinct alignment patterns, with 31.01% of undetermined characters or gaps. Estimated base frequencies were as follows; A = 0.249849, C = 0.244700, G = 0.278439, T = 0.227012; substitution rates AC = 2.770901, AG = 3.096398, AT = 2.134703, CG = 1.838546, CT = 7.075441, GT = 1.000000; gamma distribution shape parameter *α* = 0.312119. Maximum likelihood bootstrap support values ≥ 60% (ML) and Bayesian posterior probabilities (PP) ≥ 0.90 are given in the nodes. The scale bar indicates 0.03 changes. The isolates obtained in this study are in red and ex-type taxa are in black bold (**Supplementary Figure 17**).

In this analysis, our collections clustered with 100% ML and 1 PP of *T. breviconidiophora* KUMCC 18–0130 and 70% ML and 1.00 PP of *T. chromolaenae* (KUMCC 16-0036). This analysis was further confirmed with PHI test. Results of the pairwise homoplasy index (PHI) test of *Torula dracaenae* and closely related species using both LogDet transformation and splits decomposition. PHI test results (Φw) < 0.05 indicate significant recombination within the dataset. The new taxon is in red bold type. P=0.1959 (**Supplementary Figure 18**). Based on these, we introduce our collection as a new species *Dracaena* sp.

***Torula dracaenae*** Chaiwan & Jayaward, sp. nov. (**Supplementary Figure 19**).

Index Fungorum number: IF559437 Facesoffungi number: FoF10752

Etymology: Referring to the host genus from which the fungus was collected: *Dracaena* (*Asparagaceae*).

Holotype: MFLU 22-0135

*Saprobic* on dead leaves of *Dracaena* sp. Sexual morph: undetermined. Asexual morph: Colonies effuse on host, black, powdery. *Mycelium* partly immersed, composed of septate, branched, smooth, pale- brown hyphae. *Conidiophores* (2.8–)3–4.3 × 2.5–3 μm ( $\bar{\text{x}}$ = 3.8 × 2.8 μm; n = 10), macronematous, mononematous, solitary, erect, pale brown, verruculose, thick-walled, consisting of one to two cells or reduced to conidiogenous cells, subcylindrical to subglobose, arising from prostrate hypha. *Conidiogenous cells* (3–)3.2–3.5 × 3.8–4.6 μm ( $\bar{\text{x}}$ = 3.4 × 4.1 μm; n = 10), polyblastic, terminal, dark brown to black, smooth to minutely verruculose, thick-walled, doliiform to ellipsoid. *Conidia* 10–15 × 3.0–4.5 μm ( $\bar{\text{x}}$ = 13 × 3 μm; n = 20) solitary to catenate, acrogenous, simple, phragmosporous, dark brown, with apical cell pale brown, minutely verruculose, 3–10-septate, rounded at both ends, composed of subglobose cells, slightly constricted at some septa, chiefly subcylindrical, rough-walled.

***Material examined:*** Thailand, Kanchanaburi Province, on dead stems of *Dracaena* sp., 20 March 2017, Napalai Chaiwan, KAN2, (MFLU 22-0135, holotype).

***GenBank accession numbers:*** LSU: OM911934; ITS: OM911931.

***Notes:*** Phylogenetic analysis of our collection grouped it with *Torula chromolaenae* and *T. mackenziei*, showing a close relationship to *T. breviconidiophora* (**Supplementary Figure 17**). Our strain clustered with *T. breviconidiophora* with strong bootstrap support (100% ML, 1 PP, **Supplementary Figure 17**). Morphologically, our collection differs from *T. mackenziei* and *T. breviconidiophora* but is similar to *T. chromolaenae*. Its conidiophores (3.8 × 2.8 μm) and conidiogenous cells (3.4 × 4.1 μm) are smaller than those of *T. chromolaenae* (5.8 × 4 μm and 4.7 × 5.4 μm, respectively). Additionally, its conidia (13 × 3 μm) are smaller than those of *T. chromolaenae* (14.5 × 4.3 μm). We were unable to obtain a culture as the spores did not germinate. A BLASTn search of GenBank showed that the ITS sequence of our strain has 98.45% similarity to *T. chromolaenae*. The ITS nucleotides reveal 12/729 (1.64%) differences, LSU nucleotides show 1/978 (0.1%) differences, and the SSU nucleotides show 64/1090 (5.87%) differences indicating that they are distinct taxa (Jeewon and Hyde, 2016; Pem et al., 2024). The PHI test for closely related species showed a P-value of 0.2078, indicating no significant evidence of recombination. Based on the phylogenetic support, we introduce our isolate as a new species from *Dracaena* sp. in Thailand.

**Venturiales** Y. Zhang ter, C.L. Schoch & K.D. Hyde

Wijayawardene et al. (2022) recognized three families within the order Venturiales: *Cylindrosympodiaceae, Sympoventuriaceae*, and *Venturiaceae.* Shen et al. (2020) introduced *Cylindrosympodiaceae* with eight genera: *Bellamyces, Fagicola, Fraxinicola, Neofusicladium, Parafusicladium, Fuscohilum, Pinaceicola*, and *Sterila*. However, Wijayawardene et al. (2022) included only *Cylindrosympodium, Pseudoanungitea, Septonema, Sympodiella*, and *Tothia* in this family.

***Sympoventuriaceae*** Y. Zhang ter, C.L. Schoch & K.D. Hyde

Wijayawardene et al. (2022) recognized 17 genera in *Sympoventuriaceae*. Members of *Sympoventuriaceae* are often opportunistic pathogens of vertebrates in natural habitats (Dwivedi, 1959, Barron and Busch, 1962, Hoog et al., 2000) and have also been implicated in human infections (Salkin et al., 1994; Padhye et al., 1994; Singh et al., 2006; Yarita et al., 2007; Ge et al., 2009).

***Ochroconis*** de Hoog & Arx

Wijayawardene et al. (2022) recognized 28 species in the genus *Ochroconis*, with *Ochroconis constricta* serving as the type species. These species are characterized by sympodial conidiogenesis and septate, ellipsoidal conidia that are released rhexolytically. In this study, we report a new record of *Ochroconis musae* from Thailand. Hylogenetic analysis of *Ochroconis* was based on combined LSU, ITS, and *tub* sequence data of selected *Didymellaceae* isolates. Related sequences were obtained from GenBank. There are 28 taxa included in the analyses, which comprise 2,385 characters including gaps. The tree is rooted to *Verruconis calidifluminalis* (CBS125818). The best-scoring RAxML tree with a final likelihood value of − 17,388.241509 is presented. The matrix had 1,158 distinct alignment patterns, with 21.98% of undetermined characters or gaps. Estimated base frequencies were as follows; A = 0.232186, C = 0.241611, G = 0.316968, T = 0.209236; substitution rates AC = 0.942091, AG = 1.876611, AT = 1.006528, CG = 1.020611, CT = 3.796142, GT = 1.000000; gamma distribution shape parameter *α* = 0.257007. Maximum likelihood bootstrap support values ≥ 60% (ML) and Bayesian posterior probabilities (PP) ≥ 0.90 are given in the nodes. The scale bar indicates 0.08 changes. The isolates obtained in this study are in red and ex- type taxa are in black bold (**Supplementary Figure 20**). In this analysis, our collections clustered with *Ochroconis musae* CBS729.95 and (MFLU 17-2598).

***Ochroconis musae*** (G.Y. Sun & Lu Hao) Samerp. & de Hoog, Mycol. Progr. 14(no. 6): 8 (2015) (**Supplementary Figure 21**)

Index Fungorum number: IF808843

*Saprobic* on dead leaves of *Dracaena* sp. Sexual morph: Undetermined. Asexual morph: *Hyphae* effusae, pallide brunneae vel brunneae. *Mycelium* mostly superficiale, immersed, branched septate, pale *olive*, 1.0–1.5 μm wide. *Conidiophores* 0–3-septata, arising directly from aerial hyphae, straight or slightly flexuous, pale olivaceous brown, smooth, denticulate, solitary. *Conidiogenous cells* sympodial, denticulate, denticles thread-like, 0.5×1.0 μm, breaking between conidia and conidiogenous cells. *Conidia* 9–13 × 2–3 μm ( $\bar{\text{x}}$ = 11 × 2.5 μm; n = 20) solitary, subcylindrical, pale brown, smooth, with the distal end obtuse and proximal end slightly tapered, bearing a minute denticle, clearly 1-septate in the middle, guttulate, conspicuously constricted at the septum.

***Culture characteristics:*** On PDA, colonies 25–30 mm in diameter after 2 weeks, flat, moderately expanding, smooth, dry, flat. The center is white or beige, the margin is grayish brown to dark brown, submerged colony margin, reverse as dark brown in the central portion, not producing pigmentation in agar, hyphae sub-hyaline to pale brown, smooth- and thick-walled, sporulated after 3 weeks.

***Material examined:*** Thailand, Tak Province, Umphang District, on dead leaves of *Dracaena* sp., 23 August 2019, Napalai Chaiwan, poppra5.1 (MFLU 22–0188), living culture MFLUCC 22-0105.

***Known distribution*:** *Musa basjoo* (Samerpitak et al., 2015), *Persea americana* (Crous et al., 2019), *Dracaena* sp (Hyde et al., 2020). China (Samerpitak et al., 2015), Thailand (Crous et al., 2019; Hyde et al., 2020, This study).

***GenBank accession numbers:*** ITS: ON159737; LSU: ON159739; *tub*: ON184274.

***Notes:*** *Ochroconis musae* has previously been recorded from *Musa basjoo* (Samerpitak et al., 2015). Our isolate, collected from *Dracaena* sp. in Thailand, grouped with other *Ochroconis musae* strains in a monophyletic clade with 88% ML support in the phylogenetic analysis (**Supplementary Figure 20**). Thus, we identify this collection as a new host record of *O. musae* from *Dracaena* sp. in Thailand.

## 4 Discussion

This study provides morphological descriptions and phylogenetic analyses of 11 fungal taxa isolated from *Dracaena* species associated with limestone outcrops in Thailand. We describe three new species (*Cladosporium dracaenae*, *C. dracaenicola*, and *Torula dracaenae*), identify seven new host records (*Bipolaris coffeana*, *Curvularia lunata*, *Lasiodiplodia bruguierae*, *L. lignicola*, *L. thailandica*, *Longididymella clematidis*, and *Zasmidium citrigriseum*), and report a new collection of *Ochroconis musae*. These findings considerably expand current knowledge on microfungi associated with *Dracaena* in Thailand.

Our collection represents five fungal orders and among these, Botryosphaeriales and Cladosporiales were predominant, aligning with previous research highlighting their common association with woody substrates and their prevalence in tropical and subtropical habitats (Hyde et al., 2005; Marques et al., 2013; Gomdola et al., 2024; Pereira et al., 2024; Slippers et al., 2024). Members of Botryosphaeriaceae, in particular, are frequently recognized as pathogens, endophytes, or saprobes on woody plants and have been recently reported from limestone-rich habitats (Dissanayake et al., 2021; Zhang et al., 2023). For instance, Botryosphaeria guttulata was first described from limestone geology in Guizhou Province, China (Chen et al., 2020), suggesting potential ecological adaptations of Botryosphaeriaceae to extreme environments. It is noteworthy to further explore these ecological roles and their adaptation mechanisms within limestone habitats. In the current study, we introduced two new *Cladosporium* species viz. *C. dracaenae* and *C. dracaenicola*. *Cladosporium* species are ubiquitous fungi commonly associated with biodeterioration processes of limestone substrates, particularly in humid and temperate regions (Nováková, 2009; Trovão et al., 2020). Notably, species such as *Cladosporium cladosporioides* and *C. tenuissimum* have been documented to produce calcium oxalate crystals and acidic metabolites, respectively (Burford et al., 2003; Waqas et al., 2013), facilitating mineral transformations and potentially compromising structural integrity of limestone substrates. Thus, future studies should investigate the potential industrial and ecological impacts of these newly described *Cladosporium* species isolated from limestone environments.

Comparing our findings with earlier records, Thongkantha et al. (2008) documented fungal saprobes and pathogens associated with *Dracaena lourieri* in Thailand, largely relying on phylogenetic analyses from baited and natural substrates, with limited morphological integration. Our investigation aligns with several previous reports while providing clearer taxonomic identification and integrating comprehensive morphological and molecular approaches. For instance, *Ochroconis musae*, previously reported from bananas in China and *Dracaena* in Thailand (Hyde et al., 2020), was also isolated herein, confirming its broader host range. Similarly, although Torula herbarum has been recorded on *Dracaena* (Li et al., 2017), we distinguish and introduce *Torula dracaenae* as a new species. Additionally, we reconfirmed *Curvularia lunata* (previously reported by Peregrine and Ahmed, 1982) and reported *Bipolaris coffeana* as a novel association with *Dracaena*. Despite Braun et al. (2014) reporting *Zasmidium dracaenae* on *Dracaena*, we document *Z. citrigriseum* as a new host record in Thailand. *Lasiodiplodia thailandica*, previously reported from *Mangifera indica* in Thailand, is herein reported along with new host records of *L. bruguierae* and *L. lignicola* from *Dracaena*. *Longididymella clematidis*, earlier known only from *Clematis ligusticifolia* in the USA, represents both a new host and geographical record in this study.

Our results demonstrate significant diversity within the fungal community associated with *Dracaena* species in limestone outcrops in Thailand. Earlier studies often lacked precise identification due to insufficient molecular data. Therefore, future recollections and detailed phylogenetic reassessments are crucial. Biochemical characterization of fungal taxa from limestone habitats would be valuable, given their potential ecological roles and biotechnological applications. Future research should prioritize understanding the ecological contributions and adaptation strategies of fungi colonizing limestone ecosystems.

## Data Availability

The datasets presented in this study can be found in online repositories. The names of the repository/repositories and accession number(s) can be found in the article/**Supplementary Material**.
