# Correction: Morphology and molecular phylogeny of *Dothideomycetes* fungi associated with *Dracaena* plants

**DOI:** 10.3389/fcimb.2025.1686965

**Published:** 2025-09-05

**Authors:** Napalai Chaiwan, Kevin David Hyde, Ruvishika Shehali Jayawardena, Saowaluck Tibpromma, Dhanushka N. Wanasinghe, Ishara Sandeepani Manawasinghe, Dimuthu S. Manamgoda, Itthayakorn Promputtha

**Affiliations:** ^1^ Office of Research Administration, Chiang Mai University, Chiang Mai, Thailand; ^2^ Department of Biology, Faculty of Science, Chiang Mai University, Chiang Mai, Thailand; ^3^ Center of Excellence in Fungal Research, Mae Fah Luang University, Chiang Rai, Thailand; ^4^ Mushroom Research Foundation, School of Science, Chiang Mai, Thailand; ^5^ School of Science, Mae Fah Luang University, Chiang Rai, Thailand; ^6^ Center for Yunnan Plateau Biology Resources Protection and Utilization, College of Biology and Food Engineering, Qujing Normal University, Qujing, Yunnan, China; ^7^ Department of Soil Science, College of Food and Agriculture Sciences, King Saud University, Riyadh, Saudi Arabia; ^8^ Innovative Institute for Plant Health, Zhongkai University of Agriculture and Engineering, Guangzhou, China; ^9^ Department of Botany, University of Sri Jayewardenepura, Nugegoda, Sri Lanka

**Keywords:** fungal biodiversity, *Dothideomycetes*, drought tolerance plant, multi-loci phylogenetic analysis, new fungal taxa

“The order of authors in the author list of the published paper was erroneously given as:

“Kevin David Hyde, Napalai Chaiwan, Ruvishika Shehali Jayawardena, Saowaluck Tibpromma, Dhanushka N. Wanasinghe, Ishara Sandeepani ManawasingheIshara, Dimuthu S. Manamgoda, Itthayakorn Promputtha”

The correct author list reads:

“Napalai Chaiwan, Kevin David Hyde, Ruvishika Shehali Jayawardena, Saowaluck Tib-promma, Dhanushka Wanasinghe, Ishara Sandeepani Manawasinghe, Dimuthu S. Manamgoda, Itthayakorn Promputtha”

