# Supplementary material for: Morphology and molecular phylogeny of Dothideomycetes fungi associated with Dracaena plants
Source: Front Cell Infect Microbiol. 2025 Aug 11;15:1550824. doi: 10.3389/fcimb.2025.1550824 (PMC12375623; doi:10.3389/fcimb.2025.1550824)
Supplement: Supplementary file 1 [file SupplementaryFile1.docx]

**10 Figure Legends**

**
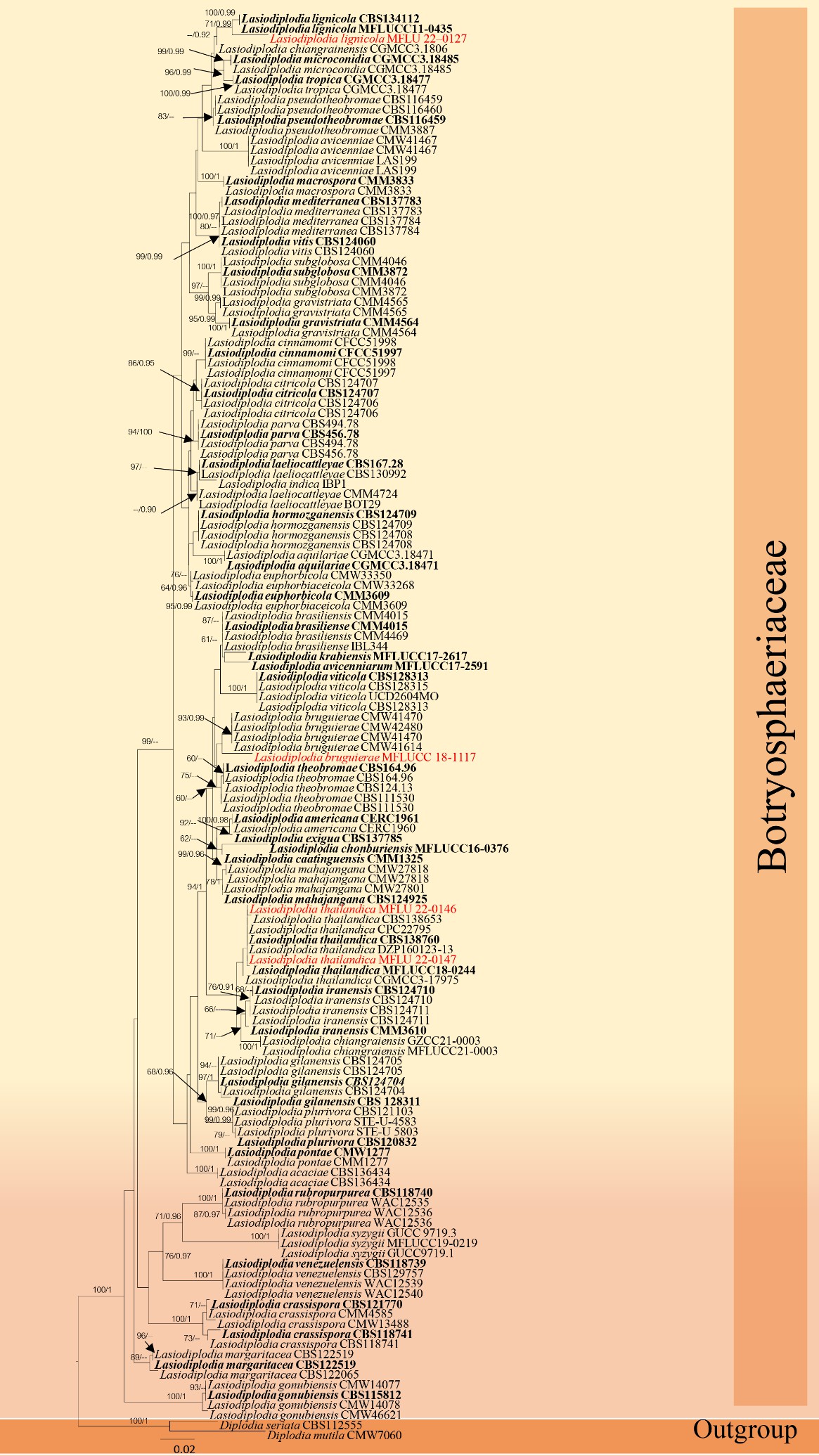
**

| \| **FIGURE 1 \|** Phylogram generated from RAxML analysis based on combined ITS and *tef1-α* sequence data of selected *Botryosphaeriaceae* isolates. Maximum likelihood bootstrap support values ≥ 60% (ML) and Bayesian pos-terior probabilities (PP) ≥ 0.90 are given. The scale bar indicates 0.02 changes. The isolates obtained in this study are in red and ex-type taxa are in black bold. \|  \| \| --- \| --- \| \|  \|  \| \|  \|  \| |
| --- | --- | --- | --- | --- | --- | --- |


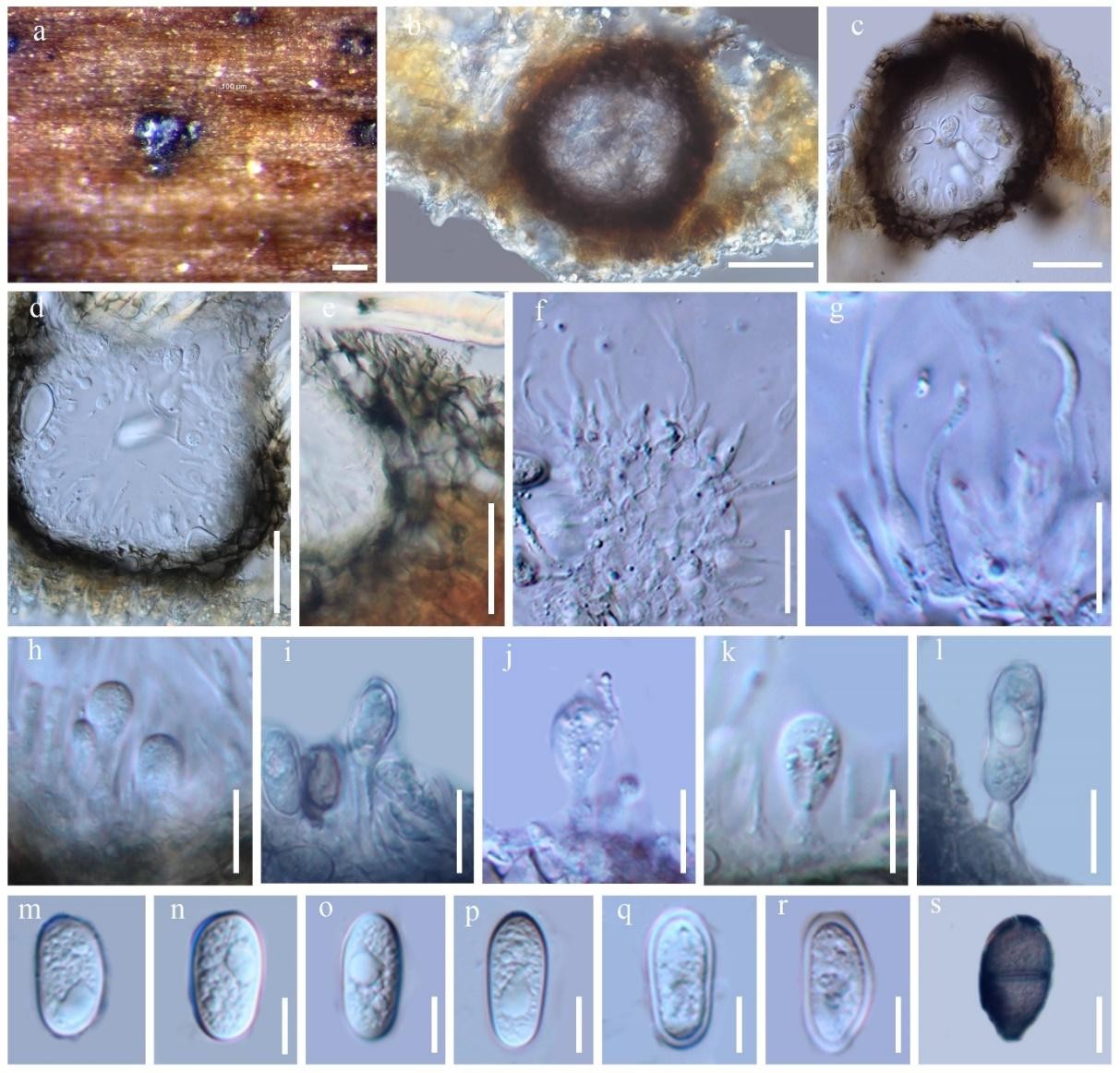
**FIGURE 2 |** *Lasiodiplodia bruguierae* (MFLU 22–0183) (**a**) Conidiomata on dead leaves of *Dracaena fragrans*. (**b–c**). Conidioma. (**d–e**). Conidioma wall. (**f–g**) Paraphyses. (**h–l**) Conidiogenous cells. (**m– r**) Conidia **s**. Mature conidia. Scale bars: (**a**) =100 μm. (**b–d**) = 50 μm. (**e–g**) = 20 μm. (**h–s**) = 10 μm.


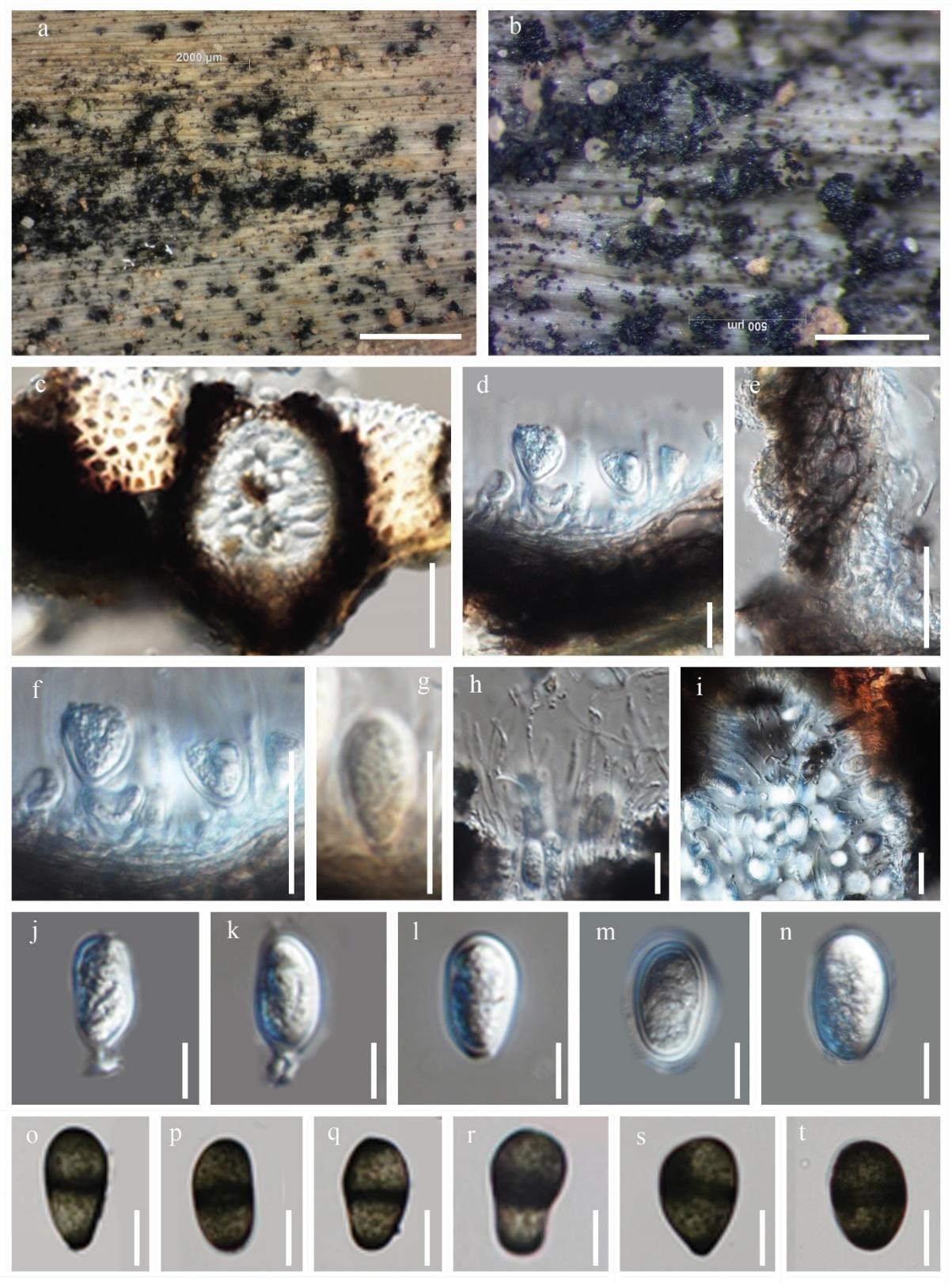


|  |  |
| --- | --- |
|  | **FIGURE 3 \|** *Lasiodiplodia lignicola* (MFLU 22–0127, holotype) (**a**–**b**) Fruiting body on dead leaves |
|  | of *Dracaena fragrans*. (**c**) Section through conidioma. (**d**–**e**) Peridium (Conidioma wall). (**f**–**g**) |
|  | Conidiogenous cells. (**h**–**i**) Paraphyses. (**j–n**) Conidia (**o–t**). Mature conidia. Scale bars: (**a**)=2000 μm. |
|  | (**b**)=500 μm. (**c, g, i**) =20 μm. (**d, f, h, j, k, l, m, n, o-t**) =10 μm. (**d, e**) = 5 μm. |


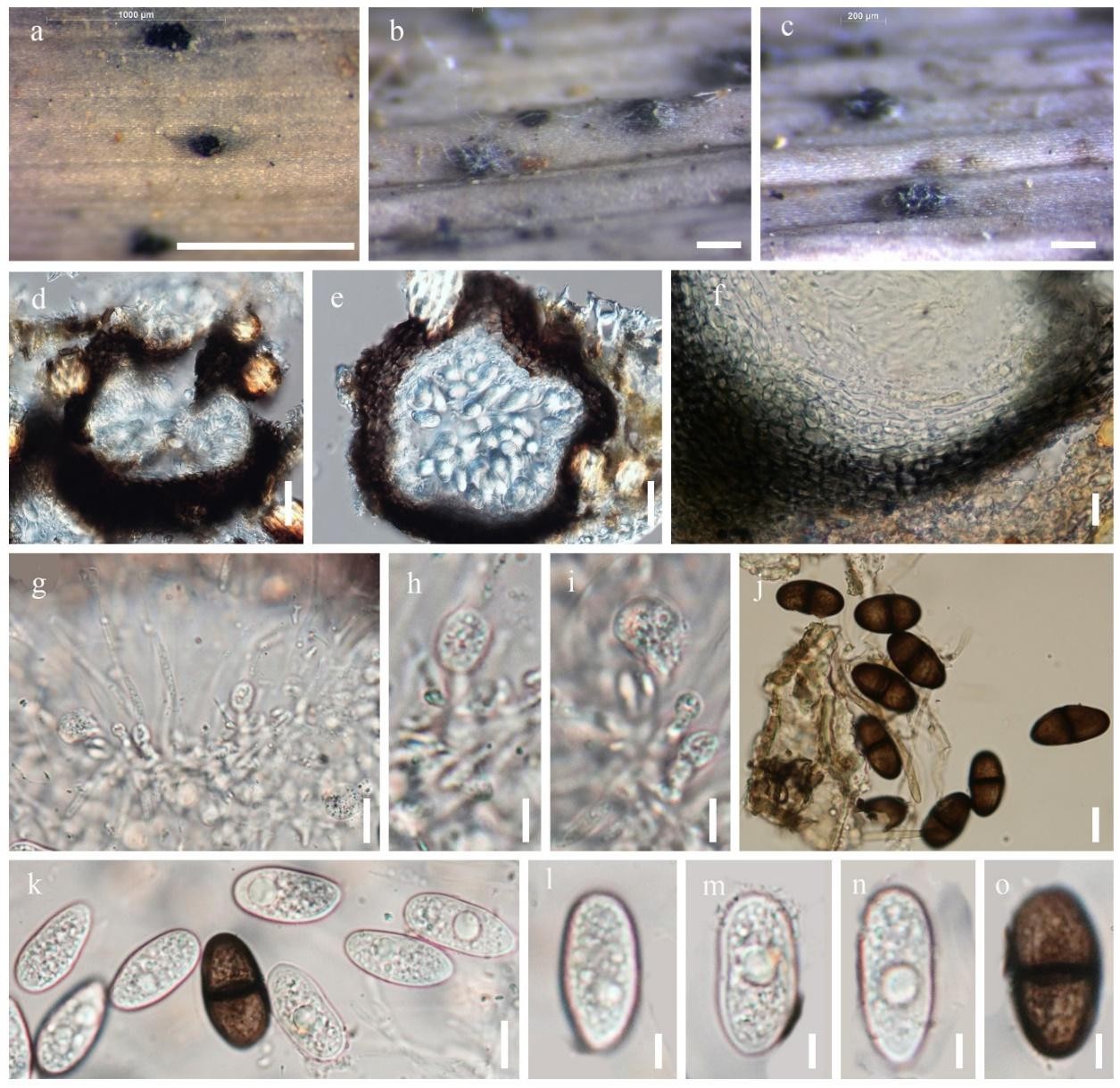


|  |  |
| --- | --- |
|  | **FIGURE 4 \|** *Lasiodiplodia thailandica* (MFLU 22–0146) (**a–c**) Conidiomata on dead leaves of |
|  | *Dracaena fragrans*. (**d–e**) Section through conidioma (**f**) Conidioma wall (**g–i**) Conidiogenous cells |
|  | (**j–o**) Conidia and mature conidia Scale bars: (**a**) = 1000 μm. (**b, c**) = 200 μm. (**d–g, j–o**) = 10 μm. (**h–** |
|  | **i**) = 5 μm. |


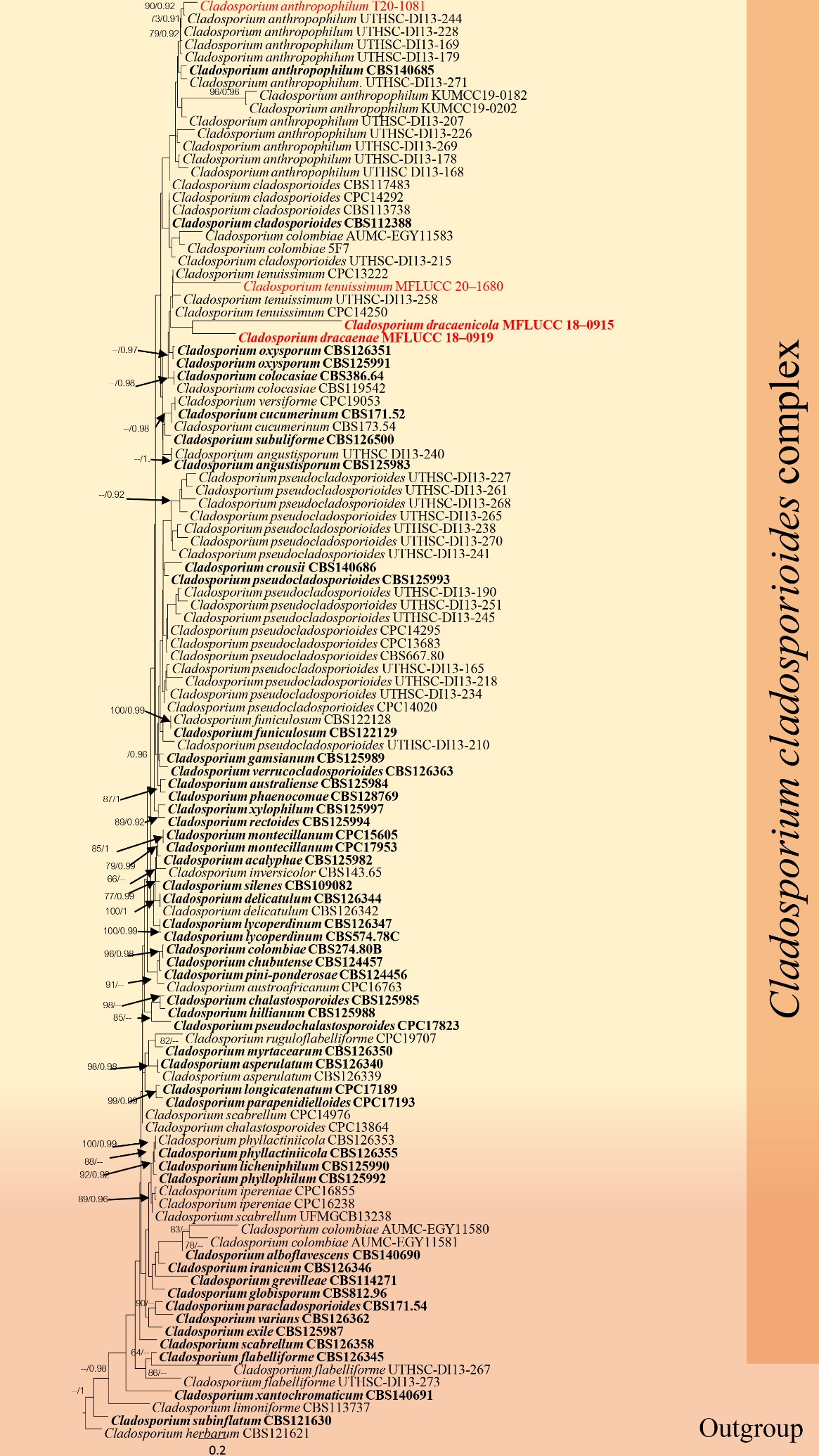


|  | **FIGURE 5 \|** Phylogram generated from RAxML analysis based on combined ITS, *tef1-α* and *act* |
| --- | --- |
|  | sequence data of selected *Cladosporiaceae* isolates. Maximum likelihood bootstrap support values ≥ |
|  | 60% (ML) and Bayesian pos-terior probabilities (PP) ≥ 0.90 are given. The scale bar indicates 0.2 |
|  | changes. The isolates obtained in this study are in red and ex-types taxa are in black bold. |
|  |  |


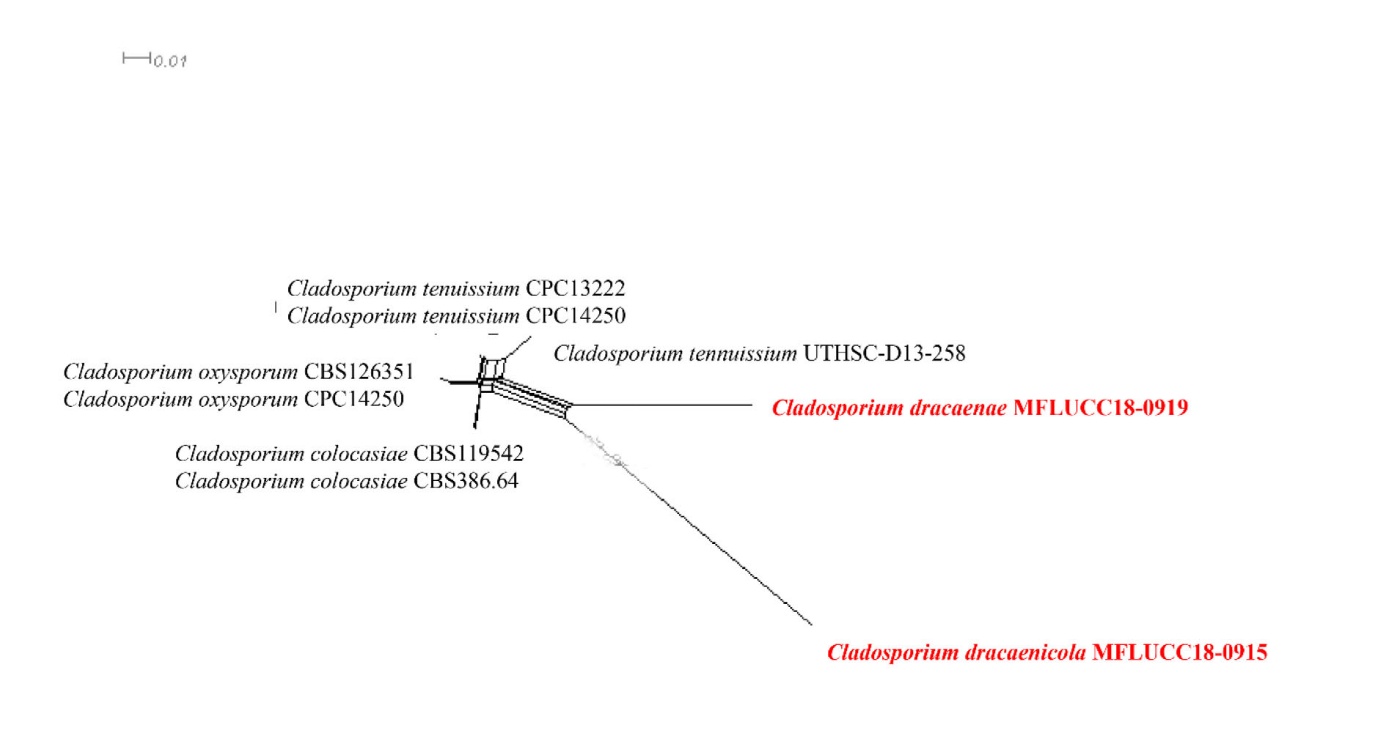


**FIGURE 6 |** Results of the pairwise homoplasy index (PHI) test of *Cladosporium dracaenae*, *Cladosporium dracaenicola* and closely related species using both LogDet transformation and splits decomposition. PHI test results (Φw) < 0.05 indicate significant recombination within the dataset. The new taxon is in red bold type. P=1.


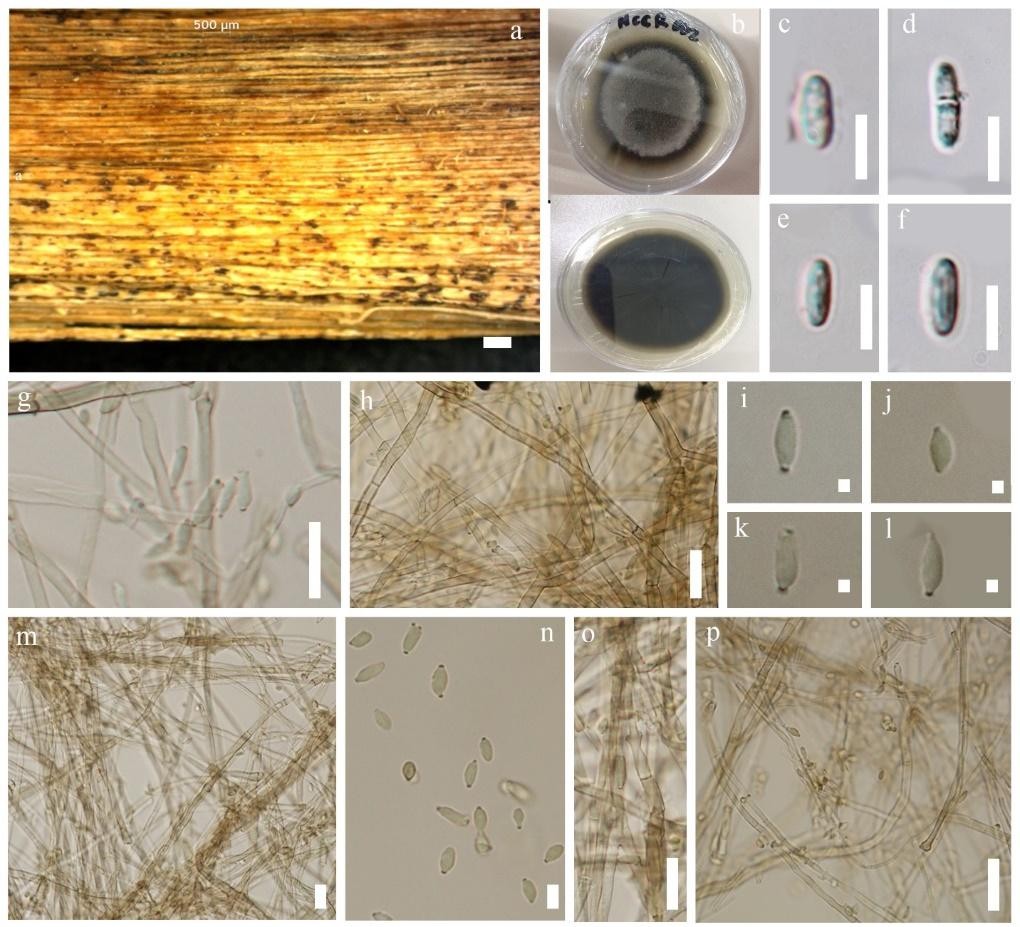
**FIGURE 7 |** *Cladosporium dracaenae* (MFLU 22–0185). (**a**) Appearance on the substrate. (**b**) Culture: The mycelium grows 5-6 cm within 28 days on potato dextrose agar (PDA). (**c–f, i–l, n**) Conidia. (**g– h, m, o–p**) Conidiophore and Conidiogenous cell. Scale bars: (**a**) = 500 μm. (**c–h, m–p**) = 10 μm. (**i– l**) =5 μm.


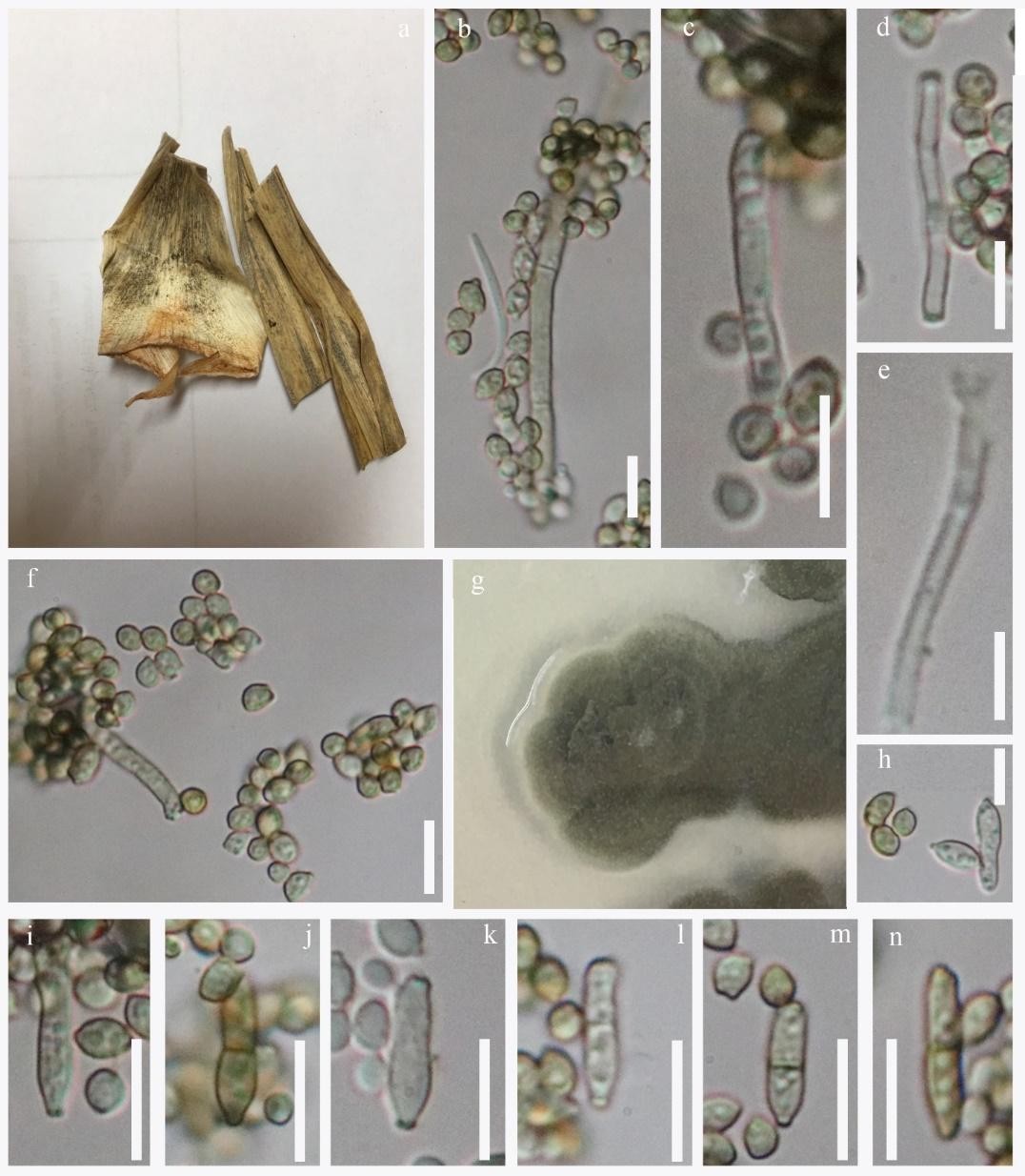
**FIGURE 8 |** *Cladosporium dracaenicola* (MFLU 22–0186). (**a**) Appearance on the substrate. (**b–e**) Conidiophore and conidia. (**f**) Conidia. (**g**) Culture: The mycelium grows 3-4 cm within 14 days on Potato dextrose agar (PDA) (front view). (**h, i–n**) Conidia and ramoconidia. Scale bars: (**b–f, i–n**) =10 μm. (**h**) = 5 μm.


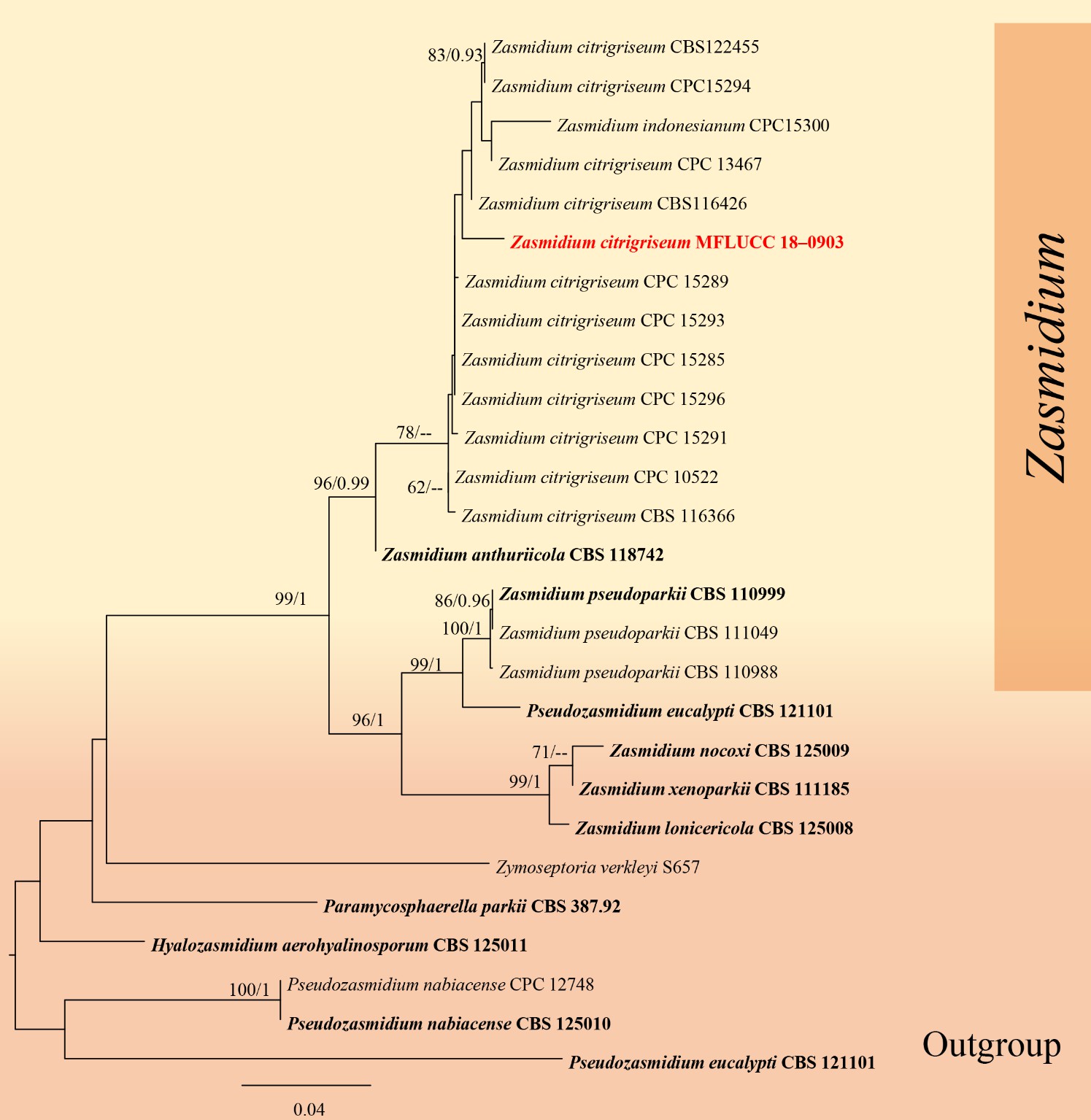


|  |  |
| --- | --- |
|  | **FIGURE 9 \|** Phylogram generated from RAxML analysis based on combined LSU, ITS and *act* |
|  | sequence data of selected *Mycosphaerellaceae* isolates. Maximum likelihood bootstrap support values |
|  | ≥ 60% (ML) and Bayesian pos-terior probabilities (PP) ≥ 0.90 are given. The scale bar indicates 0.04 |
|  | changes. The isolates obtained in this study are in red and ex-types taxa are in black bold. |


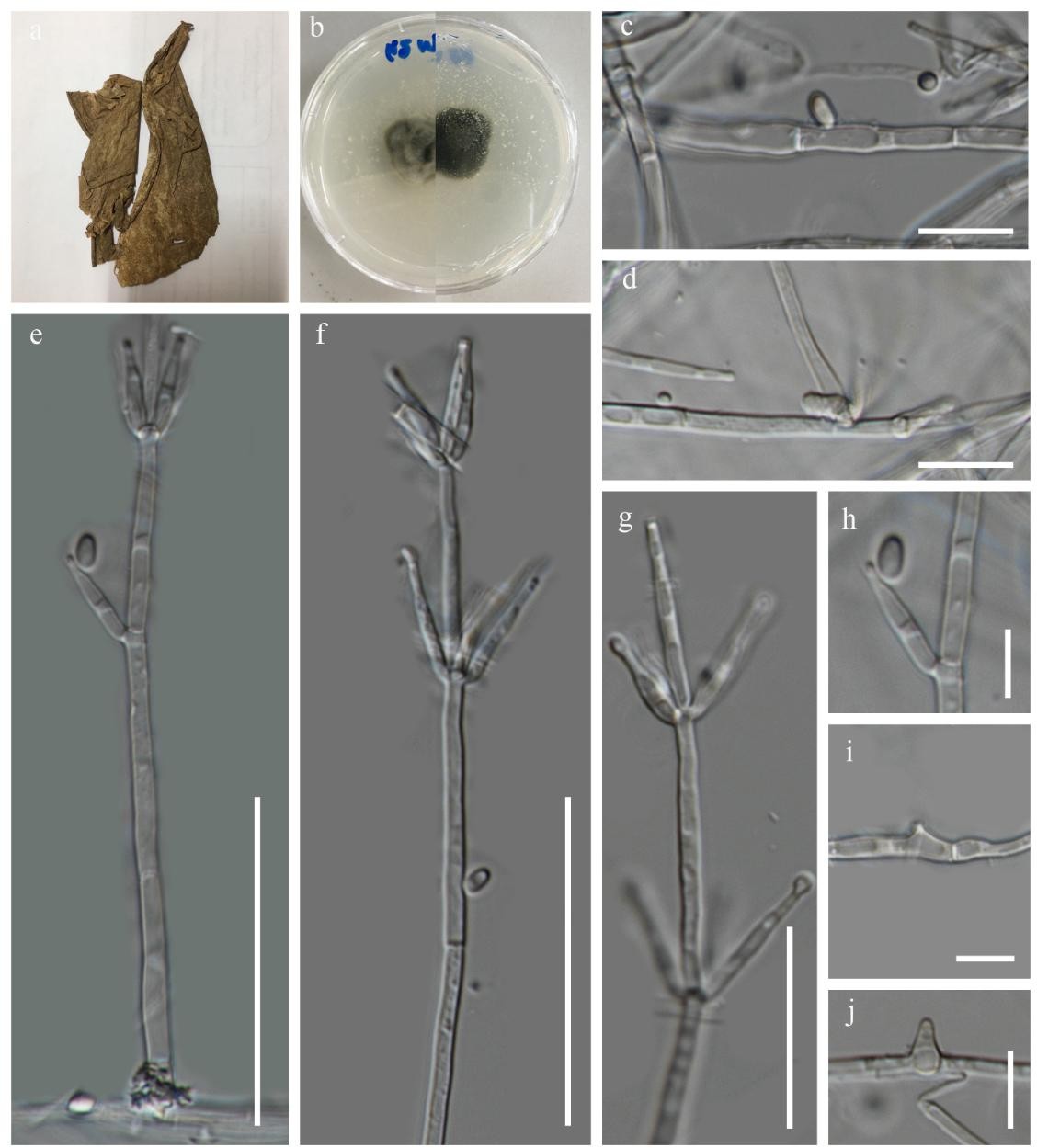


|  |  |
| --- | --- |
|  | **FIGURE 10 \|** *Zasmidium citrigriseum* (MFLU 22–0189). (**a**) Appearance on the substrate (**b**) Culture: |
|  | The mycelium grows 3-4 cm within 14 days on Potato dextrose agar (PDA). (**c, d**) Conidiogenous cell |
|  | attached on hypha. (**e–h**) Conidiophores and conidium. (**i–j**) Conidiogenous cells. Scale bars: (**e, f**) = |
|  | 50 μm. (**g**) = 20 μm. (**c, d, h, j**) = 10 μm. |


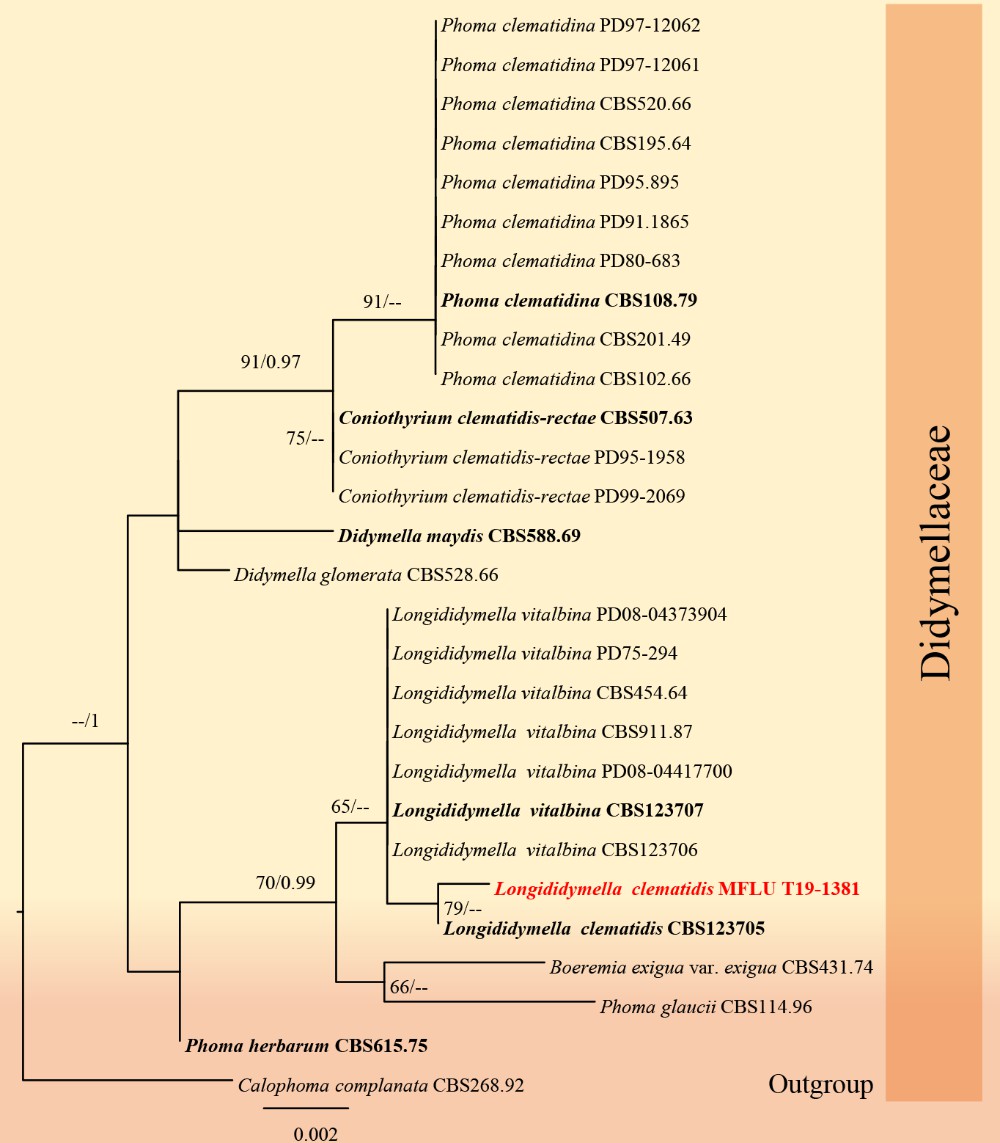


|  |  |
| --- | --- |
|  | **FIGURE 11 \|** Phylogram generated from RAxML analysis based on LSU sequence data of selected |
|  | *Didymellaceae* isolates. Maximum likelihood bootstrap support values ≥ 60% (ML) and Bayesian |
|  | pos-terior probabilities (PP) ≥ 0.90 are given. The scale bar indicates 0.002 changes. The isolates |
|  | obtained in this study are in red and extypes taxa are in black bold. |


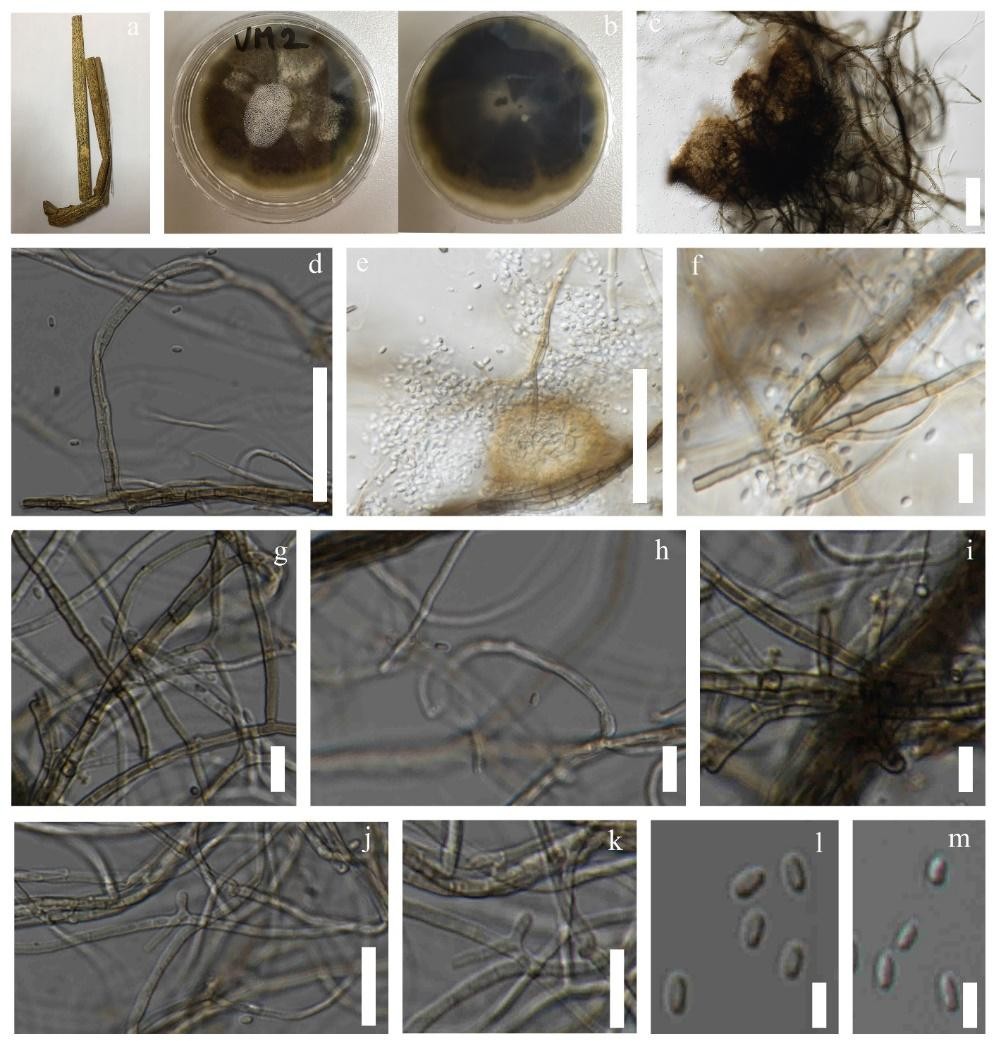


|  |  |
| --- | --- |
|  | **FIGURE 12 \|** *Longididymella clematidis* (MFLU 22–0187). (**a**) Appearance on the substrate (**b**) |
|  | Culture: The mycelium grows 3-4 cm within 14 days on Potato dextrose agar (PDA). (**c**–**d**) |
|  | Conidiogenous cell attached on hypha. (**e–h**) Conidiophores and conidium. (**i–m**) Conidiogenous cells |
|  | and conidium. Scale bars: (**c, d, f**) =50 μm. (**e, g, h-j**) = 20 μm. (**k, l, m**) = 10 μm. |


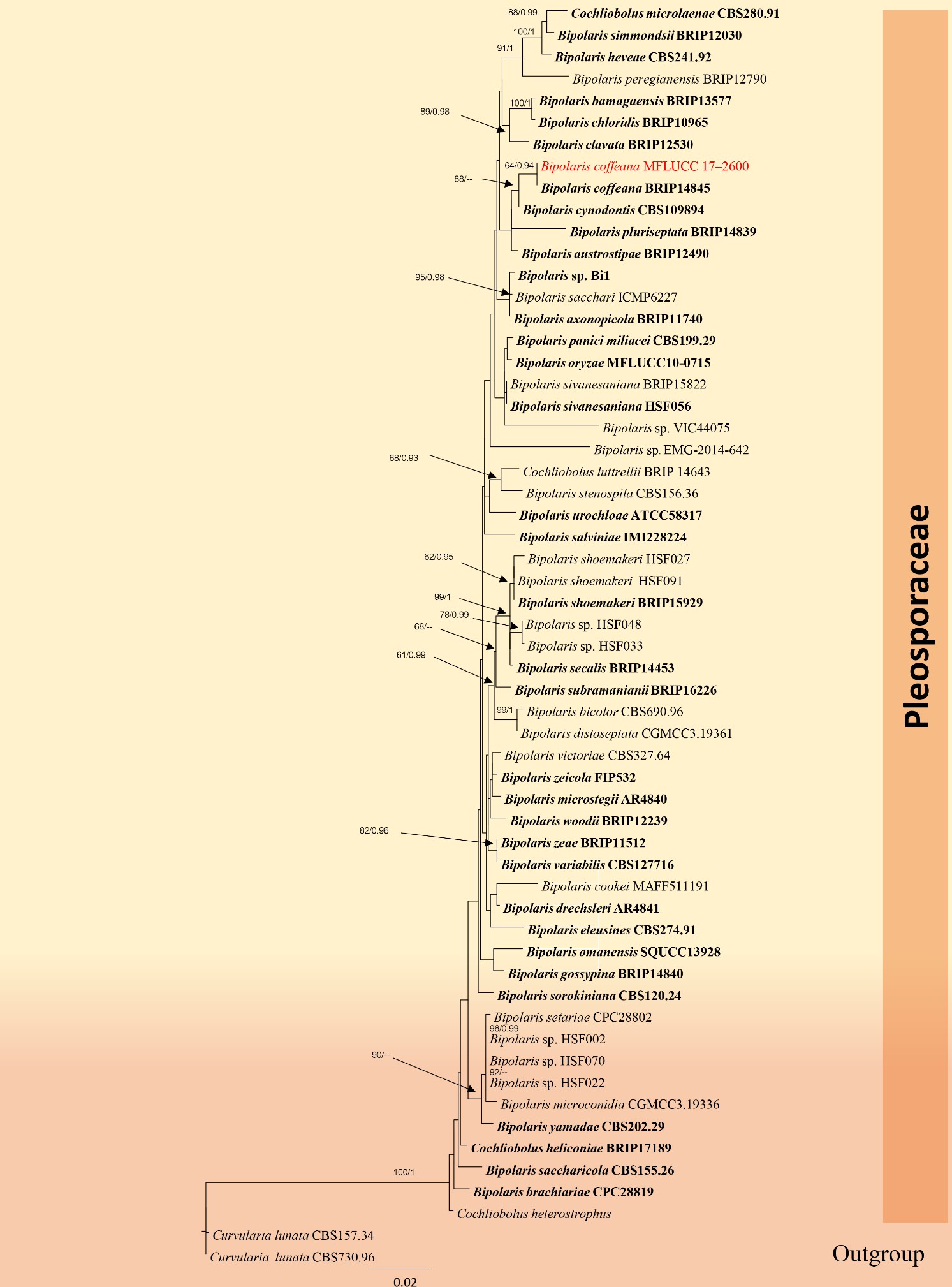


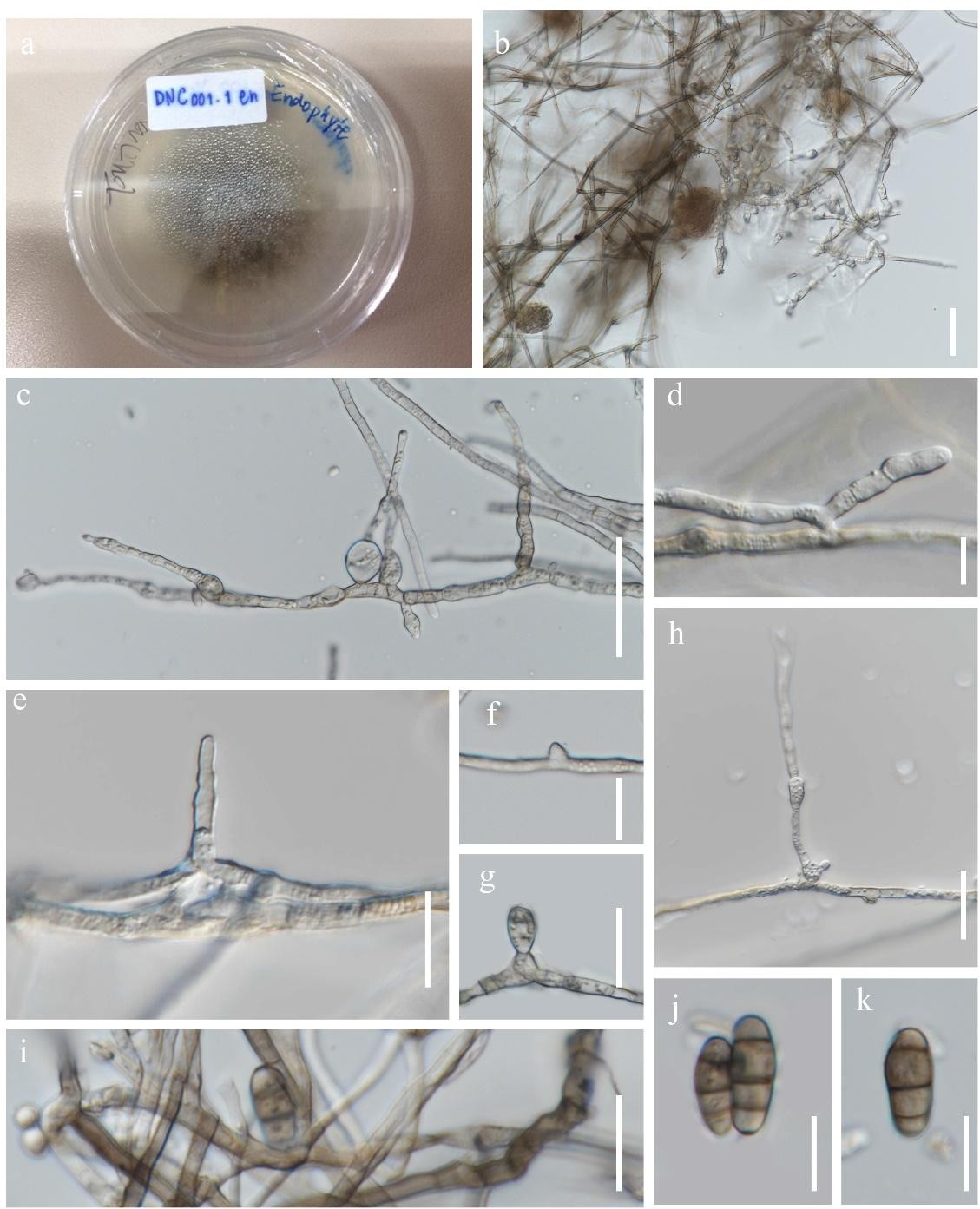


| **FIGURE 13 \|** Phylogram generated from RAxML analysis based on combined ITS and *tef1-α* |
| --- |
| sequence data of selected *Pleosporaceae* isolates. Maximum likelihood bootstrap support values ≥ |
| 60% (ML) and Bayesian pos-terior probabilities (PP) ≥ 0.90 are given. The scale bar indicates 0.02 |
| changes. The isolates obtained in this study are in red and extypes taxa are in black bold. |

|  |  |
| --- | --- |
|  | **FIGURE 14 \|** *Bipolaris coffeana* (MFLUCC 17–2600). (**a**) Culture. (**b, c, d**) Conidiogenous cell |
|  | attached on hypha. (**e**–**h**) Conidiophore. (**i**–**k)** Conidiogenous cell. (**h**) Conidia. Scale bars: (**c**) = 200 |
|  | μm. (**b, d**–**h**) =100 μm. (**c, g**) = 50 μm. (**i, j, k**) =10 μm. |


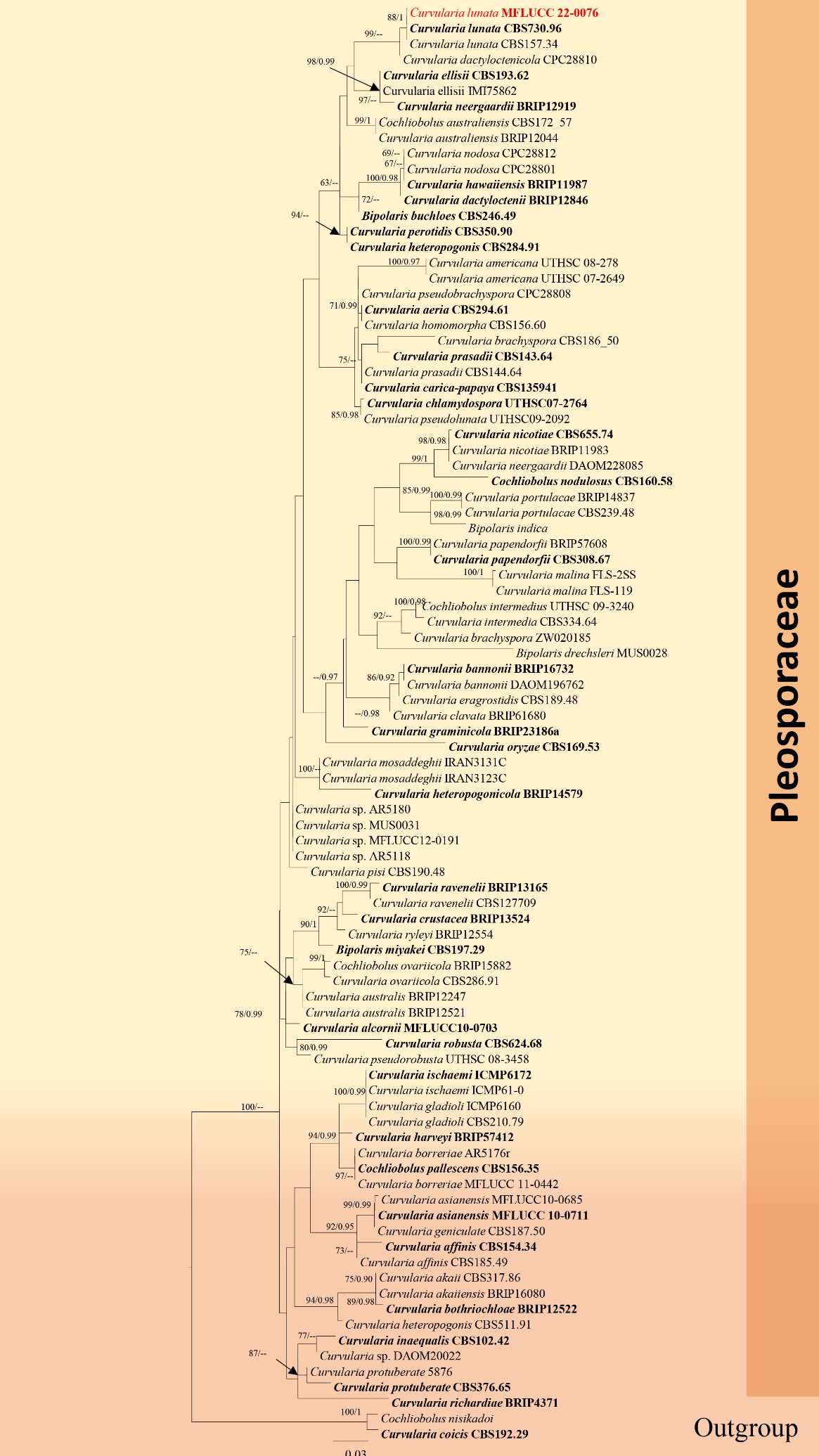


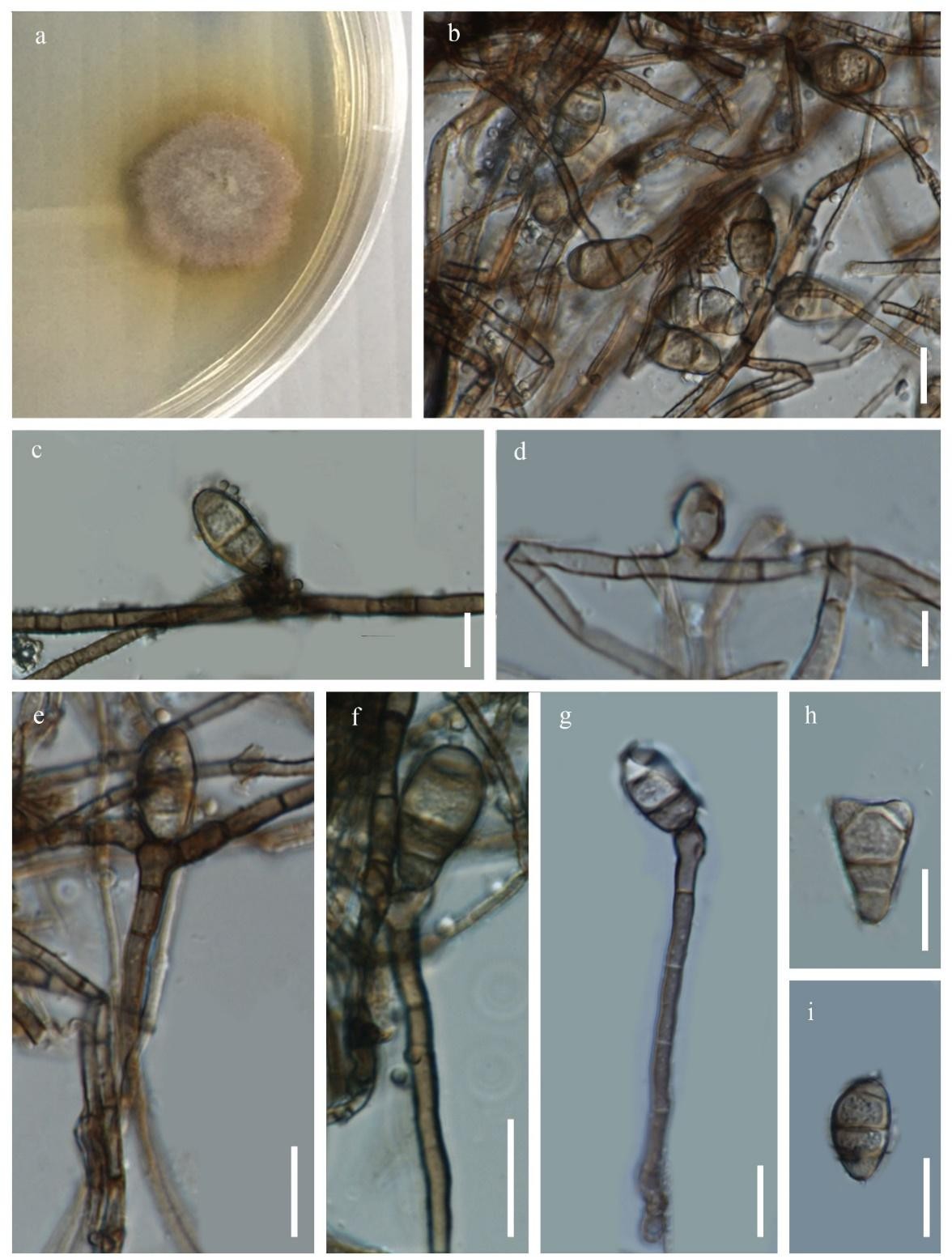


|  | **FIGURE 15 \|** Phylogram generated from RAxML analysis based on combined ITS sequence data of |
| --- | --- |
|  | selected *Pleosporaceae* isolates. Maximum likelihood bootstrap support values ≥ 60% (ML) and |
|  | Bayesian pos-terior probabilities (PP) ≥ 0.90 are given. The scale bar indicates 0.03 changes. The |
|  | isolates obtained in this study are in red and extypes taxa are in black bold. |
|  |  |

|  |  |
| --- | --- |
|  | **FIGURE 16 \|** *Curvularia lunata* (MFLUCC 22–0076, living culture). (**a**) Culture: The mycelium |
|  | grows 2-3 cm within 7 days on Potato dextrose agar (PDA). (**b**) Appearance of mycelium on culture. |
|  | (**c**–**d**) Conidiogenous cell attached on hypha (**e**–**g**) Conidiophore (**i–j)** Conidiogenous cell (**h**) Conidia |
|  | Scale bars: (**b-c, e-i**) =100 μm. (**d**)=50 μm. |


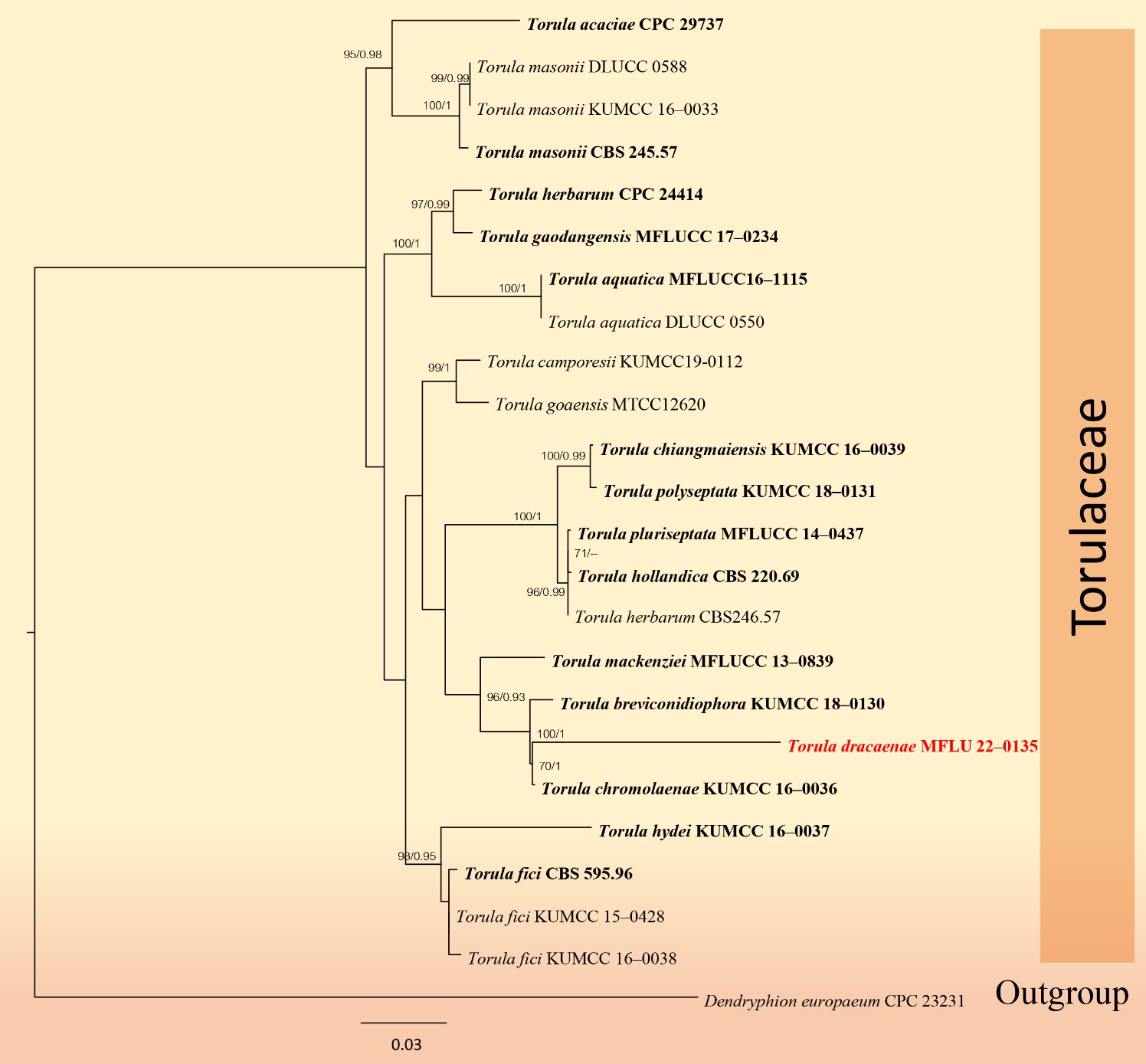
**FIGURE 17 |** Phylogram generated from RAxML analysis based on combined ITS, LSU and SSU sequence data of selected *Torulaceae* isolates. Maximum likelihood bootstrap support values ≥ 60% (ML) and Bayesian pos-terior probabilities (PP) ≥ 0.90 are given. The scale bar indicates 0.03 changes. The isolates obtained in this study are in red and extypes taxa are in black bold.


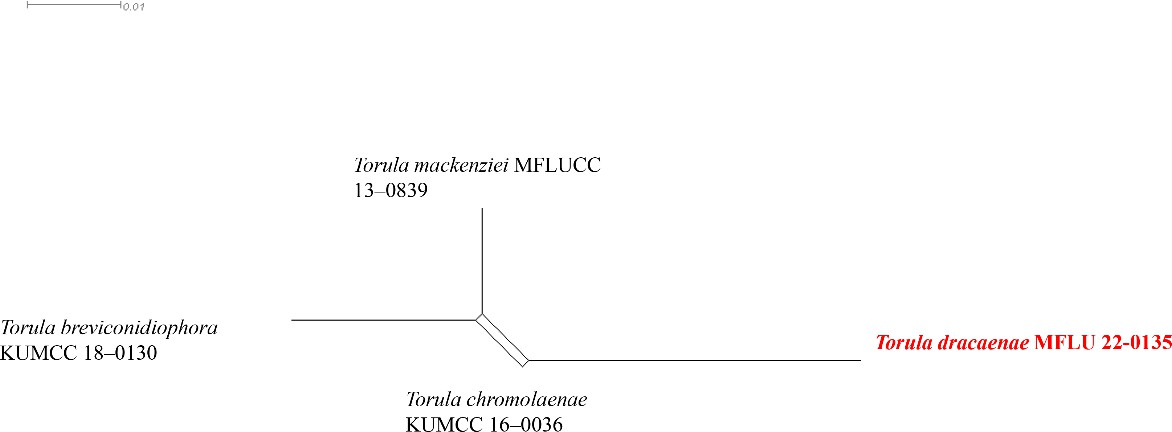


**FIGURE 18 |** Results of the pairwise homoplasy index (PHI) test of *Torula dracaenae* and closely related species using both LogDet transformation and splits decomposition. PHI test results (Φw) <

0.05 indicate significant recombination within the dataset. The new taxon is in red bold type. P=0.1959.


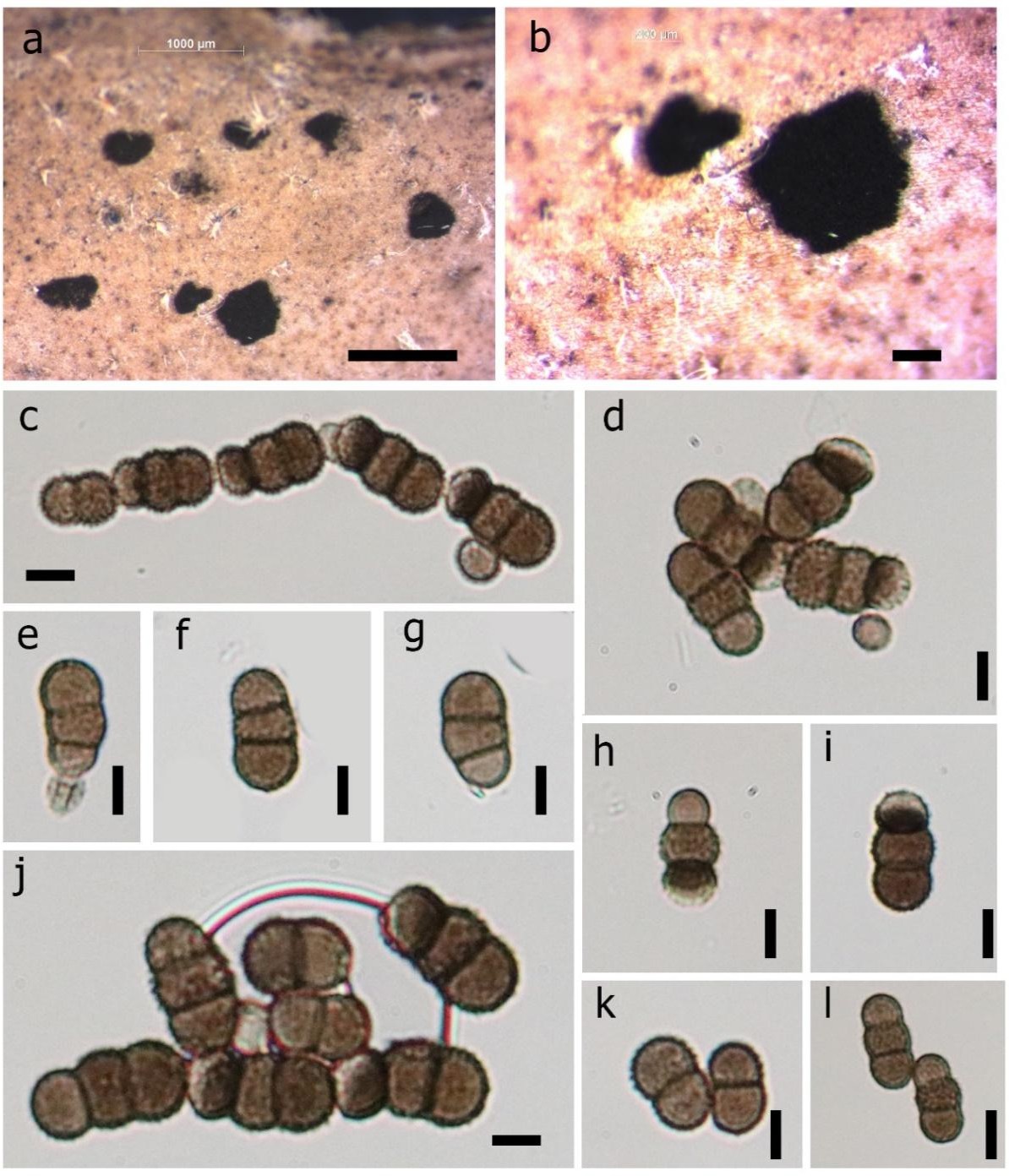
**FIGURE 19 |** *Torula dracaenae* (MFLU 22–0135, holotype) (**a, b**) Appearance of fruiting body on host surface. (**c–l**) Conidia (**e**) Conidia. Scale bars: (**a**) = 1000 µm, (**b**) = 200 μm, (**c–l**) = 10 μm.


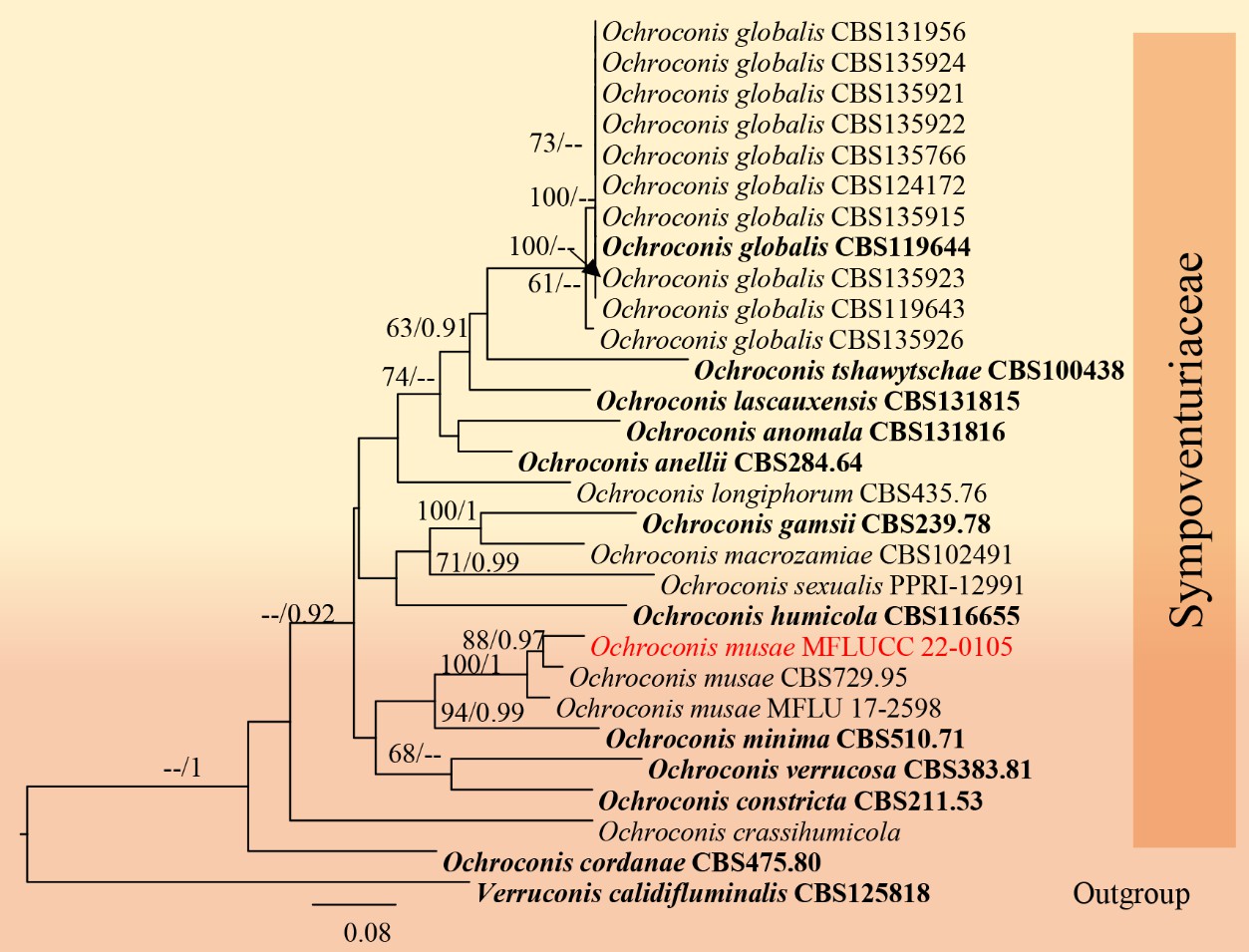
**FIGURE 20 |** Phylogram generated from RAxML analysis based on combined LSU, ITS and *tub* sequence data of selected *Didymellaceae* isolates. Maximum likelihood bootstrap support values ≥ 60% (ML) and Bayesian posterior probabilities (PP) ≥ 0.90 are given. The scale bar indicates 0.08 changes. The isolates obtained in this study are in red and extypes taxa are in black bold.


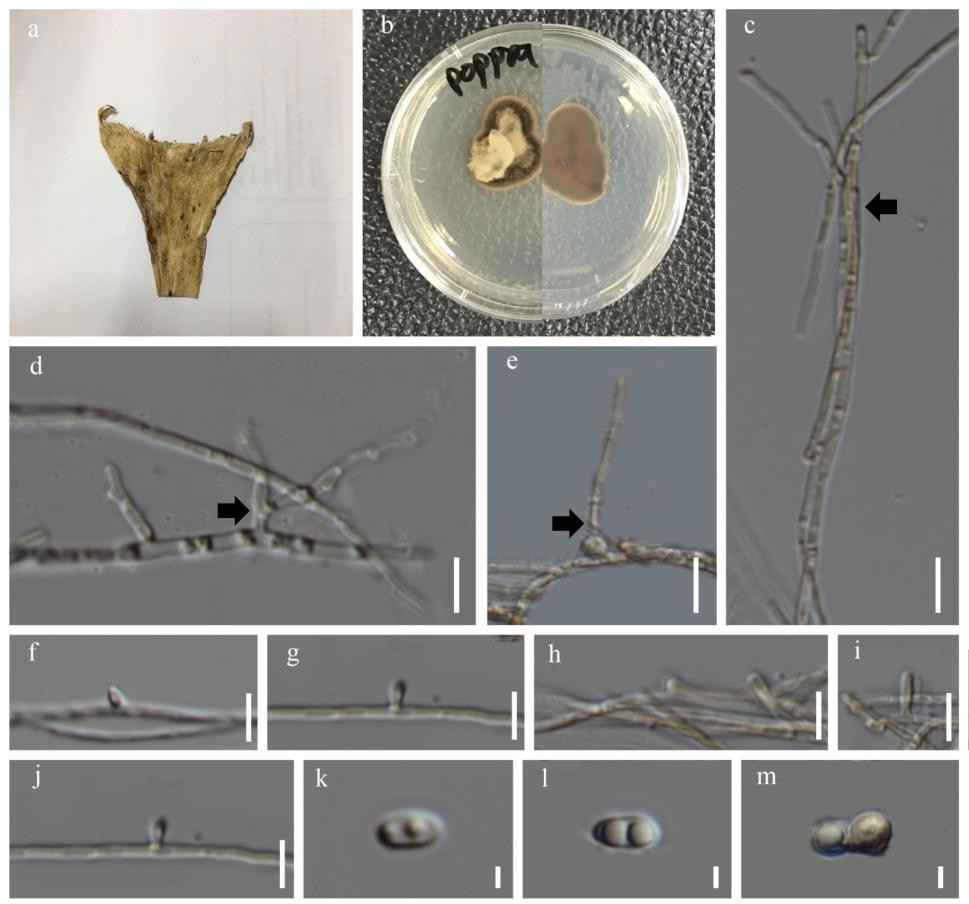
**FIGURE 21 |** *Ochroconis musae* (MFLU 22–0188). (**a**) Appearance on the substrate. (**b**) Culture: The mycelium grows 2-3 cm within 14 days on Potato dextrose agar (PDA). (**c–e**) Conidiophores. (**f–j**) Conidiogenous cell. (**k–m**) Conidia. Scale bars: (**c–m**) = 10 µm.

**Tables 1.** Details of the gene loci sequences and primers used in this study are provided.

| **Gene/loci** | **PCR primers (forward/reverse)** | **References for primer** |
| --- | --- | --- |
| *Actin gene (act)* | (ACT512F/ACT783R) | (Carbone and kohn, 1999) |
| *Internal transcribed spacer 1, 5.8S*  *ribosomal RNA gene (ITS)* | (ITS4/ITS5) | (White et al.1990) |
| *28S ribosomal RNA gene (LSU)* | (LR0R/LR5) | (Vilgalys and Hester, 1990) |
| *Elongation factor-1 alpha gene (tef1-α)* | (EF1-983F/EF1-2218R) | (Rehner and Bukley, 2005) |
| *Beta-tubulin gene (tub)* | (Bt2a/Bt2b) | (Glass and Donaldson, 1995) |

**Table 2.** Taxa names, strain numbers and GenBank accession numbers of the ITS and *tef1-α* and sequences used in the phylogenetic analyses of *Botryosphaeriaceae*. The newly generated sequences are indicated in red, while the type strains are in black bold font. “–” indicates unavailable sequences.

| **Taxon name** | **Voucher/Culture** | **GenBank Accession Number** | |
| --- | --- | --- | --- |
|  |  | **ITS** | *tef1-α* |
| ***Lasiodiplodia aquilariae*** | **CGMCC3.18471** | **OM614886** | **KY848600** |
| *Lasiodiplodia avicenniae* | CMW41467 | KP860835 | KP860680 |
| *Lasiodiplodia avicenniae* | LAS199 | KU587957 | KU587947 |
| ***Lasiodiplodia avicenniarum*** | **MFLUCC17**–**2591** | **NR_163344** | **MK340867** |
| ***Lasiodiplodia brasiliensis*** | **CMM4015** | **NR_147338** | – |
| *Lasiodiplodia brasiliensis* | CMM4469 | PP930491 | PP935223 |
| *Lasiodiplodia bruguierae* | CMW41470 | KP860833 | KP860678 |
| *Lasiodiplodia bruguierae* | CMW42480 | KP860832 | KP860677 |
| *Lasiodiplodia chiangraiensis* | MFLUCC21–0003 | MW815630 | MW760854 |
| *Lasiodiplodia chiangraiensis* | GZCC21–0003 | MW760853 | – |
| *Lasiodiplodia chonburiensis* | MFLUCC16–0376 | MH275066 | MH412773 |
| ***Lasiodiplodia cinnamomi*** | **CFCC51997** | **MG866028** | **MH236799** |
| *Lasiodiplodia cinnamomi* | CFCC51998 | MG866029 | MH236800 |
| ***Lasiodiplodia citricola*** | **CBS124707** | **NR_137048** | – |
| *Lasiodiplodia crassispora* | CBS118741 | NG_062741 | EU673303 |
| *Lasiodiplodia crassispora* | CMW13488 | DQ103552 | DQ103559 |
| *Lasiodiplodia crassispora* | CBS121770 | NR_136993 | EU101352 |
| ***Lasiodiplodia euphorbiaceicola*** | **CMM3609** | **NR_147347** | – |
| *Lasiodiplodia euphorbiaceicola* | CMW_3268 | PQ222585 | PQ227198 |
| *Lasiodiplodia gilanensis* | CBS124704 | KU696357 | KU887511 |
| *Lasiodiplodia gilanensis* | CBS124705 | KU696356 | KU887510 |
| *Lasiodiplodia gilanensis* | CBS128311 | OM367913 | OM387011 |
| *Lasiodiplodia gonubiensis* | CMW14077 | AY639595 | DQ103566 |
| *Lasiodiplodia gonubiensis* | CMW14078 | AY639594 | DQ103567 |
| ***Lasiodiplodia gravistriata*** | **CMM4564** | **KT250949** | **KT250950** |
| *Lasiodiplodia gravistriata* | CMM4565 | KT250947 | KT266812 |
| ***Lasiodiplodia hormozganensis*** | **CBS124709** | **PP465988** | **PP460491** |
| *Lasiodiplodia hormozganensis* | CBS124708 | KU696360 | KU887514 |
| *Lasiodiplodia iranensis* | CBS_24710 | OQ826628 | PP966929 |
| *Lasiodiplodia iranensis* | CBS124711 | OQ067613 | OQ076935 |
| *Lasiodiplodia iranensis* | CMM3610 | NR_147348 | – |
| ***Lasiodiplodia krabiensis*** | **MFLUCC17**–**2617** | **MN047093** | **MN077070** |
| *Lasiodiplodia laeliocattleyae* | CBS130992 | PP465986 | PP460493 |
| ***Lasiodiplodia laeliocattleyae*** | **CBS167.28** | **PP465987** | **PP460494** |
| *Lasiodiplodia lignicola* | CBS134112 | PP778677 | PP779531 |
| *Lasiodiplodia lignicola* | CGMCC3.18061 | MT934413 | MT920442 |
| *Lasiodiplodia macrospora* | CMM3833 | KF234557 | KF226718 |
| *Lasiodiplodia mahajangana* | CMW27801 | FJ900595 | FJ900641 |
| *Lasiodiplodia mahajangana* | CMW27818 | FJ900596 | FJ900642 |
| *Lasiodiplodia mahajangana* | CBS137785 | NR_147353 | – |
| *Lasiodiplodia margaritacea* | CBS122519 | EU144050 | EU144065 |
| *Lasiodiplodia mediterranea* | CBS137783 | NR_147352 | – |
| *Lasiodiplodia mediterranea* | CBS137784 | KU578252 | KU720487 |
| *Lasiodiplodia microcondia* | CGMCC3.18485 | KY783441 | KY848614 |
| ***Lasiodiplodia parva*** | **CBS456.78** | **MH861166** | – |
| *Lasiodiplodia parva* | CBS494.78 | NG_062747 | – |
| *Lasiodiplodia plurivora* | STE-U5803 | EF445362 | EF445395 |
| *Lasiodiplodia plurivora* | STE-U4583 | – | EF445396 |
| *Lasiodiplodia pontae* | CMM1277 | MK510560 | MK510671 |
| *Lasiodiplodia pseudotheobromae* | CBS116459 | EF622077 | EF622057 |
| *Lasiodiplodia pseudotheobromae* | CBS116460 | EF622078 | EF622058 |
| *Lasiodiplodia rubropurpurea* | WAC12535 | NR_136976 | – |
| *Lasiodiplodia rubropurpurea* | WAC12536 | DQ103554 | DQ103572 |
| *Lasiodiplodia subglobosa* | CMM3872 | KF234558 | KF226721 |
| *Lasiodiplodia subglobosa* | CMM4046 | NR_147350 | – |
| *Lasiodiplodia syzygii* | GUCC9719.1 | – | MW087103 |

| *Lasiodiplodia thailandica* | CBS138760 | OR759966 | OR767664 |
| --- | --- | --- | --- |
| *Lasiodiplodia thailandica* | CBS138653 | – | OQ50910 |
| *Lasiodiplodia thailandica* | CGMCC3.17975 | KY767663 | – |
| *Lasiodiplodia thailandica* | MFLUCC18-0244 | KY767662 | – |
| *Lasiodiplodia theobromae* | CBS164.96 | OR077890 | MT975693 |
| *Lasiodiplodia theobromae* | CBS111530 | EF622074 | EF622054 |
| *Lasiodiplodia tropica* | CGMCC3.18477 | KY783454 | KY848616 |
| *Lasiodiplodia venezuelensis* | WAC12539 | DQ103547 | DQ103568 |
| *Lasiodiplodia venezuelensis* | WAC12540 | DQ103548 | DQ103569 |
| *Lasiodiplodia viticola* | CBS128313 | MH864855 | – |
| *Lasiodiplodia viticola* | UCD2604MO | OK087687 | – |
| *Lasiodiplodia vitis* | CBS124060 | KX464148 | KX464642 |
| *Lasiodiplodiaacaciae* | CBS136434 | MT587421 | MT592133 |
| ***Lasiodiplodia americana*** | **CERC1961_CFCC_50065_** | **KP217059** | **KP217067** |
| *Lasiodiplodia americana* | CERC1960 CFCC50064 | KP217058 | KP217066 |
| *Lasiodiplodia aquilariae* | CGMCC3.18471 | OM614886 | OM681514 |
| *Lasiodiplodia avicenniae* | CMW41467 | KP860835 | KP860680 |
| *Lasiodiplodia avicenniae* | LAS199 | KU587957 | KU587947 |
| *Lasiodiplodia brasiliense* | CMM4015 | JX464063 | JX464049 |
| *Lasiodiplodia brasiliense* | IBL344 | KT151808 | KT151802 |
| *Lasiodiplodia bruguierae* | CMW41470 | KP860833 | KP860678 |
| *Lasiodiplodia bruguierae* | MFLUCC18-1117 | **OM919717** | **OP099910** |
| *Lasiodiplodia bruguierae* | CMW41614 | KP860834 | KP860679 |
| *Lasiodiplodia cinnamomi* | CFCC51997 | MG866028 | MH236799 |
| *Lasiodiplodia cinnamomi* | CFCC51998 | MG866029 | MH236800 |
| *Lasiodiplodia citricola* | CBS124707 | KU887505 | KM822729 |
| *Lasiodiplodia citricola* | CBS124706 | OM891500 | KM822728 |
| ***Lasiodiplodia crassispora*** | **CBS_118741** | **NG_062741** | **EU673303** |
| ***Lasiodiplodia crassispora*** | **CBS_121770** | **MG954354** | **MG979520** |
| *Lasiodiplodia euphorbicola* | CMM3609 | NR_147347 | – |
| *Lasiodiplodia euphorbicola* | CMW33350 | KT247491 | KT247493 |
| *Lasiodiplodia gilanensis* | CBS124704 | OM367913 | OM387011 |
| ***Iodiplodia gilanensis*** | **CBS124705** | **OM367912** | **OM387009** |
| ***Lasiodiplodia gonubiensis*** | **CBS_115812_CMW_14077_** | **AY639595** | – |
| *Lasiodiplodia gonubiensis* | CMW46621 | KY445937 | – |
| *Lasiodiplodia gravistriata* | CMM4564 | MH863401 | – |
| *Lasiodiplodia gravistriata* | CMM4565 | MH874918 | – |
| *Lasiodiplodia hormozganensis* | CBS124709 | PP465988 | PP460491 |
| *Lasiodiplodia indica* | IBP1 | KM376151 | – |
| ***Lasiodiplodia iranensis*** | **CBS_124710_IRAN_1520C_** | **MH863401** | – |
| *Lasiodiplodia laeliocattleyae* | CBS167.28 | MH866448 | KU507454 |
| *Lasiodiplodia laeliocattleyae* | CMM4724 | MG954343 | MG979508 |
| ***Lasiodiplodia lignicola*** | **CBS_134112** | – | **KU887003** |
| *Lasiodiplodia lignicola* | MFLUCC22-0127 | **OM919716** | **OP169689** |
| ***Lasiodiplodia macrospora*** | **CMM_3833_** | **KF234557** | **KF226718** |
| ***Lasiodiplodia mahajangana*** | **CBS_124925** | **MH863425** | – |
| ***Lasiodiplodia margaritacea*** | **CBS_122519_CMW_26162_** | **KT852959** | – |
| ***Lasiodiplodia mediterranea*** | **CBS_137783_** | **NR_147352** | – |
| *Lasiodiplodia mediterranea* | CBS137784 | KU578252 | KU720487 |
| ***Lasiodiplodia microconidia*** | **CGMCC3.18485_** | **MW880671** | – |
| *Lasiodiplodia parva* | CBS456.78 | MH861166 | – |
| *Lasiodiplodia parva* | CBS494.78 | NG_062747 | – |
| ***Lasiodiplodia plurivora*** | **CBS120832** | **EF445362** | **EF445395** |
| *Lasiodiplodia plurivora* | CBS121103 | – | EF445396 |
| ***Lasiodiplodia pontae*** | **CMW_1277** | MK510560 | MK51067 |
| ***Lasiodiplodia pseudotheobromae*** | **CBS_116459** | **KF766193** | **KF766472** |
| *Lasiodiplodia pseudotheobromae* | CMM3887 | MH263657 | MH265123 |
| ***Lasiodiplodia rubropurpurea*** | **CBS_118740** | **OR665145** | **OR552396** |

| *Lasiodiplodia rubropurpurea* | WAC12536 | OR665144 | OR552395 |
| --- | --- | --- | --- |
| ***Lasiodiplodia subglobosa*** | **CMM3872_** | **PQ587540** | **PQ510063** |
| *Lasiodiplodia subglobosa* | CMM4046 | PQ587539 | PQ510055 |
| *Lasiodiplodia syzygii* | MFLUCC19-0219 | – | MW016943 |
| *Lasiodiplodia syzygii* | GUCC9719.3 | – | MW087102 |
| ***Lasiodiplodia thailandica*** | **CBS138760** | **OR759966** | **OR767664** |
| *Lasiodiplodia thailandica* | MFLUCC20-0200 | **OM919715** | – |
| *Lasiodiplodia thailandica* | MFLUCC20-0199 | **ON000546** | **OP099911** |
| ***Lasiodiplodia theobromae*** | **CBS_164_96_** | **NG_062745** | – |
| *Lasiodiplodia theobromae* | CBS111530 | FJ150695 | – |
| *Lasiodiplodia theobromae* | CBS124.13 | EU673195 | DQ458875 |
| *Lasiodiplodia tropica* | CGMCC3.18477 | KY783454 | KY848616 |
| ***Lasiodiplodia venezuelensis*** | **CBS_118739** | **EU673192** | **EU673305** |
| *Lasiodiplodia venezuelensis* | CBS129757 | MH865373 | JX545122 |
| *Lasiodiplodia viticola* | CBS128313 | MH864855 | – |
| *Lasiodiplodia viticola* | CBS128315 | MH864856 | – |
| ***Lasiodiplodia vitis*** | **CBS124060_** | **KX464148** | **KX464642** |
| **Outgroup** |  |  |  |
| *Diplodia mutila* | CMW7060 | KF766158 | – |
| *Diplodia seriata* | CBS112555 | KF766161 | AY573220 |

|  |  |
| --- | --- |
|  | **Table 3.** Taxa names, strain numbers and GenBank accession numbers of the ITS, *tef1-α* and *act* and |
|  | sequences used in the phylogenetic analyses of *Cladosporiaceae*. The newly generated sequences are |
|  | indicated in red, while the type strains are in black bold font. “–” indicates unavailable sequences. |
|  |  |

| **Taxon name** | **Voucher/Culture** | **Gene accession numbers** | | |
| --- | --- | --- | --- | --- |
|  |  | **ITS** | *tef1-α* | *act* |
| *Cercospora beticola* | CBS 116456 | NR_121315 | AY840494 | AY840458 |
| ***Cladosporium acalyphae*** | **CBS 125982** | **HM147994** | **HM148235** | **HM148481** |
| ***C. alboflavescens*** | **CBS 140690** | **LN834420** | **LN834516** | **LN834604** |
| ***C. angustisporum*** | **CBS 125983** | **HM147995** | **HM148236** | **HM148482** |
| *C. angustisporum* | UTHSC-DI-13-240 | LN834356 | LN834452 | LN834540 |
| *C. anthropophilum* | CBS 117483 | HM148007 | HM148248 | HM148494 |
| ***C. anthropophilum*** | **CBS 140685** | **LN834437** | **LN834533** | **LN834621** |
| *C. anthropophilum* | KUMCC 19-0182 | MN412638 | MN417513 | MN417511 |
| *C. anthropophilum* | KUMCC 19-0202 | MN412639 | MN417514 | MN417512 |
| *C. anthropophilum* | UTHSC-DI-13-168 | LN834407 | LN834503 | LN834591 |
| *C. anthropophilum* | UTHSC-DI-13-169 | LN834408 | LN834504 | LN834592 |
| *C. anthropophilum* | UTHSC-DI-13-178 | LN834410 | LN834506 | LN834594 |
| *C. anthropophilum* | UTHSC-DI-13-179 | LN834411 | LN834507 | LN834595 |
| *C. anthropophilum* | UTHSC-DI-13-207 | LN834413 | LN834509 | LN834597 |
| *C. anthropophilum* | UTHSC-DI-13-226 | LN834421 | LN834517 | LN834605 |
| *C. anthropophilum* | UTHSC-DI-13-228 | LN834423 | LN834519 | LN834607 |
| *C. anthropophilum* | UTHSC-DI-13-244 | LN834428 | LN834524 | LN834612 |
| *C. anthropophilum* | UTHSC-DI-13-246 | LN834430 | LN834526 | LN834614 |
| *C. anthropophilum* | UTHSC-DI-13-269 | LN834437 | LN834533 | LN834621 |
| *C. anthropophilum* | UTHSC-DI-13-271 | LN834439 | LN834535 | LN834623 |
| *C. asperulatum* | CBS 126339 | HM147997 | HM148238 | HM148484 |
| ***C. asperulatum*** | **CBS 126340** | **HM147998** | **HM148239** | **HM148485** |
| ***C. australiense*** | **CBS 125984** | **HM147999** | **HM148240** | **HM148486** |
| *C. austroafricanum* | CPC 16763 | KT600381 | KT600478 | KT600577 |
| ***C. chalastosporoides*** | **CBS 125985** | **HM148001** | **HM148242** | **HM148488** |
| *Cladosporium chalastosporoides* | CPC 13864 | NR_119838 | LN834504 | FJ936165 |
| ***C. chubutense*** | **CBS 124457** | **FJ936158** | **FJ936161** | **HM148490** |
| ***C. cladosporioides*** | **CBS 112388** | **HM148003** | **HM148244** | **HM148491** |
| *C. cladosporioides* | CBS 113738 | HM148004 | HM148245 | HM148533 |
| *C. cladosporioides* | CPC 14292 | HM148046 | HM148287 | LN834544 |

| *C. cladosporioides* | UTHSC-DI-13-215 | LN834360 | LN834456 | HM148554 |
| --- | --- | --- | --- | --- |
| *C. colocasiae* | CBS 119542 | HM148066 | HM148309 | HM148555 |
| ***C. colocasiae*** | **CBS 386.64** | **HM148067** | **HM148310** | **FJ936166** |
| ***C. colombiae*** | **CBS 274.80B** | **FJ936159** | **FJ936163** | **FJ936165** |
| ***C. crousii*** | **CBS140686** | **LN834431** | **LN834527** | **LN834615** |
| ***C. cucumerinum*** | **CBS 171.52** | **HM148072** | **HM148316** | **HM148561** |
| *C. cucumerinum* | CBS 173.54 | HM148074 | HM148318 | HM148563 |
| *C. delicatulum* | CBS 126342 | HM148079 | HM148323 | HM148568 |
| ***C. delicatulum*** | **CBS 126344** | **HM148081** | **HM148325** | **HM148570** |
| ***C. dracaenae*** | **MFLUCC180919** | **OM908927** | **OP099913** | **-** |
| ***C. dracaenicola*** | **MFLUCC180915** | **OM908928** | **-** | **-** |
| ***C. exile*** | **CBS 125987** | **HM148091** | **HM148335** | **HM148580** |
| ***C. flabelliforme*** | **CBS 126345** | **HM148092** | **HM148336** | **HM148581** |
| *C. flabelliforme* | UTHSC-DI-13-267 | LN834361 | LN834457 | LN834545 |
| *C. flavovirens* | UTHSC-DI-13-273 | LN834440 | LN834536 | LN834624 |
| *C. funiculosum* | CBS 122128 | HM148093 | HM148337 | HM148582 |
| ***C. funiculosum*** | **CBS 122129** | **HM148094** | **HM148338** | **HM148583** |
| ***C. gamsianum*** | **CBS 125989** | **HM148095** | **HM148339** | **HM148584** |
| *C. globisporum* | CBS 812.96 | HM148096 | HM148340 | HM148585 |
| ***C. grevilleae*** | **CBS 114271** | **JF770450** | **JF770472** | **JF770473** |
| ***C. hillianum*** | **CBS 125988** | **HM148097** | **HM148341** | **HM148586** |
| *C. inversicolor* | CBS 143.65 | HM148100 | HM148344 | HM148589 |
| *C. ipereniae* | CBS 140483 | KT600394 | KT600491 | KT600589 |
| *C. ipereniae* | CPC 16855 | KT600395 | KT600492 | KT600590 |
| ***C. iranicum*** | **CBS 126346** | **HM148110** | **HM148354** | **HM148599** |
| *C. limoniforme* | CBS 113737 | KT600396 | KT600493 | KT600591 |
| ***C. longicatenatum*** | **CPC 17189** | **KT600403** | **KT600500** | **KT600598** |
| ***C. lycoperdinum*** | **CBS 126347** | **HM148112** | **HM148356** | **HM148601** |
| ***C. lycoperdinum*** | **CBS 574.78C** | **HM148115** | **HM148359** | **HM148604** |
| ***C. montecillanum*** | **CPC 15605** | **KT600407** | **KT600505** | **KT600603** |
| ***C. montecillanum*** | **CPC 17953** | **KT600406** | **KT600504** | **KT600602** |
| ***C. myrtacearum*** | **CBS 126350** | **HM148117** | **HM148361** | **HM148606** |
| ***C. oxysporum*** | **CBS 125991** | **HM148118** | **HM148362** | **HM148607** |
| ***C. oxysporum*** | **CBS 126351** | **HM148119** | **HM148363** | **HM148608** |
| ***C. paracladosporioides*** | **CBS 171.54** | **HM148120** | **HM148364** | **HM148609** |
| ***C. parapenidielloides*** | **CPC 17193** | **KT600410** | **KT600508** | **KT600606** |
| ***C. phaenocomae*** | **CBS 128769** | **JF499837** | **JF499875** | **JF499881** |
| *C. phyllactiniicola* | CBS 126353 | HM148151 | HM148395 | HM148640 |
| ***C. phyllactiniicola*** | **CBS 126355** | **HM148153** | **HM148397** | **HM148642** |
| ***C. phyllophilum*** | **CBS 125992** | **HM148154** | **HM148398** | **HM148643** |
| *C. phyllophilum* | CBS 125990 | HM148111 | HM148355 | HM148600 |
| ***C. pini-ponderosae*** | **CBS 124456** | **FJ936160** | **FJ936164** | **FJ936167** |
| ***C. pseudochalastosporoides*** | **CPC 17823** | **KT600415** | **KT600513** | **KT600611** |
| ***C. pseudocladosporioides*** | **CBS 125993** | **HM148158** | **HM148402** | **HM148647** |
| *C. pseudocladosporioides* | CBS 667.80 | HM148165 | HM148409 | HM148654 |
| *C. pseudocladosporioides* | CPC 13683 | HM148173 | HM148417 | HM148662 |
| *C. pseudocladosporioides* | CPC 14020 | HM148185 | HM148429 | HM148674 |
| *C. pseudocladosporioides* | CPC 14295 | HM148188 | HM148432 | HM148677 |
| *C. pseudocladosporioides* | UTHSC-DI-13-165 | LN834406 | LN834502 | LN834590 |
| *C. pseudocladosporioides* | UTHSC-DI-13-190 | LN834412 | LN834508 | LN834596 |
| *C. pseudocladosporioides* | UTHSC-DI-13-210 | LN834414 | LN834510 | LN834598 |
| *C. pseudocladosporioides* | UTHSC-DI-13-218 | LN834418 | LN834514 | LN834602 |
| *C. pseudocladosporioides* | UTHSC-DI-13-227 | LN834422 | LN834518 | LN834606 |
| *C. pseudocladosporioides* | UTHSC-DI-13-234 | LN834424 | LN834520 | LN834608 |
| *C. pseudocladosporioides* | UTHSC-DI-13-238 | LN834426 | LN834522 | LN834610 |
| *C. pseudocladosporioides* | UTHSC-DI-13-241 | LN834427 | LN834523 | LN834611 |
| *C. pseudocladosporioides* | UTHSC-DI-13-245 | LN834429 | LN834525 | LN834613 |
| *C. pseudocladosporioides* | UTHSC-DI-13-251 | LN834432 | LN834528 | LN834616 |

| *C. pseudocladosporioides* | UTHSC-DI-13-261 | LN834384 | LN834480 | LN834568 |
| --- | --- | --- | --- | --- |
| *C. pseudocladosporioides* | UTHSC-DI-13-265 | LN834435 | LN834531 | LN834619 |
| *C. pseudocladosporioides* | UTHSC-DI-13-268 | LN834436 | LN834532 | LN834620 |
| *C. pseudocladosporioides* | UTHSC-DI-13-270 | LN834438 | LN834534 | LN834622 |
| ***C. rectoides*** | **CBS 125994** | **HM148193** | **HM148438** | **HM148683** |
| *C. ruguloflabelliforme* | CPC 19707 | KT600458 | KT600557 | KT600655 |
| *C. scabrellum* | **CBS 126358** | HM148195 | HM148440 | HM148685 |
| *Cladosporium scabrellum* | CPC 14976 | NR_119853 | – | – |
| *Cladosporium scabrellum* | UFMGCB 13238 | MT373238 | – | – |
| ***C. silenes*** | **CBS 109082** | **EF679354** | **EF679429** | **EF679506** |
| ***C. subinflatum*** | **CBS 121630** | **EF679389** | **EF679467** | **EF679543** |
| *C. subinflatum* | CBS 121630 | EF679389 | EF679467 | EF679543 |
| ***C. subuliforme*** | **CBS 126500** | **HM148196** | **HM148441** | **HM148686** |
| *C. tenuissimum* | CPC 13222 | HM148210 | HM148455 | HM148700 |
| *C. tenuissimum* | CPC 14250 | HM148211 | HM148456 | HM148701 |
| *C. tenuissimum* | UTHSC-DI-13-258 | LN834404 | LN834500 | LN834588 |
| ***C. varians*** | **CBS 126362** | **HM148224** | **HM148470** | **HM148715** |
| ***C. verrucocladosporioides*** | **CBS 126363** | **HM148226** | **HM148472** | **HM148717** |
| *C. versiforme* | CPC 19053 | KT600417 | KT60051 | – |
| ***C. xantochromaticum*** | **CBS 140691** | **LN834415** | **LN834511** | **LN834599** |
| ***C. xylophilum*** | **CBS 125997** | **HM148230** | **HM148476** | **HM148721** |
| **Outgroup** |  |  |  |  |
| *Cladosporium herbarum* | CBS 121621 | NG_069890 | – | – |

**Table 4.** Taxa names, strain numbers and GenBank accession numbers of the LSU, ITS and *act* sequences used in the phylogenetic analyses of *Mycosphaerellaceae*. The newly generated sequences are indicated in red, while the type strains are in black bold font. “–” indicates unavailable sequences.

| **Taxon name** | **Voucher/Culture** | **GenBank Accession Number** | | |
| --- | --- | --- | --- | --- |
|  |  | **LSU** | **ITS** | *act* |
| ***Z. aerohyalinosporum*** | **CBS125011= CPC 14636** | **KF901930** | **KF901605** | **KF903576** |
| ***Z. anthuriicola*** | **CBS 118742** | **FJ839662** | **–** | **–** |
| *Z. citri* | CBS 116366 = CPC 10522 = CMW  11730 | KF902138 | KF901780 | – |
|  | CBS 116426 | KF901987 | KF901648 | – |
|  | CBS 122455 | KF902156 | KF901797 | – |
|  | CPC 10522 | KF902136 | KF901778 | – |
|  | CPC 13467 | KF902137 | KF901779 | KF903650 |
|  | CPC 15285 | KF902150 | KF901791 | – |
|  | CPC 15289 | KF902151 | KF901792 | – |
|  | CPC 15291 | KF902152 | KF901793 | KF903676 |
|  | CPC 15293 | KF902153 | KF901794 | – |
|  | CPC 15294 | KF902154 | KF901795 | – |
|  | CPC 15296 | KF902155 | KF901796 | – |
|  | CPC 15300 | KF902086 | KF901739 | KF903576 |
| ***Z. citrigriseum*** | **MFLUCC18-0903** | **OM919720** | **OM919719** | **OP099914** |
| ***Z. eucalypti*** | **CBS 121101 = CPC 13302** | **KF901931** | **KF901606** | **KF903642** |
| ***Z. eucalyptorum*** | **CBS 118500 = CPC 11174** | **–** | **KF901652** | **KF903495** |
| ***Z. lonicericola*** | **CBS 125008 = CPC 11671** | **KF902093** | **–** | **–** |
| ***Z. nabiacense*** | **CBS 125010 = CPC 12748** | **KF901933** | **KF901608** | **KF903575** |
|  | CPC 12748 | KF901932 | KF901607 | KF903624 |
| ***Z. nocoxi*** | **CBS 125009 = CPC 14044** | **KF902157** | **–** | **–** |
| ***Z. parkii*** | **CBS 387.92 = CPC 353** | **KF902143** | **KF901785** | **KF903585** |
| *Z. pseudoparkii* | CBS 110988 = CPC 1090 | KF901975 | KF901640 | KF903418 |
|  | **CBS 110999 = CPC 1087** | **KF901977** | **KF901642** | **KF903419** |
|  | CBS 111049 = CPC 1089 | KF901976 | KF901641 | KF903426 |
| ***Z. xenoparkii*** | **CBS 111185 = CPC 1300** | **KF902002** | **KF901663** | **KF903438** |

| *Zymoseptoria verkleyi* | CBS 133618 | KC005802 | KF901606 | – |
| --- | --- | --- | --- | --- |
| **Outgroup** |  |  |  |  |
| ***Pseudozamidium eucalypti*** | **CBS 121101** | **KF901931** | **KF901606** | **KF903642** |

**Table 5.** Taxa names, strain numbers and GenBank accession numbers of the LSU sequences used in the phylogenetic analyses of *Didymellaceae*. The newly generated sequences are indicated in red, while the type strains are in black bold font. “–” indicates unavailable sequences

| **Taxon name** | **Voucher/Culture** | **Gene accession numbers** |
| --- | --- | --- |
|  |  | **LSU** |
| *Boeremia exigua var. exigua* | CBS 431.74, PD 74/2447 | EU754183 |
| ***Coniothyrium***  ***clematidisrectae*** | **CBS 507.63, PD 07/03486747** | **FJ515647** |
|  | PD 95/1958 | FJ515648 |
| ***Didymella maydis*** | **CBS 588.69** | **EU754192** |
| *Didymella vitalbina* | CBS 454.64 | FJ515646 |
| ***Longididymella clematidis*** | **CBS123705** | **MH874852** |
| *Longididymella clematidis* | MFLUCC 22–0099 | ON000548 |
| ***Longididymella vitabina*** | **CBS123707** | **MH874853** |
| *Longididymella vitabina* | CBS123706 | MN943687 |
| *Longididymella vitabina* | CBS454.64 | MH870116 |
| *Longididymella vitabina* | CBS 911.87 | FJ515633 |
| *Longididymella vitabina* | PD 75/294 | FJ515637 |
|  | PD 80/683 | FJ515638 |
|  | PD 91/1865 | FJ515639 |
|  | PD 95/895 | FJ515640 |
|  | PD 97/12061 | FJ515641 |
|  | PD 97/12062 | FJ515642 |
|  | PD 99/2069 | FJ515643 |
| *Longididymella vitabina* | PD 08/04373904.2B | FJ515644 |
| *Longididymella vitabina* | PD 08/04417700.3 | FJ515645 |
| *Phoma clematidina* | CBS 201.49 | FJ515628 |
|  | CBS 195.64 | FJ515629 |
|  | CBS 102.66 | FJ515630 |
|  | CBS 520.66, PD 64/657 | FJ515631 |
|  | **CBS 108.79, PD 78/522** | **FJ515632** |
| *Phoma complanata* | CBS 268.92, PD 75/3 | EU754180 |
| *Phoma glaucii* | CBS 114.96, PD 94/888 | FJ515649 |
| *Phoma glomerata* | CBS 528.66, PD 63/590 | EU754184 |
| ***Phoma herbarum*** | **CBS 615.75** | **EU754186** |
| **Outgroup** |  |  |
| ***Calophoma complanata*** | **CBS 268.92** | **EU754180** |

**Table 6.** Taxa names, strain numbers and GenBank accession numbers of the ITS and *tef1-α* sequences used in the phylogenetic analyses of *Pleosporaceae*. The newly generated sequences are indicated in red, while the type strains are in black bold font. “–” indicates unavailable sequences

| **Taxon name** | **Voucher/Culture** | **GenBank Accession Number** | |
| --- | --- | --- | --- |
|  |  | **ITS** | *tef1-α* |
| *Bipolaris bicolor* | CBS 690.96 | KJ909762 | KM093776 |
| *B. brachiariae* | CPC28819 | NR_153654 | MF490850 |
| *B. chloridis* | CBS 242.77 | JN192372 | – |
| ***B. clavata*** | **BRIP 12530** | **KJ415524** | **KJ415471** |
| *B. coffeana* | MFLUCC 17-2600 | OP090572 | OP099915 |

| ***B. coffeana*** | **BRIP 14845** | **KJ415525** | **KJ415470** |
| --- | --- | --- | --- |
|  | C 12.04 | KM230385 | KM093781 |
|  | MFLUCC 12-0185 | KJ922385 | KM093784 |
|  | M 1129 | KJ922384 | KM093780 |
|  | M 1130 | KM230387 | KM093779 |
|  | MFU0090 | KM230386 | KM093783 |
|  | ICMP 6128 | JX256412 | JX266581 |
| *B. cookei* | AR 5185 | KJ922391 | KM093777 |
|  | MAFF 51191 | KJ922392 | KM093778 |
| *B. crotonis (= B. eleusines)* | CBS 274.91 | KJ909768 | KM093758 |
| *B. crotonis* | BRIP 14838 | KJ415526 | KJ415479 |
| ***B. cynodontis*** | **CBS 109894** | **KJ909767** | **KM093782** |
| *B. drechsleri* | CBS 136207 | KF500530 | KM093760 |
|  | MUS0028 | KF500532 | KM093761 |
|  | FIP 373 | KF500531 | KM093759 |
| ***B. eleusines*** | **CBS274.91** | KJ909768 | – |
| ***B. heliconiae*** | **BRIP 17186** | **KJ415530** | **KJ415465** |
| ***B. heveae*** | **CBS 241.92** | **KJ909763** | **KM093791** |
| ***B. gossypina*** | **BRIP 14840** | **KJ415528** | **KJ415467** |
| *B. luttrellii* | BRIP 14643 | AF071350 | – |
| *B. maydis* | CBS 137271/ C5 | AF071325 | KM093794 |
|  | AR 5182 | KM230388 | KM093792 |
|  | AR 5183 | KM230390 | KM093796 |
|  | M 1122/ C4 | KM230389 | KM093795 |
|  | CBS 136.29 | KJ909769 | KM093793 |
| *B. microconidia* | CGMCC3.19336 | MN215630 | MN263924 |
| *B. microlaenae* | BRIP 15613 | JN601032 | JN601017 |
| *B. microstegii* | CBS 132550 | JX089579 | KM093756 |
|  | AR 5192 | KM230391 | KM093757 |
| ***B. omanensis*** | **SQUCC13928** | MK072962 | – |
| ***B. oryzae*** | **MFLUCC 100715** | **JX256416** | **JX266585** |
|  | MFLUCC 100733 | JX256417 | KM093790 |
|  | MAFF 235499 | KJ922383 | KM093789 |
|  | AR3797 | KM230392 | KM093786 |
|  | AR 5204 | KM230393 | KM093787 |
| ***B. panici-miliacei*** | **CBS 199.29** | **KJ909773** | **KM093788** |
| *B. peregianensis* | DAOM 221998 | KJ922393 | KM093797 |
|  | BRIP 12790 | JN601034 | JN601022 |
| ***B. pluriseptata*** | **BRIP 14839** | **KJ415532** | **KJ415461** |
| *B. sacchari* | ICMP 6227 | KJ922386 | KM093785 |
| *B. salkadehensis* | Bi 4 | AB675491 | – |
| ***B. salviniae*** | **IMI 228224** | **KJ922390** | **KM093772** |
| *B. salviniae (= B. melinidis)* | BRIP 12898 | JN601035 | KM093771 |
| ***B. secalis*** | **BRIP 14453** | **KJ415537** | **KJ415455** |
| *B. setariae* | CPC28802 | **MW446193** | – |
| ***B. sorokiniana (= B. multiformis)*** | **CBS 120.24** | **KJ909776** | **KM093762** |
|  | CBS 110.14 | KJ922381 | KM093763 |
|  | FIP 499 | KJ922382 | KM093769 |
|  | MAFF 236448 | KJ909792 | KM093767 |
|  | MAFF 235500 | KJ909789 | KM093764 |
|  | MAFF 235501 | KJ909791 | KM093766 |
|  | MAFF 238877 | KJ909790 | KM093765 |
|  | CBS 480.74 | KJ909771 | KM093768 |
| ***B. urochloae*** | **ATCC 58317** | **KJ922389** | **KM093770** |
| ***B. variabilis*** | **CBS127716** | **KY905676** | **KY905696** |
| *B. victoriae* | CBS 327.64 | KJ909778 | KM093748 |
| ***B. woodii*** | **BRIP12239** | **KX452458** | **KX452475** |
| *B. yamadae* | DAOM 147441 | KJ922388 | KM093774 |

|  | MAFF 235507 | KJ922387 | KM093775 |
| --- | --- | --- | --- |
|  | **CBS 202.29** | **KJ909779** | **KM093773** |
| *B. zeae* | BRIP11512 | KJ909786 | KM093753 |
| *AR 5181* | S. bicolor | KM230394 | KM093754 |
|  | DAOM 211267 | KJ909787 | KM093755 |
| *B. zeicola* | AR 5166 | KJ909788 | KM093750 |
|  | AR 5168 | KM230397 | KM093751 |
|  | FIP 532 | KM230398 | KM093752 |
| ***Cochliobolus heterostrophus*** |  |  |  |
| ***Cochliobolus helicconiae*** | BRIP17189 | KJ415530 | KJ415465 |
| ***Cochliobolus luttrellii*** | BRIP14643 | AF071350 | KJ415464 |
| ***Cochliobolus microlaenae*** | CBS280.91 | JN601032 | JN601018 |
| **Outgroup** |  |  |  |
| ***C. lunata*** | **CBS 730.96** | **JX256429** | – |
| *C. lunata* | CBS 157.34 | JX256430 | – |

|  | **Table 7.** Taxa names, strain numbers and GenBank accession numbers of the ITS sequences used in |
| --- | --- |
|  | the phylogenetic analyses of *Pleosporaceae*. The newly generated sequences are indicated in red, while |
|  | the type strains are in black bold font. “–” indicates unavailable sequences. |
|  |  |

| **Taxon name** | **Voucher/Culture** | **Gene accession numbers** |
| --- | --- | --- |
|  |  | **ITS** |
| *B. drechsleri* | MUS0028 | KF500532 |
| ***C. aeria*** | **CBS 294.61** | **HE861850** |
| ***C. affinis*** | **CBS 154.34** | **KJ909780** |
| *C. affinis* | CBS 185.49 | HG778982 |
| *C. akaii* | CBS 317.86 | KJ909782 |
| *C. akaiiensis* | BRIP 16080 | KJ415539 |
| ***C. alcornii*** | **MFLUCC 100703** | **JX256420** |
| *C. americana* | UTHSC 072649 | HE861834 |
| *C. americana* | UTHSC 08278 | HE861832 |
| *C. asianensis* | MFLUCC 100685 | JX256425 |
| ***C. asianensis*** | **MFLUCC 100711** | **JX256424** |
| *C. australiensis* | BRIP 12044 | KJ415540 |
| *C. australiensis* | CBS 172.57 | JN601026 |
| *C. australis* | BRIP 12247a | KC424609 |
| *C. australis* | BRIP 12521 | KJ415541 |
| ***C. bannonii*** | **BRIP 16732** | **KJ415542** |
| *C. bannonii* | DAOM196762 | KP400634 |
| *C. borreriae* | AR5176r | KP400637 |
| *C. borreriae* | MFLUCC 11–0422 | KP400638 |
| ***C. bothriochloae*** | **BRIP 12522** | **KJ415543** |
| *C. brachyspora* | CBS 186.50 | KJ922372 |
| *C. brachyspora* | ZW020185 | HM053667 |
| ***C. buchloes*** | **CBS 246.49** | **KJ909765** |
| ***C. caricapapayae*** | **CBS 135941** | **HG778984** |
| ***C. chlamydospora*** | **UTHSC 072764** | **HG779021** |
| *C. clavata* | BRIP:61680 | KU552205 |
| ***C. crustacea*** | **BRIP 13524** | **KJ415544** |
| *C. dactyloctenicola* | CPC 28810 | MF490815 |
| ***C. ellisii*** | **CBS 193.62** | **JN192375** |
| *C. ellisii* | IMI 75862 | KJ922379 |
| *C. eragrostidis* | CBS 189.48 | HG778986 |
| *C. geniculata* | CBS 187.50 | KJ909781 |
| *C. gladioli* | CBS 210.79 | HG778987 |
| *C. gladioli* | ICMP 6160 | JX256426 |
| ***C. graminicola*** | **BRIP 23186a** | **JN192376** |
| ***C. harveyi*** | **BRIP 57412** | **KJ415546** |
| ***C. hawaiiensis*** | **BRIP 11987** | **KJ415547** |

| ***C. heteropogonicola*** | **BRIP 14579** | **KJ415548** |
| --- | --- | --- |
| ***C. heteropogonis*** | **CBS 284.91** | **JN192379** |
| *C. heteropogonis* | CBS 511.91 | HF934918 |
| *C. hominis* | AR 5118 | KP400639 |
| *C. hominis* | MFLUCC 120191 | KP400640 |
| *C. homomorpha* | CBS 156.60 | JN192380 |
| ***C. inaequalis*** | **CBS 102.42** | **KJ922375** |
| *C. inaequalis* | DAOM 20022 | KJ922374 |
| *C. intermedius* | CBS 334.64 | HG778991 |
| *C. intermedius* | UTHSC 09–3240 | HE861855 |
| *C. ischaemi* | CBS 630.82 | JX256428 |
| ***C. ischaemi*** | **ICMP 6172** | **JX256428** |
| ***C. lunata*** | **CBS 730.96** | **JX256429** |
| *C. lunata* | CBS 157.34 | JX256430 |
| *C. lunata* | MFLUCC 22-0076 | OP090556 |
| *C. malina* | CBS 131274 | JF812154 |
| *C. malina* | FLS–119 | KR493070 |
| ***C. miyakei*** | **CBS197.29** | **KJ909770** |
| *C. mosaddeghii* | IRAN 3131C | MG846737 |
| *C. mosaddeghii* | IRAN 3123C | MG971270 |
| *C. muehlenbeckiae* | AR5180 | KP400649 |
| *C. muehlenbeckiae* | MUS 0031 | KP400647 |
| ***C. neergaardii*** | **BRIP 12919** | **KJ415550** |
| *C. neergaardii* | DAOM 228085 | KJ909784 |
| *C. neoindica* | BRIP 17439 | AF081449 |
| *C. nicotiae* | BRIP 11983 | KJ415551 |
| ***C. nicotiae*** | **CBS 655.74** | **KJ909772** |
| *C. nisikadoi* | CBS 192.29 | AF081447 |
| *C. nodosa* | CPC 28801 | MF490817 |
| *C. nodosa* | CPC 28812 | MF490818 |
| ***C. nodulosa*** | **CBS 160.58** | **JN601033** |
| ***C. oryzae*** | **CBS 169 53** | **KP400650** |
| *C. ovariicola* | BRIP 15882 | JN601031 |
| *C. ovariicola* | CBS 286.91 | HG778994 |
| ***C. pallescens*** | **CBS 156.35** | **KJ922380** |
| *C. papendorfii* | BRIP 57608 | KJ415552 |
| ***C. papendorfii*** | **CBS308.67** | **KJ909774** |
| ***C. perotidis*** | **CBS 350.90** | **JN192385** |
| *C. pisi* | CBS 190.48 | KY905678 |
| *C. portulacae* | BRIP 14837 | KJ415554 |
| *C. portulacae* | CBS 239.48 | KJ909775 |
| ***C. prasadii*** | **CBS 143.64** | **KJ922373** |
| *C. prasadii* | CBS 144.64 | HG778997 |
| *C. protuberata* | 5876 | KT012665 |
| ***C. protuberata*** | **CBS 376.65** | **KJ922376** |
| *C. pseudobrachyspora* | CPC 28808 | MF490819 |
| *C. pseudolunata* | UTHSC 092092 | HE861842 |
| *C. pseudorobusta* | UTHSC 083458 | HE861838 |
| ***C. ravenelii*** | **BRIP 13165** | **JN192386** |
| *C. ravenelii* | CBS 127709 | HG778999 |
| ***C. richardiae*** | **BRIP 4371** | **KJ415555** |
| ***C. robusta*** | **CBS624 68** | **KJ909783** |
| *C. ryleyi* | BRIP 12554 | KJ415556 |
| *C. ryleyi* | CBS349.90 | KJ909766 |
| *C. senegalensis* | CBS 149.71 | HG779001 |
| *C. senegalensis* | ZM020571 | JN006787 |
| *C. soli* | CBS 222.96 | KY905679 |
| *C. sorghina* | BRIP 15900 | KJ415558 |

| *C. spicifera* | CBS 274.52S | JN192387 |
| --- | --- | --- |
| *C. subpapendorfii* | CBS656.74 | KJ909777 |
| *C. trifolii* | AR5169 | KP400656 |
| *C. tripogonis* | BRIP 12375 | JN192388 |
| *C. tropicalis* | BRIP 14834 | KJ415559 |
| *C. tsudae* | BRIP 10970 | KC424605 |
| *C. tsudae* | MAFF 236750 | KP400651 |
| *C. tuberculata* | CBS 14663 | JX256433 |
| *C. uncinata* | CBS 221.52 | HG779024 |
| *C. variabilis* | CPC 28813 | MF490820 |
| *C. variabilis* | CPC 28815 | NR154866 |
| *C. verruculosa* | CBS150 63 | KP400652 |
| *C. verruculosa* | MFLUCC 100690 | JX256437 |
| *Curvularia sp.* | AR5117 | KP400655 |
| *Curvularia sp.* | MFLUCC 100709 | JX256442 |
| *Curvularia sp.* | MFLUCC 100739 | JX256443 |
| *Curvularia sp.* | MFLUCC 120177 | KP400654 |
| *Curvularia sp.* | UTHSC 08809 | HE861826 |
| **Outgroup** |  |  |
| ***C. coicis*** | **CBS 192.29** | **JN192373** |

|  |  |
| --- | --- |
|  | **Table 8.** Taxa names, strain numbers and GenBank accession numbers of the ITS, LSU, SSU and *rpb2* |
|  | sequences used in the phylogenetic analyses of *Torulaceae*. The newly generated sequences are |
|  | indicated in red, while the type strains are in black bold font. “–” indicates unavailable sequences |

| **Taxon name** | **Voucher/Culture** | **Gene accession numbers** | | | |
| --- | --- | --- | --- | --- | --- |
|  |  | **ITS** | **LSU** | **SSU** | ***rpb2*** |
| ***Torula acaciae*** | **CPC 29737** | **NR_155944** | **NG_059764** | **–** | **KY173594** |
| *Torula aquatica* | DLUCC 0550 | MG208166 | MG208145 | – | MG207976 |
| ***Torula aquatica*** | **MFLUCC16-1115** | **MG208167** | **MG208146** | **–** | **MG207977** |
| ***Torula breviconidiophora*** | **KUMCC 18–0130** | **MK071670** | **MK071672** | **MK071697** | **–** |
| *Torula camporesii* | KUMCC 19–0112 | MN507400 | MN507402 | MN507401 | MN507404 |
| ***Torula chiangmaiensis*** | **KUMCC 16–0039** | **MN061342** | **KY197856** | **KY197863** | **–** |
| ***Torula chromolaenae*** | **KUMCC 16–0036** | **MN061345** | **KY197860** | **KY197867** | **KY197873** |
| *Torula dracaenae* | MFLUCC 22–0135 | OM911931 | OM911934 | – | – |
| ***Torula fici*** | **CBS 595.96** | **KF443408** | **KF443385** | **KF443387** | **KF443395** |
| *Torula fici* | KUMCC 15–0428 | MG208172 | MG208151 | – | MG207981 |
| *Torula fici* | KUMCC 16–0038 | MN061341 | KY197859 | KY197866 | KY197872 |
|  | **MFLUCC 17–**  **0234** |  |  |  | **–** |
| ***Torula gaodangensis*** |  | **MF034135** | **NG_059827** | **NG_063641** |  |
| *Torula goaensis* | NFCCL 4040 | NR_159045 | NG_060016 | – | – |
| ***Torula herbarum*** | **CPC 24414** | **KR873260** | **KR873288** | **–** | **–** |
| ***Torula hollandica*** | **CBS 220.69** | **NR_132893** | **NG_064274** | **KF443389** | **KF443393** |
| ***Torula hydei*** | **KUMCC 16–0037** | **MN061346** | **MH253926** | **MH253928** | **–** |
| ***Torula mackenziei*** | **MFLUCC 13–**  **0839** | **MN061344** | **KY197861** | **KY197868** | **KY197874** |
| ***Torula masonii*** | **CBS 245.57** | **R_145193** | **NG_058185** | **–** | **–** |
| *Torula masonii* | DLUCC 0588 | MG208173 | MG208152 | – | MG207982 |
| *Torula masonii* | KUMCC 16–0033 | MN061339 | KY197857 | KY197864 | KY197870 |
| ***Torula pluriseptata*** | **MFLUCC14–0437** | **MN061338** | **KY197855** | **KY197862** | **KY197869** |
| ***Torula polyseptata*** | **KUMCC 18–0131** | **MK071671** | **MK071673** | **MK071698** | **–** |
| *Torula sp.* | CBS 246.57 | KF443411 | KR873290 | – | – |
| *Torula camporesii* | MFLU 20-0070 | MN507400 | MN507402 | MN507401 | MN507404 |
| **Outgroup** |  |  |  |  |  |
| *Dendryphion europaeum* | CPC 23231 | KJ869145 | KJ869202 | – | – |

|  | **Table 9.** Taxa names, strain numbers and GenBank accession numbers of the ITS, LSU, SSU and *rbp2* |
| --- | --- |
|  | sequences used in the phylogenetic analyses of *Sympoventuriaceae*. The newly generated sequences |
|  | are indicated in red, while the type strains are in black bold font. “–” indicates unavailable sequences |

| **Taxon name** | **Voucher/Culture** | **Gene accession numbers** | |  |
| --- | --- | --- | --- | --- |
|  |  | **LSU** | **ITS** | ***tub*** |
| ***O. anellii*** | **CBS 284.64** | **KF156138** | **FR832477** | **KF156184** |
| ***O. anomala*** | **CBS 131816** | **KF156137** | **HE575201** | **KF156194** |
| *O. crassihumicola* | CBS 120700 | KJ867430 | KJ867429 | KJ867433 |
| *O. constricta* | CBS 211.53 | KF156148 | HQ667519 | KF156187 |
| ***O. cordanae*** | **CBS 475.80** | **KF156122** | **KF156022** | **KF156197** |
| ***O. gamsii*** | **CBS 239.78** | **KF156150** | **KF156019** | **KF156190** |
| *O. globalis* | CBS 119643 | KF961096 | KF961085 | – |
| ***O. globalis*** | **CBS 119644** | **KF961097** | **KF961086** | **KF961065** |
| *O. globalis* | CBS 124172 | KF961098 | KF961087 | KF961066 |
| *O. globalis* | CBS 131956 | KF961100 | KF961088 | KF961067 |
| *O. globalis* | CBS 135766 | KF961106 | KF961094 | KF961072 |
| *O. globalis* | CBS 135921 | KF961101 | KF961089 | KF961068 |
| *O. globalis* | CBS 135922 | KF961102 | KF961090 | KF961069 |
| *O. globalis* | CBS 135923 | KF961103 | KF961091 | KF961084 |
| *O. globalis* | CBS 135924 | KF961104 | KF961092 | KF961070 |
| *O. globalis* | CBS 135925 | KF961105 | KF961093 | KF961071 |
| *O. globalis* | CBS 135926 | KF961099 | KF961095 | KF961073 |
| *O. globalis* | CBS 119643 | KF961096 | KF961085 | KF156195 |
| ***O. humicola*** | **CBS 116655** | **KF156124** | **HQ667521** | **KF156183** |
| ***O. lascauxensis*** | **CBS 131815** | **KF156136** | **FR832474** | **KF156182** |
| *O. longiphora* | CBS 435.76 | KF156135 | KF156038 | KF156191 |
| *O. macrozamiae* | CBS 102491 | KF156152 | KF156021 | KF156172 |
| ***O. minima*** | **CBS 510.71** | **KF156134** | **HQ667522** | **KF156171** |
| *O. musae* | CBS 729.95 | KF156144 | KF156029 | KF156189 |
| *O. musae* | MFLUCC22-0105 | ON159739 | ON159737 | ON184274 |
| ***O. sexualis*** | **CBS 131765** | **KF156118** | **KF156018** | **KF156180** |
| ***O. tshawytschae*** | **CBS 100438** | **KF156126** | **HQ667562** | **KF156185** |
| ***O. verrucosa*** | **CBS 383.81** | **KF156129** | **KF156015** | **KF156202** |
| **Outgroup** |  |  |  |  |
| ***V. calidifluminalis*** | **CBS 125818** | **KF156108** | **AB385698** | **KF156195** |

|  | **11 Data Availability Statement** |
| --- | --- |
|  | The datasets presented in this study can be found in online repositories. The names of the |
|  | repository/repositories and accession number(s) have been mentioned in the article/Supplementary |
|  | Material. |
|  |  |
